# Rapid within‐ and transgenerational changes in thermal tolerance and fitness in variable thermal landscapes

**DOI:** 10.1002/ece3.6496

**Published:** 2020-07-16

**Authors:** Grisel Cavieres, Enrico L. Rezende, Sabrina Clavijo‐Baquet, José M. Alruiz, Carla Rivera‐Rebella, Francisca Boher, Francisco Bozinovic

**Affiliations:** ^1^ Departamento de Ecología Center of Applied Ecology and Sustainability (CAPES) Pontificia Universidad Católica de Chile Santiago Chile; ^2^ Sección Etología Facultad de Ciencias Universidad de la República Montevideo Uruguay

**Keywords:** *Drosophila melanogaster*, fitness, phenotypic plasticity, thermal tolerance, thermal variability

## Abstract

Phenotypic plasticity may increase the performance and fitness and allow organisms to cope with variable environmental conditions. We studied within‐generation plasticity and transgenerational effects of thermal conditions on temperature tolerance and demographic parameters in *Drosophila melanogaster*. We employed a fully factorial design, in which both parental (P) and offspring generations (F1) were reared in a constant or a variable thermal environment. Thermal variability during ontogeny increased heat tolerance in P, but with demographic cost as this treatment resulted in substantially lower survival, fecundity, and net reproductive rate. The adverse effects of thermal variability (V) on demographic parameters were less drastic in flies from the F1, which exhibited higher net reproductive rates than their parents. These compensatory responses could not totally overcome the challenges of the thermally variable regime, contrasting with the offspring of flies raised in a constant temperature (C) that showed no reduction in fitness with thermal variation. Thus, the parental thermal environment had effects on thermal tolerance and demographic parameters in fruit fly. These results demonstrate how transgenerational effects of environmental conditions on heat tolerance, as well as their potential costs on other fitness components, can have a major impact on populations’ resilience to warming temperatures and more frequent thermal extremes.

## Graphical abstract

Within‐generation plasticity and transgenerational effects can ameliorate the impact of stressful thermal conditions on physiological and fitness‐related traits.

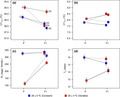

## 1 INTRODUCTION

Understanding the ecological consequences of changing environments and extreme climatic events has grown more prominent as climate change scenarios predict more frequent and pronounced fluctuations in temperature (Vázquez, Gianoli, Morris, & Bozinovic, 2017). The world's climate is changing dramatically, to such an extent that the 90% probability interval for global warming from 1990 to 2,100 predicts an increase in average temperatures ranging from 1.7°C to 4.9°C (IPCC, Rahmstorf & Coumou, 2011). And while it is acknowledged that warming temperatures will have a significant impact on biodiversity (Dawson, Jackson, House, Prentice, & Mace, 2011; Gitay, Suárez, Watson, & Dokken, 2002; Meehl & Tebaldi, 2004; Shuker, Simpkins, & Hero, 2016), recent studies have shown that the increase in climatic variability and thermal extremes may also have a major impact on populations through a decrease in growth rates, reproduction, and survival (Bozinovic, Medina, Alruiz, Cavieres, & Sabat, 2016; Folguera, Bastías, & Bozinovic, 2009).

In ectotherms, thermal performance is largely influenced by environmental conditions (Pörtner, 2002; Sunday, Bates, & Dulvy, 2011). Thermal variability and extremes temperatures can impose selection pressures on organisms and drive the evolution of physiological capabilities (Buckley & Huey, 2016; Diamond, Chick, Perez, Strickler, & Martin, 2018; Logan, Cox, & Calsbeek, 2014). Besides, experimental studies have shown that organisms are able to respond to thermal variability through plastic changes in thermal tolerance (Chidawanyika, Nyamukondiwa, Strathie, & Fischer, 2017; Terblanche, Nyamukondiwa, & Kleynhans, 2010). Within‐generation plasticity, which includes both developmental plasticity and reversible plasticity, might impact future generations accelerating the adaptation to novel or fluctuating environments (Ho & Burggren, 2010; Ezard, Prizak, & Hoyle, 2014). In contrast, transgenerational plasticity refers to phenotypic changes in the offspring generation, without DNA sequence alteration, as a response to environmental inputs experienced by the previous generation (Salinas, Brown, Mangel, & Munch, 2013; Donelson, Wong, Booth, & Munday, 2016). Transgenerational plasticity has been described in several traits, including locomotor performance (Cavieres, Alruiz, Medina, Bogdanovich, & Bozinovic, 2019; Leroi, Bennett, & Lenski, 1994; Seebacher, Beaman, & Little, 2014), thermal tolerance (Norouzitallab et al., 2014), and metabolic rate (Donelson, Munday, McCormick, & Pitcher, 2012; Le Roy, Loughland, & Seebacher, 2017), and might enables the offspring to change adaptively according to parental information and avoid the time lag between environmental signal and phenotypic response (Baker, Sultan, Maya, & Robin, 2019).

While heat extreme events can induce increased tolerance to high temperatures (Bozinovic et al., 2011; Estay, Clavijo‐Baquet, Lima, & Bozinovic, 2011), they may cause organisms to reduce energy allocation to reproduction (Ragland & Kingsolver, 2008; Roitberg & Mangel, 2016; Koussoroplis, Pincebourde, & Wacker, 2017). Indeed, it has been reported that prolonged exposure to extreme temperatures may cause a decrease in survival, fertility, and growth rate (Sikkink, Ituarte, Reynolds, Cresko, & Phillips, 2014; Sales et al., 2018; Cavieres et al., 2019) that can even affect the next generation (Guillaume, Monro, & Marshall, 2016). Royama (1992) proposed that the thermal environment can affect demographic parameters through nonlinear changes in fecundity and survival. Thus, high temperatures could negatively impact fitness (Clavijo‐Baquet et al., 2014; Estay et al., 2011) even if organisms exhibit a seemingly compensatory plastic response in thermal tolerance. Most experimental studies assessing the impact of thermal conditions on animal performance focus on physiological performance, and few studies report those effects in conjunction with Darwinian fitness (Bozinovic et al., 2011; Nyamukondiwa et al., 2018).

**FIGURE 1:**
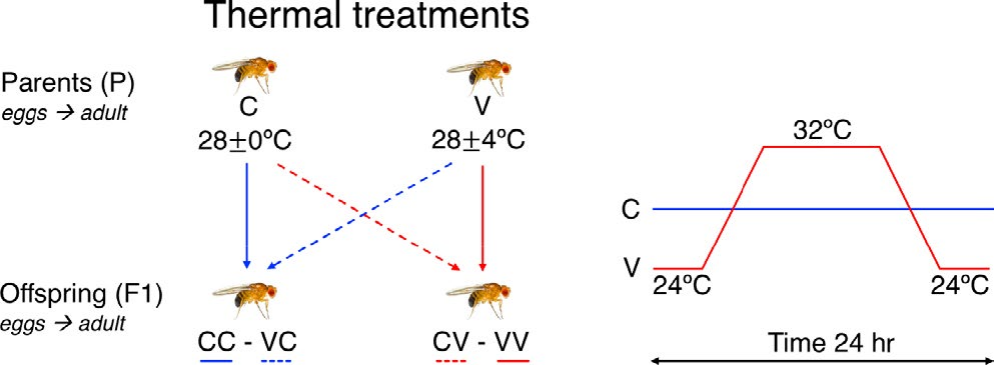
Schematic diagram of the experimental design used to assess within‐ and transgenerational effects of thermal variability on critical thermal limits, and demographic parameters in *D. melanogaster*. Offspring and parental generation were reared in one of two thermal environments, constant (C) or variable (V), described in the right panel (variable cycles included 8 hr at 24ºC, 8 hr at 32ºC, and 8 hr of ramping, see Methods). Abbreviations represent the thermal treatments for the parental generation (C and V) and the offspring (CC, CV, VV, and VC)

In this vein, here we quantified within‐generational plasticity and transgenerational effects of thermal environment on thermal tolerance and demographic parameters in the fruit fly *Drosophila melanogaster*, and their potential role to ameliorate the negative impact of increased temperature variability. Specifically, we evaluated critical thermal maximum and minimum (*CT_max_*, *CT_min_*) as indicators of physiological thermal tolerances, and the demographic parameters net reproductive rate (i.e., the average number of offspring produced by an individual during its lifetime, $R_{0}$), and generation time (i.e., the average time between the birth of a female and the birth of her first female offspring, $T_{g}$) as direct indicators of populational fitness (Pasztor, Meszéna, & Kisdi, 1996; Royama, 1992) (Figure 1).

Overall, we hypothesized that flies reared in variable environments would exhibit a trade‐off between physiological and fitness‐related traits, namely an increase in heat tolerance but with adverse effects on demographic parameters. We predicted that the negative effects on fitness might be buffered in the subsequent generation if the offspring encountered the same thermal environment as their parents.

## 2 MATERIALS AND METHODS

### 2.1 Experimental setup

We performed a cross‐factorial experiment in which parental flies (P) were raised in constant and variable thermal environments, and their offspring were split and maintained in either the parental environment or the opposite (Figure 1). To obtain this experimental design, we use more than 200 inseminated *D*.* melanogaster* collected in central Chile (33°26′S; 70°39′W at 500 m above sea level) during 2016 in a nearly 500‐m^2^ habitat. After collection, twenty groups were established with approximately ten females each. Groups were reared in controlled conditions at a constant ambient temperature *T_a_* = 24°C and a light:dark 12:12‐hr photoperiod. Flies were maintained for three generations in 250‐ml glass vials with the Burdick culture medium (Burdick, 1955). Third‐generation adult males and virgin females from this stock were randomly assigned to two thermal treatments that differ in the variance of temperature. Thermal treatments were 28 ± 0°C and 28 ± 4°C, a constant (C) and variable (V) thermal environment, respectively, and crossed under these conditions. Acclimation temperatures were chosen based on the well‐known limits of fruit fly egg viability (Hoffmann, 2010) (egg‐to‐adult viability is 80% at 28ºC and 0 to 5% at 32ºC; for details, see Ref. Cavieres, Bogdanovich, Toledo, & Bozinovic, 2018; Hoffmann, 2010). In the variable thermal environment, during the day, temperature started to increase linearly at 7:00, reached the maximum at 11:00, then stayed constant, and began to decrease at 19:00 and reached 24°C at 23:00 hr, and the heating/cooling rate between the minimum and maximum temperatures was 0.03°C/min (Figure 1). The offspring, which corresponds to the parental generation P in our breeding setup, were maintained from eggs to adult in each thermal treatment. Subsequently, adult males and virgin females from both treatments were evenly divided into two breeding groups and transferred to constant and variable thermal conditions. The breeding groups were allowed to interact for a period of 36 hr. Their offspring corresponds to the F1 in our breeding setup, resulting in a factorial experiment with two P (C and V for constant and variable, respectively) and four F1 groups (CC, CV, VV, and VC, which reflect both the parental and offspring thermal environments). As detailed below, using different individuals, we estimated for all P and F1 groups lower and upper thermal critical limits (*CT_min_* and *CT_max_*, respectively), and demographic parameters (net reproductive rate $\left(R_{0}\right)$ and generation time $\left(T_{g}\right)$.

### 2.2 Critical thermal limits

We quantified critical thermal limits in virgin flies 8–10 days, using the dynamic method (Bozinovic et al., 2016), which involves heating or cooling flies from a starting temperature until physiological failure (Terblanche, Deere, Clusella‐Trullas, Janion, & Chown, 2007). Flies were placed individually in 5‐mL glass vials into a thermoregulated bath, and the temperature was monitored employing a type K thermocouple inserted into a control empty vial. The flies were allowed to equilibrate for 10 min at either 19 or 28°C before either *CT_min_* or *CT_max_* assessments started, respectively. The cooling and heating rates were 0.1°C min/°C. We monitored flies every minute and recorded thermal limits as the temperature when postural control was lost. The point of critical thermal minimum (*CT_min_*) was defined as the temperature of loss of a coordinated muscle function, and critical thermal maximum (*CT_max_*) was defined as the temperature of onset of muscle spasms as suggested by Terblanche et al. (2007). Each thermal tolerance experiment was repeated at least three times to yield a minimum sample size of *N* = 45 (for details, see Figure S1).

### 2.3 Net reproductive rate and generation time

To quantify ontogenetic and transgenerational effects of thermal environment on $R_{0}$ and $T_{g}$, newly emerged adults from both in P and F1 were collected within 8 hr of hatching and transferred to vials containing 6 g of culture medium. Since temperature may impact fitness‐related traits and that effect could be mediated by population density (Clavijo‐Baquet et al., 2014; Estay et al., 2011; Royama, 1992), four different population densities were established following the discrete design of Utida (1941) and Royama (1992), which included two, four, eight, and sixteen individuals per vials (sex ratio 1:1). We prepared at least 7 glass vial (cohorts) per density, resulting in a minimum of 28 cohorts per experimental group. Every other day, vials were checked to determine the number of dead flies and to replace the culture medium until complete mortality of the cohort. We then counted the number of eggs from the removed medium to estimate daily fecundity. A Lotka life table (Carey, 2001) was constructed to estimate $R_{0}$ and $T_{g}$ combining data on fecundity ($m_{x}$) and the proportion of the surviving individuals at age $x\left(l_{x}\right)$ for each replicate. We estimated $R_{0}$ and $T_{g}$ as $R_{0}=\sum l_{x}m_{x}$ and $T_{g}=\sum xl_{x}m_{x}/R_{0}$.

### 2.4 Statistical analyses

Before the statistical tests, we evaluated the assumptions of normality and equality of variances using the Kolmogorov–Smirnov and Levene tests. To compare critical thermal limits and demographic variables among experimental groups, we included thermal treatment (*treat*) as a factor with six levels (C, V, CV, CC, VC, and VV) that describe the thermal experience of flies (direct experience through ontogeny, and indirect thermal experience, through parental thermal exposition; see Figure 1). This factor allowed us to compare phenotypic response between P and F1, and also to perform comparisons across P and F1 groups. To compare critical thermal limits among experimental groups, we employed linear mixed model with trial as random effect (random intercept) and sex and treatment as predictor variables. Also, to test the potential of trade‐off between fitness‐related traits ($R_{0}$ *and* $T_{g}$) and *CT_max_* the one‐tailed correlation analysis was conducted.

Because the population density affected significantly $R_{0}$ and $T_{g}$ (Table S1), we assessed the global response of $R_{0}$ *and* $T_{g}$ to temperature and density. We performed a nonparametric regression analysis using a generalized additive model (GAM) incorporating populational density (D), parental thermal environment (*T*_P_), offspring thermal environment (*T*_F1_), and thermal treatment (*treat*) as predictors. We performed GAM since it does not make any a priori assumptions about the shape of relationships between variables, which are key to our evaluation of the effects of population density. Moreover, the main difference between GAMs and linear models is that linear functions of the variables in GAM are replaced by unknown smooth functions, giving additional flexibility to the modeling process (Wood, 2017). The complexity of the curve (the number of degrees of freedom) and the smoothing terms were determined by penalized regression splines and generalized cross‐validation to avoid overfitting (Wood, 2017). Also, we allowed the shrinkage of the smoothers. This technique allows for an extra penalty to be added in the model, and if the penalty is high enough, it will shrink all smoothing coefficients to zero. Model selection was done using the AIC criterion (ΔAIC_c_ < 2; Burnham and Anderson (2002)). To perform pairwise comparisons between experimental groups, we performed a posteriori Tukey test following the linear mixed models or GAMs.

All analyses and visualizations were performed in the R statistical environment (http://www.R-project.org/).

## 3 RESULTS

**FIGURE 2:**
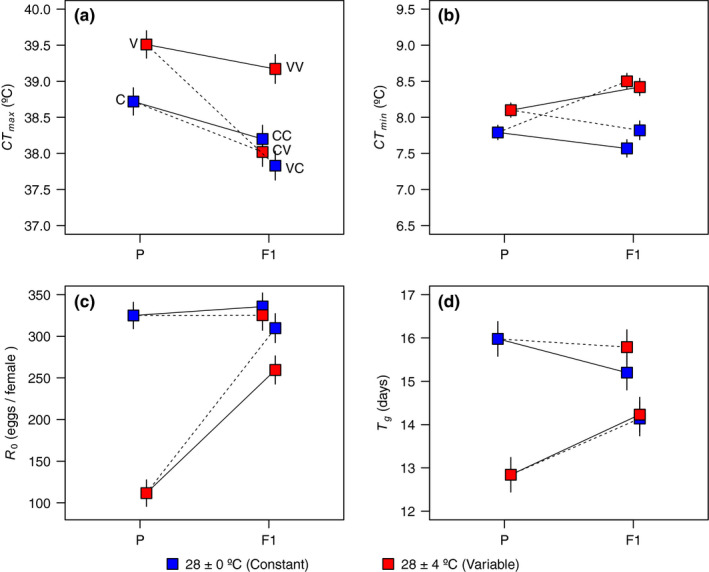
Critical thermal limits (*CT_max_* (a) and *CT_min_* (b)) and demographic parameters ($R_{0}$ (c) and $T_{g}(d$) in our experimental groups. Parental flies (P) and their offspring (F1) were reared in a constant (C, 28 ± 0ºC) or variable (V, 28 ± 4ºC) thermal environment. Solid and dashed lines in the right panels represent similar or alternate thermal environments between P and F1 (see Figure 1). Colored points represent the thermal environment experienced by flies. Values are shown as mean ± *SE*

Maximum and minimum critical thermal limits were differentially affected by the thermal environment experienced by flies. *CT_max_* was significantly higher in flies from parental generation reared at variable thermal environment (*V*) than those reared at constant temperature (C), while *CT_min_* was not different between parental thermal environments (Figure 2a, b and Figure S1). Interestingly, whereas *CT_max_* was significantly higher in females than males, *CT_min_* did not vary between sex (Table 1, Figure S1). With regard to the F1, the thermal environment experienced by parental generation affected the critical thermal limits of their offspring (Table 1, Figure 2). More specifically, F1 flies raised under variable conditions whose parents were also maintained in this environment (i.e., VV) exhibited a significantly higher *CT_max_* than all other F1 groups (including flies from CV; see Figure 2 and Figure S1). Also, *CT_max_* of F1 flies reared under constant environment, whose parents were reared in the alternative treatment (i.e., VC), had significantly lower *CT_max_* than F1 flies from a constant environment whose parents were maintained in a constant environment (CC). Additionally, cold tolerance of F1 flies reared in a variable thermal environment (i.e., VV and CV) was lower than those reared at C (i.e., CC and VC).

**TABLE 1:** Coefficients of the linear mixed model fitted to data for critical thermal maximum and minimum (*CT_max_* and *CT_min_*) in *Drosophila melanogaster*

Effect	Estimate	*SD*	*df*	*T*	*p*
*CT_max_* (ºC)					
Intercept	38.8	0.15	17.1	243	**<.001**
V	0.83	0.12	329	6.81	**<.001**
CC	−0.36	0.13	315	−2.78	**.005**
CV	−0.55	0.13	313	−4.08	**<.001**
VV	0.55	0.14	325	3.81	**<.001**
VC	−0.78	0.13	320	−5,74	**<.001**
Male	−0.36	0.08	315	−4,31	**<.001**
*CT_min_* (ºC)					
Intercept	7.78	0.09	68.0	79.2	**<.001**
V	0.27	0.11	341	2.36	**.02**
CC	−0.24	0.12	258	−1.88	.06
CV	0.68	0.12	197	6.21	**<.001**
VV	0.59	0.13	184	4.39	**<.001**
VC	0.006	0.13	253	0.04	.96
Male	0.08	0.08	123	1.05	.29

Note

Significant differences are indicated in bold (*p* < .05). *N* = sample size. Multiple comparisons in Figure S1.

**FIGURE 3:**
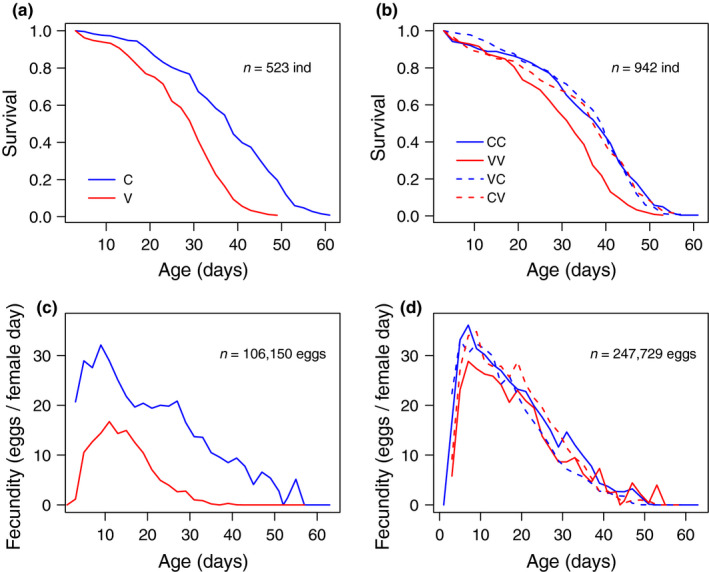
Survival (a and b) and fecundity (c and d) of P and F1 flies of *D. melanogaster* reared in a constant (C, 28 ± 0ºC) or a variable thermal environment (V, 28 ± 4ºC). In colors, thermal treatments for parental generation (C and V, left panels) and their offspring (CC, CV, VC, and VV, right panels). Solid and dashed lines in the right panels represent similar or alternate thermal environments between P and F1 (see Figure 1)

Changing now to demographic descriptors of fitness, both survival and fecundity per female were lower in P flies reared at variable environments (Figure 3, detailed analyses in Table S2), resulting in substantially lower $R_{0}$ and $T_{g}$ in C in comparison with V (Figure 2, Table S3). In contrast, the variation in survival and fecundity was substantially reduced in the F1 regardless of the thermal treatment, which suggests some sort of compensation across generations (Figures 2 and 3). In fact, flies from the second generation exposed to a variable thermal environment showed a significant increase in $R_{0}$ and $T_{g}$ values compared with their parents (Figure 2). Effects of the parental environment were still evident, however, with both $R_{0}$ and $T_{g}$ being on average lower in F1 lines derived from parents subjected to a variable environment (i.e., VC and VV groups), indicating that the apparent compensatory response to a stressful parental environment was only partial (Figure 2). Interestingly, flies whose parents were maintained at a constant temperature (CC and CV) did not show differences in $R_{0}$ and $T_{g}$ between them or their parents regardless of the thermal environment in which they were raised (Figure 2).

Results from our GAM analyses in conjunction with a model comparison approach support the observations listed above (Table 2, Table S3). Thermal treatment had a major effect on these fitness components, explaining up to 53.6% and 34.9% of the variance in $R_{0}$ and $T_{g}$, respectively, after controlling for density (density effects on demographic variables are represented in Figure S2). Furthermore, the models with the lowest AIC in both cases included the treatment factor (see Methods), which encapsulates the thermal environment of the parents (*T*_P_), the offspring (*T*_F1_), and their interaction, and resulted in a model with a better fit than those where these factors were included separately (Table 1).

**TABLE 2:** Results of the GAM fitted for net reproductive rate $R_{0}$ and generation time $T_{g}$ in *Drosophila melanogaster*

Model	Sum edf	logLik	AIC	*r* ^2^
Models for *R*_0_				
*s*(*D*, *by* = *treat*) + *treat*	**5.77**	**−1127**	**2,279**	**53.6**
*s*(*D*, *by* = *T*_P_) + *treat*	3. 53	−1131	2,283	51.5
*s*(*D*, *by* = *T*_F1_ *)* + *treat*	2.55	−1137	2,293	48.2
*s*(*D*) + *treat*	1.41	−1137	2,291	48.2
Models for *T_g_*				
s(*D*, *by* = *treat*) + *treat*	**6.09**	**−427.3**	**881**	**34.9**
s(*D*, *by* = *T*_P_) + *treat*	3.65	−438.6	898	26.5
s(*D*, *by* = *T*_F1_) + *treat*	2.53	−434.2	887	30.0
s(*D*) + *treat*	1.74	−438.2	893	26.8

Note

*D* is population density, *treat* is thermal treatment, *T*_P_ is parental thermal environment, and *T*_F1_ is offspring thermal environment. *s* represents the cubic regression spline for these variables, Sum edf is the sum of effective degrees of freedom, logLik is log‐likelihood values, AIC is Akaike information criterion for the model, and *r* ^2^ is determination coefficient. Models with the best fit are shown in bold.

Interestingly, $R_{0}$ and *CT_max_* were negative and significantly correlated (*r = −0*.*8*,* T = −2*.*69*,* df = 4*,* p = *.*027*), but we did not find significant correlations among $T_{g}$ and *CT_max_* (*r = −0*.*55*,* T = −1*.*32*,* df = 4*,* p = *.*12*).

## 4 DISCUSSION

Phenotypic plasticity involves phenotypic changes associated with environmental conditions and may favor the establishment or persistence of organisms in changing environments (Ghalambor, McKay, Carroll, & Reznick, 2007). Consequently, plasticity may potentially affect the selective pressures that a population encounters and, as a result, its evolutionary trajectory (Bonduriansky, Crean, & Day, 2012; Oster & Alberch, 1982). Our experiment illustrates how within‐generational plasticity and transgenerational effects can ameliorate the impact of stressful thermal conditions on physiological and fitness‐related traits. In this context, our main results can be summarized as follows. First‐generation flies subjected to a variable and stressful thermal environment exhibited higher *CT_max_* when compared against their counterparts maintained at a constant temperature. This plastic and seemingly adaptive response came at costs, however, since these flies also exhibited lower survival rates, fecundity (Figure 3), and ultimately $R_{0}$ (Figure 2). The inverse relationship between $T_{g}$ and temperature in ectotherms, apparently advantageous (Gillooly, 2000; Taylor, 1981), here was accompanied by a substantial drop in life spam, fecundity, and $R_{0}$. Interestingly, these maladaptive plastic responses were less evident in their offspring (Figures 2 and 3), suggesting partial compensation mediated to some degree by transgenerational plasticity.

We aimed to compare the response of the flies under variable and constant thermal environments using both direct measures of fitness and physiological proxies of fitness, such as thermal tolerance, and results were dramatically different (Figure 3). Contrary to results by Nyamukondiwa et al. (Nyamukondiwa et al., 2018) who evaluated the influence of thermal variability on heat tolerance, our results show that *CT_max_* was positively affected by thermal variability, although with adverse effects on fitness.

These results are intriguing because the temperature peak in the variable thermal environment (32ºC) was substantially lower than the estimated *CT_max_* (~39ºC), and yet this temperature was clearly stressful and impacted survival (Figure 3). Although the impact of temperature peak and time of exposure to thermal extremes on organisms cannot be disentangled, the differences in exposure time might explain this counterintuitive result, since the temperature range that organisms can tolerate is associated with the duration of thermal stress (Rezende, Castañeda, & Santos, 2014).

In this sense, it has been described that pattern and duration of exposure to high temperatures may have adverse effects on physiological performance and fitness (i.e., time‐dependent effects) (Kingsolver, Higgins, & Augustine, 2015). The thermal environment experienced by flies might have adverse effects on the subsequent generation (Guillaume et al., 2016). For instance, the reduction in *CT_max_* in F1 flies from CV in comparison with flies from V (38.2º C in CV versus 39.6ºC in V) represents a transgenerational cost of the mean temperature in the parental generation (28ºC and 24ºC, respectively). Consequently, prolonged exposure to high and yet less extreme temperatures elicited an increase in *CT_max_* at a cost in survival and, more importantly, in fecundity rates that are suggestive of a trade‐off since less energy could be allocated to reproduction.

These results agree with Folguera et al. (2009) who reported that high environmental thermal amplitude experienced by terrestrial isopods increased the synthesis of stress‐inducible heat‐shock proteins (HSP), but at a metabolic energy cost with negative effects on longevity and growth rate. Not only is the production of HSP metabolically expensive (e.g., protein biosynthesis represents nearly 30%–50% of total cellular energy consumption, Krebs & Feder, 1997, 1998; Krebs & Loeschcke, 1994), but also they require ATP to maintain the structural integrity of other proteins (Hochachka & Somero, 2002).

In this sense, although plastic responses may mitigate the adverse effects of thermal stress, their compensatory effects might be limited by energetic trade‐offs (Bozinovic et al., 2016; Pigliucci, 2001). Consequently, several studies work with the assumption that higher heat tolerance is a beneficial trait (Cavieres, Bogdanovich, & Bozinovic, 2016; Sørensen, Schou, Kristensen, & Loeschcke, 2016; Salachan & Sørensen, 2017; Salinas Santiago, Irvine, Schertzing, Golden, & Munch, 2019), but here we show that this response was accompanied by a decrease in survival and fecundity, highlighting the importance of incorporating direct measures of fitness in physiological studies in order to have a broad understanding of the implications of phenotypic changes in response to environmental inputs.

Interestingly, fitness cost of living under a variable temperature decreased significantly in the second generation, providing evidence of partial compensation to a stressful thermal environment. This cross‐generational compensatory response involves a 133% increase in $R_{0}$ in the F1 in comparison with P ($R_{0}$ = 259 in VV versus 111 eggs/female in V), but values were still 25% lower than in flies reared at a constant temperature ($R_{0}$ = 325 eggs/female). Also, the drop of 192% in $R_{0}$ in flies from V versus CV ($R_{0}$ = 111 in V versus 325 eggs/female in CV) could be caused by the differences in acclimation temperature in the parental generation (24 versus 28ºC, respectively). Our results agree with previous studies that have documented that parental experience may modify the response to environmental input in their offspring. For instance, rapid compensatory responses in thermal tolerance and/or reproductive output have been described in the marine polychaete *Ophryotrocha labronica* subjected to a low CO_2_ environment (Rodríguez‐Romero, Jarrold, Massamba‐N’Siala, Spicer, & Calosi, 2016), in *Daphnia magna* raised with toxic cyanobacteria (Gustafsson, Rengefors, & Hansson, 2005), or coral reef fish *Acanthochromis polycanthus* who after gradual warming over generations improved the reproductive output (Donelson et al., 2016) (see also Jensen, Allen, & Marshall, 2014; Thor & Dupont, 2015). Overall, these studies suggest that populations can respond rapidly to pronounced environmental changes, providing putative evidence that nongenetic inheritance might underlie observed responses to rapid changes in climatic conditions (Rando & Verstrepen, 2007).

The potential impact of selection should not be dismissed, however. Recovery of reproductive output reported in the literature and in our study might result from the synergistic effects of within‐generation plasticity and genetic adaptation (Rodríguez‐Romero et al., 2016). As has been recently pointed out, estimates of transgenerational plasticity can be biased due to selection and this is the case even in half‐ or full‐sib designs (Santos, Matos, Wang, & Althoff, 2019). Consequently, these effects are particularly relevant in studies dealing with responses to stressful environments employing outbred populations (e.g., this study or results for *O*.* labronica* (Rodríguez‐Romero et al., 2016)), which is a problem often neglected in the literature of transgenerational effects (Burggren, 2014; Ho & Burggren, 2010). For instance, the drop in survival and fecundity in P flies from V may impose strong selection and observed responses in F1 could partly reflect adaptive responses in the Darwinian sense. While the growing evidence indicates that natural populations can respond rapidly to environmental changes, resulting in full or partial compensatory responses to an environmental stress, caution is warranted regarding inferences on the mechanistic basis underlying these responses (Santos et al., 2019).

To summarize, here we describe how an outbred population of *D*.* melanogaster* responds rapidly to changing thermal conditions within and across generations. Our analyses provide evidence of a trade‐off between thermal tolerance and fitness components such as fecundity in parental flies and pronounced albeit incomplete compensatory responses in their offspring. Similar approaches are necessary to extend studies from within‐generational responses to responses across multiple generations. In this context, we urge future research to be tailored to specific climatic scenarios or geographic regions, aiming to build explanations and predict, in the near future, the potential responses of natural populations to ongoing global warming.

## CONFLICT OF INTERESTS

The authors declare no competing interests.

## AUTHOR CONTRIBUTION

**Grisel Cavieres:** Conceptualization (lead); Formal analysis (equal); Funding acquisition (lead); Investigation (equal); Methodology (equal); Project administration (lead); Resources (supporting); Supervision (lead); Visualization (equal); Writing‐original draft (lead); Writing‐review & editing (equal). **Enrico L. Rezende:** Formal analysis (equal); Funding acquisition (supporting); Visualization (equal); Writing‐original draft (supporting); Writing‐review & editing (equal). **Sabrina Clavijo‐Baquet:** Formal analysis (equal); Investigation (equal). **Jose M. Alruiz:** Investigation (equal). **Carla Rivera‐Rebella:** Investigation (equal). **Francisca Boher:** Investigation (equal). **Francisco Bozinovic:** Conceptualization (equal); Funding acquisition (supporting); Resources (lead); Visualization (equal); Writing‐original draft (supporting); Writing‐review & editing (equal).

## Supporting information

Fig S1

Fig S2

Supplementary Material

Supplementary Material

## Data Availability

The data sets analyzed during the current study are available in the Dryad Digital Repository https://doi.org/10.5061/dryad.8931zcrmr.
