# Supplementary figures and images for: Rapid within‐ and transgenerational changes in thermal tolerance and fitness in variable thermal landscapes
Source: Ecol Evol. 2020 Jul 16;10(15):8105–13. doi: 10.1002/ece3.6496 (PMC7417229; doi:10.1002/ece3.6496)

(A)

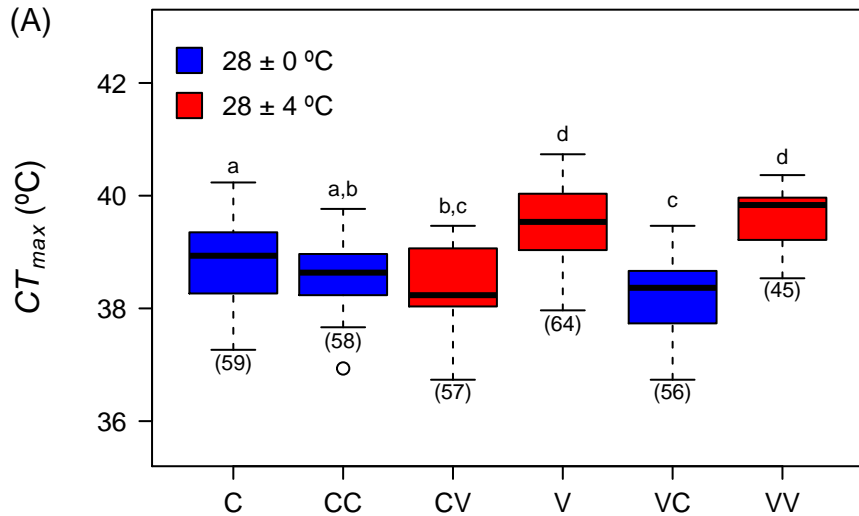

(B)

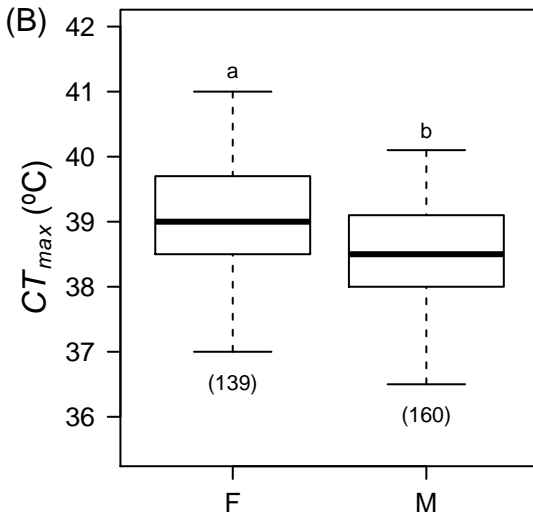

(C)

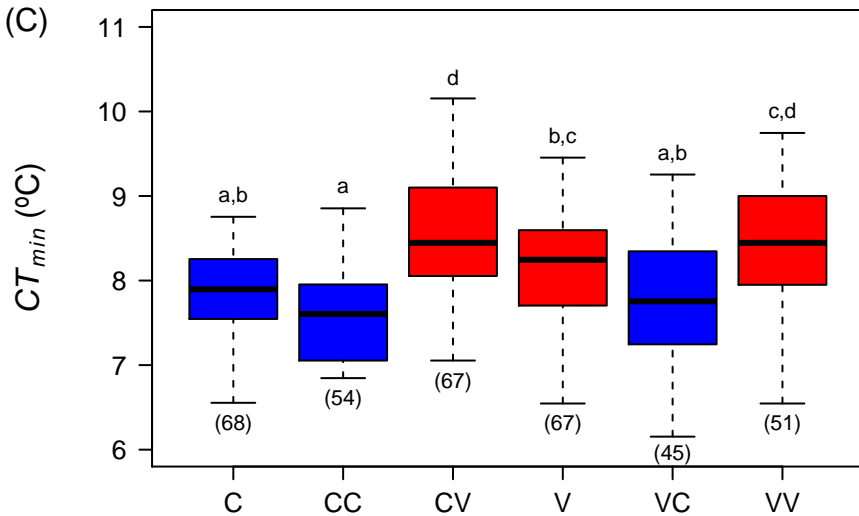

(D)

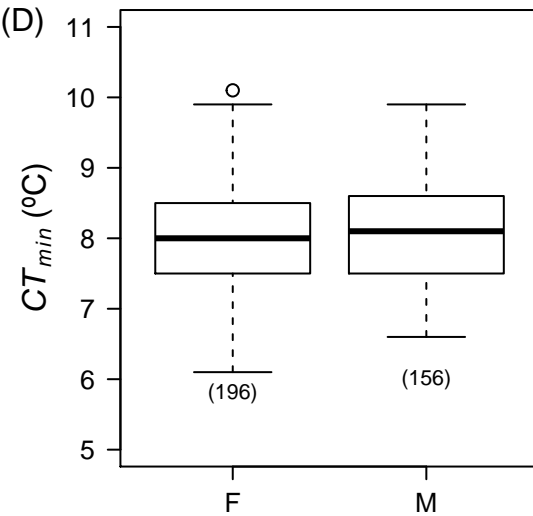

Supplement: Supplementary file 1 — Fig S1 [file ECE3-10-8105-s001.pdf]

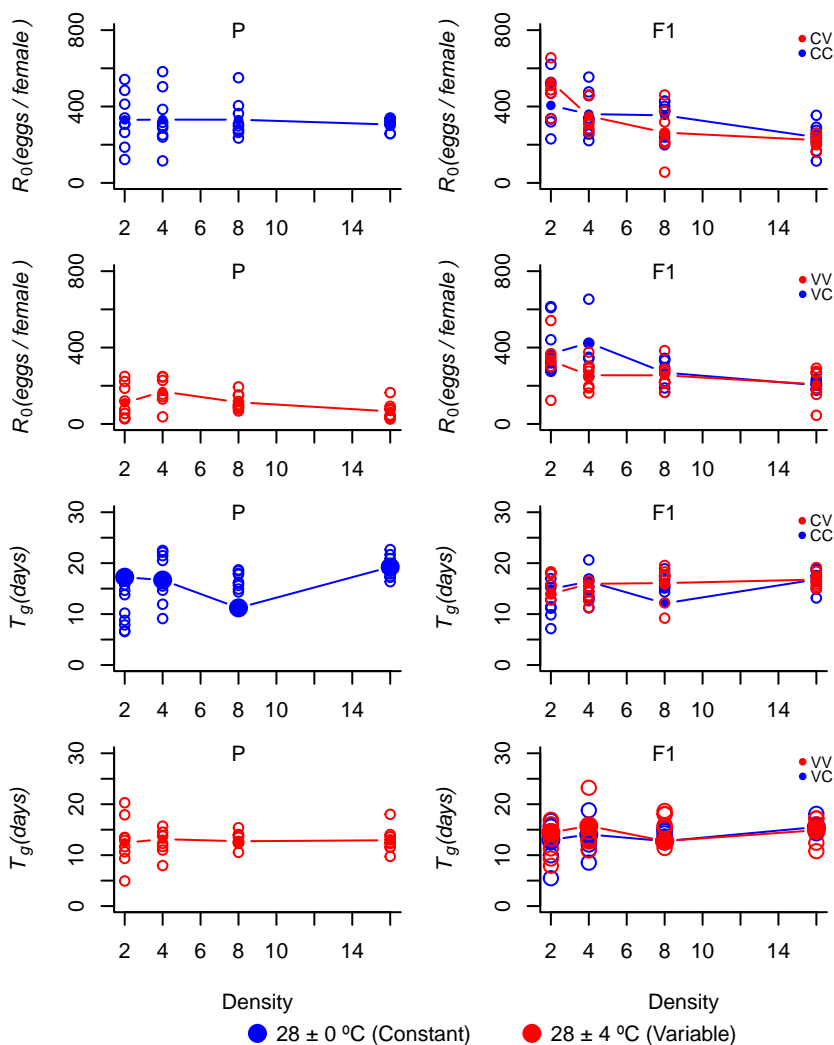

Supplement: Supplementary file 2 — Fig S2 [file ECE3-10-8105-s002.pdf]
