# Supplementary material for: Rapid within‐ and transgenerational changes in thermal tolerance and fitness in variable thermal landscapes
Source: Ecol Evol. 2020 Jul 16;10(15):8105–13. doi: 10.1002/ece3.6496 (PMC7417229; doi:10.1002/ece3.6496)
Supplement: Supplementary file 3 — Supplementary Material [file ECE3-10-8105-s003.docx]

**Supplementary Material**

**Table S1** Analyses of variance (ANOVA) for net reproductive rate (*R*_0_) and generation time (*T_g_*) in *Drosophila melanogaster*. *treat* represent the thermal treatment experience by flies (for details see Fig. 1). *D* represent population densities expressed as individuals per treatment (see methods). Significant P-values are in bold.

| Effect | Df | Sum Sq | Mean Sq | F | P |
| --- | --- | --- | --- | --- | --- |
| ***R_0_ (number of eggs /female)*** | | | | | |
| *treat* | 5 | 1216619 | 243324 | 26.79 | **<0.0001** |
| *D* | 3 | 430271 | 143424 | 15.79 | **<0.0001** |
| *treat* : *D* | 15 | 250688 | 16713 | 1.84 | **0.03** |
| Residuals | 166 | 1507334 | 9080 |  |  |
| ***T_g_ (days)*** | | | | | |
| *treat* | 5 | 216.7 | 43.35 | 6.94 | **<0.0001** |
| *D* | 3 | 229.9 | 76.64 | 12.2 | **<0.0001** |
| *treat* : *D* | 15 | 199.5 | 13.30 | 2.13 | **0.018** |
| Residuals | 162 | 1010.6 | 6.24 |  |  |

**Table S2** Generalized linear models and coefficients comparing how survival and fecundity are affected by the thermal treatment (for details see Fig. 2). For relative survival, we performed a linear regression of survival – 1 through the origin and tested for the interaction *time* : *treat* to compare slopes across treatments. For fecundity, we compared treatments controlling for temporal effects with a regular linear regression starting at day 9, which comprises the temporal window in which fecundity decreased roughly linearly with time (Fig. 2). Significant P-values are in bold.

| Effect | Df | Sum Sq | F | P |
| --- | --- | --- | --- | --- |
| ***Survival*** | | |  | |
| *time : treat* | 6 | 50.568 | 562.59 | **<0.0001** |
| Residuals | 160 | 2.397 |  |  |
|  | |  |  |  |
| *Slope ± SE (Δ survival/day)* | | | | |
| C | -0.0147 | ± 0.0006 |  |  |
| V | -0.0198 | ± 0.0008 |  |  |
| CC | -0.0155 | ± 0.0006 |  |  |
| CV | -0.0156 | ± 0.0006 |  |  |
| VC | -0.0152 | ± 0.0006 |  |  |
| VV | -0.0183 | ± 0.0007 |  |  |
|  |  |  |  |  |
| ***Fecundity (number of eggs/female day)*** | | | | |
| *time* | 1 | 11501 | 1440.40 | **<0.0001** |
| *treat* | 6 | 24985 | 521.54 | **<0.0001** |
| Residuals | 126 | 1006 |  |  |
|  | |  |  |  |
| *Intercept ± SE (eggs/female at day 0)* | | | | |
| C | 37.54 | ± 0.84 |  |  |
| V | 24.09 | ± 0.80 |  |  |
| CC | 36.30 | ± 0.80 |  |  |
| CV | 36.09 | ± 0.83 |  |  |
| VC | 33.86 | ± 0.82 |  |  |
| VV | 34.34 | ± 0.83 |  |  |

**Table S3** Coefficients of the generalized additive model (GAM) to net reproductive rate (*R*_0_) and generation time (*T_g_*) in *Drosophila melanogaster*. Offspring and parental generation were reared in one of two thermal environments, constant (C) or variable (V). Abbreviations represent the thermal treatments for the parental generation (C and V) and the offspring (CC, CV, VV and VC). Significant P-values are in bold.

| Effect | Estimate | SD | t-value | P |
| --- | --- | --- | --- | --- |
| ***R_0_ (number of eggs /female)*** | | | | |
| Intercept (C) | 325 | 15.9 | 20.42 | **< 0.001** |
| V | -213 | 22.5 | -9.47 | **< 0.001** |
| CC | 10.8 | 23.3 | 0.19 | 0.85 |
| CV | 0.38 | 24.2 | -0.16 | 0.98 |
| VV | -65.4 | 23.2 | -2.82 | **0.005** |
| VC | -15.2 | 23.6 | -0.64 | 0.52 |
| ***T_g_ (days)*** |  |  |  |  |
| Intercept (C) | 15.98 | 0.44 | 36.3 | **< 0.001** |
| V | -3.13 | 0.61 | -5.07 | **< 0.001** |
| CC | -0.77 | 0.62 | -1.24 | 0.21 |
| CV | -0.19 | 0.63 | -0.31 | 0.76 |
| VC | -1.84 | 0.63 | -2.91 | **0.004** |
| VV | -1.74 | 0.63 | -2.76 | **0.006** |

**Figure S1** Critical thermal minima and maxima (*CT_min_* and *CT_max_*, respectively) in *D. melanogaster* reared in constant (C, 28 ± 0ºC) or variable (V, 28 ± 4ºC) thermal environments. In colors thermal treatments for parental generation and their offspring, with acronyms shown in Fig. 1. Mean estimates for females (F) and males (M) are shown in the right panels (B,C). The boxplots show the median, 25% and 75% percentiles (hinges) and 95% confidence intervals (notches). Different letters indicate significant differences between values. Numbers in parentheses are sample size.

**Figure S2** Best model for net reproductive rate (*R*_0_) and generation time (*T_g_*) fitted to experimental data. Predicted and observed values for parental of F1 generations. In colors thermal treatments for parental generation (P) and their offspring (F1), with acronyms shown in Fig. 1. Population densities are expressed as individuals per treatment. Left, model considering Parental generation. Right, the same model considering their offspring. Note that several points are overlapped.
