# Supplementary material for: Rapid within‐ and transgenerational changes in thermal tolerance and fitness in variable thermal landscapes
Source: Ecol Evol. 2020 Jul 16;10(15):8105–13. doi: 10.1002/ece3.6496 (PMC7417229; doi:10.1002/ece3.6496)
Supplement: Supplementary file 4 — Supplementary Material [file ECE3-10-8105-s004.pdf]

Suplemmentary material

Life table for *D. melanogaster* reared in a constant (C, 28 ± 0 ºC) or a variable thermal environment (V, 28 ± 4 ºC). Thermal treatments for parental generation (C and V) and their offspring (CC, CV, VC, VV).

Treat: Treatment code
Rep: Replica Number
Density: Individual density
Age: Age or time at measure
Survival:Number of survival individuals of the original cohort
Eggs/per capita: Numbers of eggs per time step divided by number of survival individuals at same time (Fecundity)
Surv %=% of survival individuals
lx*mx= Survival x Fecundity
x*lx*mx= Age x Survival x Fecundity

Treat	Rep	Density	Age	Survival	Eggs/per capita	Surv %	lx*mx	x*lx*mx
C	1	2	3	2	30,5	1	30,5	91,5
C	1	2	5	2	15,5	1	15,5	77,5
C	1	2	7	2	24	1	24	168
C	1	2	9	2	43,5	1	43,5	391,5
C	1	2	11	2	27,5	1	27,5	302,5
C	1	2	13	2	9,5	1	9,5	123,5
C	1	2	15	2	4,5	1	4,5	67,5
C	1	2	17	2	16	1	16	272
C	1	2	19	2	13,5	1	13,5	256,5
C	1	2	21	2	24	1	24	504
C	1	2	23	2	23,5	1	23,5	540,5
C	1	2	25	2	13,5	1	13,5	337,5
C	1	2	27	2	16	1	16	432
C	1	2	29	2	3	1	3	87
C	1	2	31	2	0	1	0	0
C	1	2	33	1	0	0,5	0	0
C	1	2	35	1	0	0,5	0	0
C	1	2	37	0	0	0	0	0
C	2	2	3	2	19	1	19	57
C	2	2	5	2	49	1	49	245
C	2	2	7	2	22	1	22	154
C	2	2	9	2	57	1	57	513
C	2	2	11	2	60	1	60	660
C	2	2	13	2	26	1	26	338
C	2	2	15	2	52,5	1	52,5	787,5
C	2	2	17	2	15,5	1	15,5	263,5
C	2	2	19	2	34	1	34	646
C	2	2	21	2	35	1	35	735
C	2	2	23	2	37	1	37	851

Suplemmentary material

C	2	2	25	2	33,5	1	33,5	837,5
C	2	2	27	2	45,5	1	45,5	1228,5
C	2	2	29	2	27,5	1	27,5	797,5
C	2	2	31	2	19	1	19	589
C	2	2	33	2	4,5	1	4,5	148,5
C	2	2	35	2	4,5	1	4,5	157,5
C	2	2	37	2	0	1	0	0
C	2	2	39	1	0	0,5	0	0
C	2	2	41	1	0	0,5	0	0
C	2	2	43	1	0	0,5	0	0
C	2	2	45	1	0	0,5	0	0
C	2	2	47	1	0	0,5	0	0
C	2	2	49	0	0	0	0	0
C	3	2	3	2	71,5	1	71,5	214,5
C	3	2	5	2	42	1	42	210
C	3	2	7	2	34,5	1	34,5	241,5
C	3	2	9	1	12	0,5	6	54
C	3	2	11	1	9	0,5	4,5	49,5
C	3	2	13	1	15	0,5	7,5	97,5
C	3	2	15	1	10	0,5	5	75
C	3	2	17	1	13	0,5	6,5	110,5
C	3	2	19	1	3	0,5	1,5	28,5
C	3	2	21	1	11	0,5	5,5	115,5
C	3	2	23	1	5	0,5	2,5	57,5
C	3	2	25	1	0	0,5	0	0
C	3	2	27	1	0	0,5	0	0
C	3	2	29	1	0	0,5	0	0
C	3	2	31	1	0	0,5	0	0
C	3	2	33	1	0	0,5	0	0
C	3	2	35	1	0	0,5	0	0
C	3	2	37	1	0	0,5	0	0
C	3	2	39	1	0	0,5	0	0
C	3	2	41	1	0	0,5	0	0
C	3	2	43	1	0	0,5	0	0
C	3	2	45	1	0	0,5	0	0
C	3	2	47	1	0	0,5	0	0
C	3	2	49	1	0	0,5	0	0
C	3	2	51	1	0	0,5	0	0
C	3	2	53	0	0	0	0	0
C	4	2	3	2	39,5	1	39,5	118,5
C	4	2	5	2	58	1	58	290
C	4	2	7	2	49	1	49	343
C	4	2	9	2	54,5	1	54,5	490,5
C	4	2	11	2	70,5	1	70,5	775,5
C	4	2	13	2	38	1	38	494

Suplemmentary material

C	4	2	15	2	27,5	1	27,5	412,5
C	4	2	17	2	3,5	1	3,5	59,5
C	4	2	19	2	0	1	0	0
C	4	2	21	2	0	1	0	0
C	4	2	23	2	0	1	0	0
C	4	2	25	1	0	0,5	0	0
C	4	2	27	1	0	0,5	0	0
C	4	2	29	1	0	0,5	0	0
C	4	2	31	1	0	0,5	0	0
C	4	2	33	1	0	0,5	0	0
C	4	2	35	1	0	0,5	0	0
C	4	2	37	1	0	0,5	0	0
C	4	2	39	1	0	0,5	0	0
C	4	2	41	1	0	0,5	0	0
C	4	2	43	1	0	0,5	0	0
C	4	2	45	1	0	0,5	0	0
C	4	2	47	1	0	0,5	0	0
C	4	2	49	1	0	0,5	0	0
C	4	2	51	1	0	0,5	0	0
C	4	2	53	1	0	0,5	0	0
C	4	2	55	1	0	0,5	0	0
C	4	2	57	1	0	0,5	0	0
C	4	2	59	1	0	0,5	0	0
C	4	2	61	1	0	0,5	0	0
C	4	2	63	0	0	0	0	0
C	5	2	3	2	23,5	1	23,5	70,5
C	5	2	5	2	55,5	1	55,5	277,5
C	5	2	7	2	40,5	1	40,5	283,5
C	5	2	9	2	38	1	38	342
C	5	2	11	2	65	1	65	715
C	5	2	13	2	40,5	1	40,5	526,5
C	5	2	15	2	45	1	45	675
C	5	2	17	2	30	1	30	510
C	5	2	19	2	24	1	24	456
C	5	2	21	2	23,5	1	23,5	493,5
C	5	2	23	2	15,5	1	15,5	356,5
C	5	2	25	2	24	1	24	600
C	5	2	27	2	19	1	19	513
C	5	2	29	2	12	1	12	348
C	5	2	31	2	11	1	11	341
C	5	2	33	2	9,5	1	9,5	313,5
C	5	2	35	2	7	1	7	245
C	5	2	37	2	0	1	0	0
C	5	2	39	1	2	0,5	1	39
C	5	2	41	1	0	0,5	0	0

Suplemmentary material

C	5	2	43	1	0	0,5	0	0
C	5	2	45	0	0	0	0	0
C	6	2	3	2	29,5	1	29,5	88,5
C	6	2	5	2	50	1	50	250
C	6	2	7	2	33,5	1	33,5	234,5
C	6	2	9	2	43	1	43	387
C	6	2	11	2	47,5	1	47,5	522,5
C	6	2	13	2	24	1	24	312
C	6	2	15	2	28,5	1	28,5	427,5
C	6	2	17	2	38	1	38	646
C	6	2	19	2	14,5	1	14,5	275,5
C	6	2	21	2	0	1	0	0
C	6	2	23	2	0	1	0	0
C	6	2	25	1	0	0,5	0	0
C	6	2	27	1	0	0,5	0	0
C	6	2	29	1	0	0,5	0	0
C	6	2	31	1	0	0,5	0	0
C	6	2	33	1	0	0,5	0	0
C	6	2	35	1	0	0,5	0	0
C	6	2	37	1	0	0,5	0	0
C	6	2	39	1	0	0,5	0	0
C	6	2	41	1	0	0,5	0	0
C	6	2	43	0	0	0	0	0
C	8	2	3	2	43	1	43	129
C	8	2	5	2	54	1	54	270
C	8	2	7	2	68,5	1	68,5	479,5
C	8	2	9	2	48	1	48	432
C	8	2	11	2	63	1	63	693
C	8	2	13	2	24	1	24	312
C	8	2	15	2	7	1	7	105
C	8	2	17	2	0	1	0	0
C	8	2	19	1	0	0,5	0	0
C	8	2	21	1	0	0,5	0	0
C	8	2	23	0	0	0	0	0
C	9	2	3	2	18	1	18	54
C	9	2	5	1	54	0,5	27	135
C	9	2	7	1	84	0,5	42	294
C	9	2	9	1	70	0,5	35	315
C	9	2	11	1	0	0,5	0	0
C	9	2	13	1	1	0,5	0,5	6,5
C	9	2	15	1	0	0,5	0	0
C	9	2	17	0	0	0	0	0
C	10	2	3	2	2	1	2	6
C	10	2	5	2	49,5	1	49,5	247,5
C	10	2	7	2	50	1	50	350

Suplemmentary material

C	10	2	9	2	55	1	55	495
C	10	2	11	2	47	1	47	517
C	10	2	13	2	37,5	1	37,5	487,5
C	10	2	15	2	15	1	15	225
C	10	2	17	2	12,5	1	12,5	212,5
C	10	2	19	2	9,5	1	9,5	180,5
C	10	2	21	2	9	1	9	189
C	10	2	23	2	7	1	7	161
C	10	2	25	2	18,5	1	18,5	462,5
C	10	2	27	2	35,5	1	35,5	958,5
C	10	2	29	2	25,5	1	25,5	739,5
C	10	2	31	2	18	1	18	558
C	10	2	33	2	13,5	1	13,5	445,5
C	10	2	35	1	12	0,5	6	210
C	10	2	37	1	0	0,5	0	0
C	10	2	39	1	2	0,5	1	39
C	10	2	41	1	1	0,5	0,5	20,5
C	10	2	43	1	0	0,5	0	0
C	10	2	45	0	0	0	0	0
C	1	4	3	4	13,5	1	13,5	40,5
C	1	4	5	4	16,25	1	16,25	81,25
C	1	4	7	4	16	1	16	112
C	1	4	9	4	26,75	1	26,75	240,75
C	1	4	11	4	11,25	1	11,25	123,75
C	1	4	13	4	15,75	1	15,75	204,75
C	1	4	15	4	10	1	10	150
C	1	4	17	4	5,75	1	5,75	97,75
C	1	4	19	4	0	1	0	0
C	1	4	21	4	0	1	0	0
C	1	4	23	3	0	0,75	0	0
C	1	4	25	3	0	0,75	0	0
C	1	4	27	3	0	0,75	0	0
C	1	4	29	3	0	0,75	0	0
C	1	4	31	2	0	0,5	0	0
C	3	4	3	4	12,5	1	12,5	37,5
C	3	4	5	4	26,25	1	26,25	131,25
C	3	4	7	4	31,25	1	31,25	218,75
C	3	4	9	4	28	1	28	252
C	3	4	11	4	29,5	1	29,5	324,5
C	3	4	13	4	24,5	1	24,5	318,5
C	3	4	15	4	24	1	24	360
C	3	4	17	4	24,75	1	24,75	420,75
C	3	4	19	4	24,25	1	24,25	460,75
C	3	4	21	4	6,5	1	6,5	136,5
C	3	4	23	4	4	1	4	92

Suplemmentary material

C	3	4	25	3	6	0,75	4,5	112,5
C	3	4	27	3	0	0,75	0	0
C	3	4	29	2	0	0,5	0	0
C	3	4	31	2	0	0,5	0	0
C	3	4	33	0	0	0	0	0
C	4	4	3	4	22,75	1	22,75	68,25
C	4	4	5	4	32	1	32	160
C	4	4	7	4	24	1	24	168
C	4	4	9	4	33,75	1	33,75	303,75
C	4	4	11	4	39,75	1	39,75	437,25
C	4	4	13	4	36,75	1	36,75	477,75
C	4	4	15	4	39	1	39	585
C	4	4	17	4	32,25	1	32,25	548,25
C	4	4	19	4	26,25	1	26,25	498,75
C	4	4	21	4	24,5	1	24,5	514,5
C	4	4	23	3	22,33333333	0,75	16,75	385,25
C	4	4	25	3	21,66666667	0,75	16,25	406,25
C	4	4	27	2	58,5	0,5	29,25	789,75
C	4	4	29	2	23,5	0,5	11,75	340,75
C	4	4	31	1	60	0,25	15	465
C	4	4	33	1	56	0,25	14	462
C	4	4	35	1	50	0,25	12,5	437,5
C	4	4	37	1	48	0,25	12	444
C	4	4	39	1	45	0,25	11,25	438,75
C	4	4	41	1	51	0,25	12,75	522,75
C	4	4	43	1	58	0,25	14,5	623,5
C	4	4	45	1	28	0,25	7	315
C	4	4	47	1	37	0,25	9,25	434,75
C	4	4	49	1	36	0,25	9	441
C	4	4	51	1	5	0,25	1,25	63,75
C	4	4	53	0	0	0	0	0
C	5	4	3	4	27,75	1	27,75	83,25
C	5	4	5	4	32,25	1	32,25	161,25
C	5	4	7	4	27	1	27	189
C	5	4	9	4	28,25	1	28,25	254,25
C	5	4	11	4	32,5	1	32,5	357,5
C	5	4	13	4	47,75	1	47,75	620,75
C	5	4	15	4	35	1	35	525
C	5	4	17	4	37,25	1	37,25	633,25
C	5	4	19	4	36,75	1	36,75	698,25
C	5	4	21	4	14	1	14	294
C	5	4	23	3	19	0,75	14,25	327,75
C	5	4	25	3	18,66666667	0,75	14	350
C	5	4	27	3	22,66666667	0,75	17	459
C	5	4	29	3	9,666666667	0,75	7,25	210,25

Suplemmentary material

C	5	4	31	3	4	0,75	3	93
C	5	4	33	2	8,5	0,5	4,25	140,25
C	5	4	35	2	1	0,5	0,5	17,5
C	5	4	37	1	14	0,25	3,5	129,5
C	5	4	39	1	4	0,25	1	39
C	5	4	41	1	7	0,25	1,75	71,75
C	5	4	43	1	0	0,25	0	0
C	5	4	45	1	0	0,25	0	0
C	5	4	47	1	0	0,25	0	0
C	5	4	49	1	0	0,25	0	0
C	5	4	51	1	0	0,25	0	0
C	5	4	53	0	0	0	0	0
C	6	4	3	4	22,5	1	22,5	67,5
C	6	4	5	4	13,75	1	13,75	68,75
C	6	4	7	4	8,25	1	8,25	57,75
C	6	4	9	4	38	1	38	342
C	6	4	11	4	23,25	1	23,25	255,75
C	6	4	13	4	21,5	1	21,5	279,5
C	6	4	15	4	6,5	1	6,5	97,5
C	6	4	17	4	26,5	1	26,5	450,5
C	6	4	19	4	0,5	1	0,5	9,5
C	6	4	21	4	15,5	1	15,5	325,5
C	6	4	23	4	19	1	19	437
C	6	4	25	4	10,25	1	10,25	256,25
C	6	4	27	4	3	1	3	81
C	6	4	29	4	7	1	7	203
C	6	4	31	4	12,25	1	12,25	379,75
C	6	4	33	4	12,5	1	12,5	412,5
C	6	4	35	3	11,33333333	0,75	8,5	297,5
C	6	4	37	3	24,66666667	0,75	18,5	684,5
C	6	4	39	2	0,5	0,5	0,25	9,75
C	6	4	41	2	30	0,5	15	615
C	6	4	43	2	10,5	0,5	5,25	225,75
C	6	4	45	1	26	0,25	6,5	292,5
C	6	4	47	1	26	0,25	6,5	305,5
C	6	4	49	1	11	0,25	2,75	134,75
C	6	4	51	1	6	0,25	1,5	76,5
C	6	4	53	1	19	0,25	4,75	251,75
C	6	4	55	1	30	0,25	7,5	412,5
C	6	4	57	1	0	0,25	0	0
C	6	4	59	0	0	0	0	0
C	7	4	3	4	22	1	22	66
C	7	4	5	4	13,5	1	13,5	67,5
C	7	4	7	4	6,5	1	6,5	45,5
C	7	4	9	4	13,5	1	13,5	121,5

Suplemmentary material

C	7	4	11	4	6,25	1	6,25	68,75
C	7	4	13	4	18,5	1	18,5	240,5
C	7	4	15	4	19,25	1	19,25	288,75
C	7	4	17	4	19,75	1	19,75	335,75
C	7	4	19	4	22,5	1	22,5	427,5
C	7	4	21	2	32	0,5	16	336
C	7	4	23	2	31,5	0,5	15,75	362,25
C	7	4	25	2	29,5	0,5	14,75	368,75
C	7	4	27	2	16,5	0,5	8,25	222,75
C	7	4	29	2	12	0,5	6	174
C	7	4	31	2	6	0,5	3	93
C	7	4	33	2	23,5	0,5	11,75	387,75
C	7	4	35	2	29	0,5	14,5	507,5
C	7	4	37	2	27	0,5	13,5	499,5
C	7	4	39	2	20,5	0,5	10,25	399,75
C	7	4	41	1	35	0,25	8,75	358,75
C	7	4	43	1	37	0,25	9,25	397,75
C	7	4	45	1	0	0,25	0	0
C	7	4	47	1	27	0,25	6,75	317,25
C	7	4	49	1	19	0,25	4,75	232,75
C	7	4	51	1	13	0,25	3,25	165,75
C	7	4	53	1	0	0,25	0	0
C	7	4	55	1	0	0,25	0	0
C	7	4	57	1	0	0,25	0	0
C	7	4	58	0	0	0	0	0
C	8	4	3	4	10	1	10	30
C	8	4	5	4	25	1	25	125
C	8	4	7	4	13	1	13	91
C	8	4	9	4	33,75	1	33,75	303,75
C	8	4	11	4	31,25	1	31,25	343,75
C	8	4	13	4	26	1	26	338
C	8	4	15	4	22	1	22	330
C	8	4	17	4	15,25	1	15,25	259,25
C	8	4	19	2	37,5	0,5	18,75	356,25
C	8	4	21	2	32,5	0,5	16,25	341,25
C	8	4	23	2	25,5	0,5	12,75	293,25
C	8	4	25	2	32,5	0,5	16,25	406,25
C	8	4	27	2	14,5	0,5	7,25	195,75
C	8	4	29	2	31,5	0,5	15,75	456,75
C	8	4	31	2	30	0,5	15	465
C	8	4	33	1	33	0,25	8,25	272,25
C	8	4	35	1	5	0,25	1,25	43,75
C	8	4	37	1	7	0,25	1,75	64,75
C	8	4	39	1	5	0,25	1,25	48,75
C	8	4	41	1	8	0,25	2	82

Suplemmentary material

C	8	4	43	1	9	0,25	2,25	96,75
C	8	4	45	1	7	0,25	1,75	78,75
C	8	4	47	1	8	0,25	2	94
C	8	4	49	1	6	0,25	1,5	73,5
C	8	4	51	0	0	0	0	0
C	9	4	3	4	13	1	13	39
C	9	4	5	4	21,25	1	21,25	106,25
C	9	4	7	4	26,25	1	26,25	183,75
C	9	4	9	4	35	1	35	315
C	9	4	11	4	31,75	1	31,75	349,25
C	9	4	13	4	30	1	30	390
C	9	4	15	4	36,75	1	36,75	551,25
C	9	4	17	4	43,75	1	43,75	743,75
C	9	4	19	4	58,25	1	58,25	1106,75
C	9	4	21	4	48,75	1	48,75	1023,75
C	9	4	23	4	42,25	1	42,25	971,75
C	9	4	25	4	41,5	1	41,5	1037,5
C	9	4	27	4	18,5	1	18,5	499,5
C	9	4	29	3	32,66666667	0,75	24,5	710,5
C	9	4	31	3	12	0,75	9	279
C	9	4	33	3	16,66666667	0,75	12,5	412,5
C	9	4	35	2	19	0,5	9,5	332,5
C	9	4	37	2	25	0,5	12,5	462,5
C	9	4	39	2	27	0,5	13,5	526,5
C	9	4	41	2	15	0,5	7,5	307,5
C	9	4	43	2	32	0,5	16	688
C	9	4	45	2	11,5	0,5	5,75	258,75
C	9	4	47	2	12	0,5	6	282
C	9	4	49	1	51	0,25	12,75	624,75
C	9	4	51	1	24	0,25	6	306
C	9	4	53	0	0	0	0	0
C	10	4	3	4	3,25	1	3,25	9,75
C	10	4	5	4	24	1	24	120
C	10	4	7	4	26,75	1	26,75	187,25
C	10	4	9	4	16,75	1	16,75	150,75
C	10	4	11	4	22,25	1	22,25	244,75
C	10	4	13	4	31,25	1	31,25	406,25
C	10	4	15	4	9,5	1	9,5	142,5
C	10	4	17	4	20,5	1	20,5	348,5
C	10	4	19	4	24,75	1	24,75	470,25
C	10	4	21	4	15,5	1	15,5	325,5
C	10	4	23	4	14,75	1	14,75	339,25
C	10	4	25	3	20	0,75	15	375
C	10	4	27	2	17,5	0,5	8,75	236,25
C	10	4	29	2	28,5	0,5	14,25	413,25

Suplemmentary material

C	10	4	31	2	3	0,5	1,5	46,5
C	10	4	33	2	1,5	0,5	0,75	24,75
C	10	4	35	2	3	0,5	1,5	52,5
C	10	4	37	2	0	0,5	0	0
C	10	4	39	1	0	0,25	0	0
C	10	4	41	0	0	0	0	0
C	1	8	3	8	20,625	1	20,625	61,875
C	1	8	5	8	40,625	1	40,625	203,125
C	1	8	7	8	32,375	1	32,375	226,625
C	1	8	9	8	37	1	37	333
C	1	8	11	7	42,42857143	0,875	37,125	408,375
C	1	8	13	6	34,5	0,75	25,875	336,375
C	1	8	15	6	29	0,75	21,75	326,25
C	1	8	17	6	37,16666667	0,75	27,875	473,875
C	1	8	19	6	27	0,75	20,25	384,75
C	1	8	21	6	22,66666667	0,75	17	357
C	1	8	23	6	10,66666667	0,75	8	184
C	1	8	25	4	24	0,5	12	300
C	1	8	27	4	16,5	0,5	8,25	222,75
C	1	8	29	4	25,5	0,5	12,75	369,75
C	1	8	31	4	18,5	0,5	9,25	286,75
C	1	8	33	4	18	0,5	9	297
C	1	8	35	4	8,75	0,5	4,375	153,125
C	1	8	37	3	18,66666667	0,375	7	259
C	1	8	39	3	18	0,375	6,75	263,25
C	1	8	41	3	7	0,375	2,625	107,625
C	1	8	43	3	4	0,375	1,5	64,5
C	1	8	45	3	3	0,375	1,125	50,625
C	1	8	47	0	0	0	0	0
C	2	8	3	8	19,875	1	19,875	59,625
C	2	8	5	8	22,375	1	22,375	111,875
C	2	8	7	8	14,75	1	14,75	103,25
C	2	8	9	8	19,875	1	19,875	178,875
C	2	8	11	8	19,75	1	19,75	217,25
C	2	8	13	8	34,375	1	34,375	446,875
C	2	8	15	8	14,625	1	14,625	219,375
C	2	8	17	8	11,625	1	11,625	197,625
C	2	8	19	8	30,125	1	30,125	572,375
C	2	8	21	7	32,42857143	0,875	28,375	595,875
C	2	8	23	7	17,28571429	0,875	15,125	347,875
C	2	8	25	6	35,83333333	0,75	26,875	671,875
C	2	8	27	6	18,16666667	0,75	13,625	367,875
C	2	8	29	4	10	0,5	5	145
C	2	8	31	3	16,33333333	0,375	6,125	189,875
C	2	8	33	3	5,666666667	0,375	2,125	70,125

Suplemmentary material

C	2	8	35	3	16,66666667	0,375	6,25	218,75
C	2	8	37	3	13,33333333	0,375	5	185
C	2	8	39	3	9	0,375	3,375	131,625
C	2	8	41	1	24	0,125	3	123
C	2	8	43	1	18	0,125	2,25	96,75
C	2	8	45	1	12	0,125	1,5	67,5
C	2	8	47	1	35	0,125	4,375	205,625
C	2	8	49	0	0	0	0	0
C	3	8	3	8	20,625	1	20,625	61,875
C	3	8	5	8	17,375	1	17,375	86,875
C	3	8	7	8	13,5	1	13,5	94,5
C	3	8	9	8	14,375	1	14,375	129,375
C	3	8	11	8	23,75	1	23,75	261,25
C	3	8	13	8	22,25	1	22,25	289,25
C	3	8	15	8	19,625	1	19,625	294,375
C	3	8	17	8	21,75	1	21,75	369,75
C	3	8	19	8	16,5	1	16,5	313,5
C	3	8	21	8	17,125	1	17,125	359,625
C	3	8	23	8	10,375	1	10,375	238,625
C	3	8	25	8	15,625	1	15,625	390,625
C	3	8	27	8	11,875	1	11,875	320,625
C	3	8	29	8	9,5	1	9,5	275,5
C	3	8	31	8	0,5	1	0,5	15,5
C	3	8	33	8	0	1	0	0
C	3	8	35	8	0	1	0	0
C	3	8	37	5	0	0,625	0	0
C	3	8	39	5	0	0,625	0	0
C	3	8	41	4	0	0,5	0	0
C	3	8	43	3	0	0,375	0	0
C	3	8	45	3	0	0,375	0	0
C	3	8	47	3	0	0,375	0	0
C	3	8	49	3	0	0,375	0	0
C	3	8	51	1	0	0,125	0	0
C	3	8	53	1	0	0,125	0	0
C	3	8	55	1	0	0,125	0	0
C	3	8	57	1	0	0,125	0	0
C	3	8	59	1	0	0,125	0	0
C	3	8	61	1	0	0,125	0	0
C	3	8	63	0	0	0	0	0
C	4	8	3	8	27,25	1	27,25	81,75
C	4	8	5	8	28	1	28	140
C	4	8	7	8	24,5	1	24,5	171,5
C	4	8	9	8	18,375	1	18,375	165,375
C	4	8	11	8	20,625	1	20,625	226,875
C	4	8	13	7	19,57142857	0,875	17,125	222,625

Suplemmentary material

C	4	8	15	7	29,28571429	0,875	25,625	384,375
C	4	8	17	7	17,85714286	0,875	15,625	265,625
C	4	8	19	7	22,14285714	0,875	19,375	368,125
C	4	8	21	5	35,6	0,625	22,25	467,25
C	4	8	23	5	39,2	0,625	24,5	563,5
C	4	8	25	5	32,4	0,625	20,25	506,25
C	4	8	27	4	22,5	0,5	11,25	303,75
C	4	8	29	4	10,5	0,5	5,25	152,25
C	4	8	31	4	0	0,5	0	0
C	4	8	33	4	0	0,5	0	0
C	4	8	35	4	0	0,5	0	0
C	4	8	37	4	0	0,5	0	0
C	4	8	39	4	0	0,5	0	0
C	4	8	41	2	0	0,25	0	0
C	4	8	43	1	0	0,125	0	0
C	4	8	45	1	0	0,125	0	0
C	4	8	47	1	0	0,125	0	0
C	4	8	49	1	0	0,125	0	0
C	4	8	51	1	0	0,125	0	0
C	4	8	53	0	0	0	0	0
C	5	8	3	8	29,5	1	29,5	88,5
C	5	8	5	8	22,75	1	22,75	113,75
C	5	8	7	8	20,125	1	20,125	140,875
C	5	8	9	8	31,5	1	31,5	283,5
C	5	8	11	8	19,125	1	19,125	210,375
C	5	8	13	7	14,28571429	0,875	12,5	162,5
C	5	8	15	5	18,6	0,625	11,625	174,375
C	5	8	17	5	18,2	0,625	11,375	193,375
C	5	8	19	5	18,2	0,625	11,375	216,125
C	5	8	21	3	18,33333333	0,375	6,875	144,375
C	5	8	23	3	39,33333333	0,375	14,75	339,25
C	5	8	25	3	38	0,375	14,25	356,25
C	5	8	27	3	103,3333333	0,375	38,75	1046,25
C	5	8	29	3	27	0,375	10,125	293,625
C	5	8	31	3	18	0,375	6,75	209,25
C	5	8	33	3	13,66666667	0,375	5,125	169,125
C	5	8	35	3	10,66666667	0,375	4	140
C	5	8	37	3	7	0,375	2,625	97,125
C	5	8	39	3	1,666666667	0,375	0,625	24,375
C	5	8	41	2	0	0,25	0	0
C	5	8	43	2	0	0,25	0	0
C	5	8	45	2	0	0,25	0	0
C	5	8	47	1	0	0,125	0	0
C	5	8	49	1	0	0,125	0	0
C	5	8	51	0	0	0	0	0

Suplemmentary material

C	6	8	3	8	37,25	1	37,25	111,75
C	6	8	5	8	41,125	1	41,125	205,625
C	6	8	7	8	34,5	1	34,5	241,5
C	6	8	9	8	35,875	1	35,875	322,875
C	6	8	11	8	24,5	1	24,5	269,5
C	6	8	13	8	30	1	30	390
C	6	8	15	8	47,25	1	47,25	708,75
C	6	8	17	8	29,5	1	29,5	501,5
C	6	8	19	7	41	0,875	35,875	681,625
C	6	8	21	6	36,66666667	0,75	27,5	577,5
C	6	8	23	6	27,16666667	0,75	20,375	468,625
C	6	8	25	6	24,5	0,75	18,375	459,375
C	6	8	27	6	29,66666667	0,75	22,25	600,75
C	6	8	29	6	51,66666667	0,75	38,75	1123,75
C	6	8	31	6	41,83333333	0,75	31,375	972,625
C	6	8	33	6	45,5	0,75	34,125	1126,125
C	6	8	35	6	31,5	0,75	23,625	826,875
C	6	8	37	6	14,83333333	0,75	11,125	411,625
C	6	8	39	6	6,5	0,75	4,875	190,125
C	6	8	41	6	3,333333333	0,75	2,5	102,5
C	6	8	43	5	0	0,625	0	0
C	6	8	45	3	0	0,375	0	0
C	6	8	47	3	0	0,375	0	0
C	6	8	49	1	0	0,125	0	0
C	6	8	51	1	0	0,125	0	0
C	6	8	53	0	0	0	0	0
C	7	8	3	8	21,25	1	21,25	63,75
C	7	8	5	8	35,5	1	35,5	177,5
C	7	8	7	8	31	1	31	217
C	7	8	9	8	22,375	1	22,375	201,375
C	7	8	11	8	19,625	1	19,625	215,875
C	7	8	13	8	20,5	1	20,5	266,5
C	7	8	15	8	17	1	17	255
C	7	8	17	8	14,75	1	14,75	250,75
C	7	8	19	8	13,375	1	13,375	254,125
C	7	8	21	8	3,875	1	3,875	81,375
C	7	8	23	7	21,14285714	0,875	18,5	425,5
C	7	8	25	7	26,14285714	0,875	22,875	571,875
C	7	8	27	7	8,714285714	0,875	7,625	205,875
C	7	8	29	7	16,42857143	0,875	14,375	416,875
C	7	8	31	6	10,83333333	0,75	8,125	251,875
C	7	8	33	5	14,8	0,625	9,25	305,25
C	7	8	35	5	12	0,625	7,5	262,5
C	7	8	37	5	8,8	0,625	5,5	203,5
C	7	8	39	4	3,75	0,5	1,875	73,125

Suplemmentary material

C	7	8	41	4	2	0,5	1	41
C	7	8	43	4	3,25	0,5	1,625	69,875
C	7	8	45	3	1,666666667	0,375	0,625	28,125
C	7	8	47	3	0	0,375	0	0
C	7	8	49	2	0	0,25	0	0
C	7	8	51	2	0	0,25	0	0
C	7	8	53	0	0	0	0	0
C	8	8	3	8	10,875	1	10,875	32,625
C	8	8	5	8	19,375	1	19,375	96,875
C	8	8	7	8	11,125	1	11,125	77,875
C	8	8	9	8	17,875	1	17,875	160,875
C	8	8	11	8	19,75	1	19,75	217,25
C	8	8	13	8	22,25	1	22,25	289,25
C	8	8	15	8	20,375	1	20,375	305,625
C	8	8	17	8	17,75	1	17,75	301,75
C	8	8	19	8	16,75	1	16,75	318,25
C	8	8	21	8	12,25	1	12,25	257,25
C	8	8	23	8	18	1	18	414
C	8	8	25	8	6,25	1	6,25	156,25
C	8	8	27	8	10,375	1	10,375	280,125
C	8	8	29	8	23,5	1	23,5	681,5
C	8	8	31	7	8,285714286	0,875	7,25	224,75
C	8	8	33	7	12,28571429	0,875	10,75	354,75
C	8	8	35	7	10,85714286	0,875	9,5	332,5
C	8	8	37	7	4,857142857	0,875	4,25	157,25
C	8	8	39	5	9,4	0,625	5,875	229,125
C	8	8	41	5	0	0,625	0	0
C	8	8	43	5	0	0,625	0	0
C	8	8	45	4	0	0,5	0	0
C	8	8	47	4	0	0,5	0	0
C	8	8	49	3	0	0,375	0	0
C	8	8	51	3	0	0,375	0	0
C	8	8	53	0	0	0	0	0
C	9	8	3	8	26,25	1	26,25	78,75
C	9	8	5	8	26,75	1	26,75	133,75
C	9	8	7	6	41,83333333	0,75	31,375	219,625
C	9	8	9	6	39,83333333	0,75	29,875	268,875
C	9	8	11	6	27,83333333	0,75	20,875	229,625
C	9	8	13	6	30,66666667	0,75	23	299
C	9	8	15	6	33	0,75	24,75	371,25
C	9	8	17	6	34,33333333	0,75	25,75	437,75
C	9	8	19	6	30,5	0,75	22,875	434,625
C	9	8	21	6	35	0,75	26,25	551,25
C	9	8	23	5	39,2	0,625	24,5	563,5
C	9	8	25	5	37,6	0,625	23,5	587,5

Suplemmentary material

C	9	8	27	5	33	0,625	20,625	556,875
C	9	8	29	5	29,2	0,625	18,25	529,25
C	9	8	31	5	36,2	0,625	22,625	701,375
C	9	8	33	5	26,8	0,625	16,75	552,75
C	9	8	35	4	13,25	0,5	6,625	231,875
C	9	8	37	4	16,5	0,5	8,25	305,25
C	9	8	39	4	7,5	0,5	3,75	146,25
C	9	8	41	2	7	0,25	1,75	71,75
C	9	8	43	2	0,5	0,25	0,125	5,375
C	9	8	45	2	0	0,25	0	0
C	9	8	47	2	0	0,25	0	0
C	9	8	49	2	0	0,25	0	0
C	9	8	51	0	0	0	0	0
C	1	16	3	16	9,5	1	9,5	28,5
C	1	16	5	16	20,3125	1	20,3125	101,5625
C	1	16	7	16	19,6875	1	19,6875	137,8125
C	1	16	9	16	16,25	1	16,25	146,25
C	1	16	11	16	17,5625	1	17,5625	193,1875
C	1	16	13	16	28,5	1	28,5	370,5
C	1	16	15	15	15,33333333	0,9375	14,375	215,625
C	1	16	17	15	15,4	0,9375	14,4375	245,4375
C	1	16	19	14	18,71428571	0,875	16,375	311,125
C	1	16	21	13	16,23076923	0,8125	13,1875	276,9375
C	1	16	23	13	23,30769231	0,8125	18,9375	435,5625
C	1	16	25	13	14,69230769	0,8125	11,9375	298,4375
C	1	16	27	13	14,61538462	0,8125	11,875	320,625
C	1	16	29	13	15,46153846	0,8125	12,5625	364,3125
C	1	16	31	11	16,09090909	0,6875	11,0625	342,9375
C	1	16	33	10	10,1	0,625	6,3125	208,3125
C	1	16	35	10	3,3	0,625	2,0625	72,1875
C	1	16	37	10	5,7	0,625	3,5625	131,8125
C	1	16	39	9	10,66666667	0,5625	6	234
C	1	16	41	9	4,111111111	0,5625	2,3125	94,8125
C	1	16	43	9	1,777777778	0,5625	1	43
C	1	16	45	5	0,8	0,3125	0,25	11,25
C	1	16	47	3	5,666666667	0,1875	1,0625	49,9375
C	1	16	49	2	0	0,125	0	0
C	1	16	51	1	0	0,0625	0	0
C	1	16	53	1	0	0,0625	0	0
C	1	16	55	1	0	0,0625	0	0
C	1	16	57	0	0	0	0	0
C	2	16	3	16	8,75	1	8,75	26,25
C	2	16	5	16	11,8125	1	11,8125	59,0625
C	2	16	7	16	26,75	1	26,75	187,25
C	2	16	9	16	21,5625	1	21,5625	194,0625

Suplemmentary material

C	2	16	11	16	26,4375	1	26,4375	290,8125
C	2	16	13	16	23,75	1	23,75	308,75
C	2	16	15	16	19,75	1	19,75	296,25
C	2	16	17	16	11,375	1	11,375	193,375
C	2	16	19	16	19,9375	1	19,9375	378,8125
C	2	16	21	16	17,875	1	17,875	375,375
C	2	16	23	16	21,375	1	21,375	491,625
C	2	16	25	16	11,8125	1	11,8125	295,3125
C	2	16	27	16	8,5625	1	8,5625	231,1875
C	2	16	29	16	10,375	1	10,375	300,875
C	2	16	31	11	7,272727273	0,6875	5	155
C	2	16	33	11	5,727272727	0,6875	3,9375	129,9375
C	2	16	35	6	12,33333333	0,375	4,625	161,875
C	2	16	37	5	9	0,3125	2,8125	104,0625
C	2	16	39	2	6	0,125	0,75	29,25
C	2	16	41	2	0	0,125	0	0
C	2	16	43	1	0	0,0625	0	0
C	2	16	45	1	0	0,0625	0	0
C	2	16	47	1	0	0,0625	0	0
C	2	16	49	1	0	0,0625	0	0
C	2	16	51	0	0	0	0	0
C	3	16	3	16	21,1875	1	21,1875	63,5625
C	3	16	5	16	20,1875	1	20,1875	100,9375
C	3	16	7	16	21,125	1	21,125	147,875
C	3	16	9	15	29,46666667	0,9375	27,625	248,625
C	3	16	11	15	24,66666667	0,9375	23,125	254,375
C	3	16	13	15	25,13333333	0,9375	23,5625	306,3125
C	3	16	15	15	16,06666667	0,9375	15,0625	225,9375
C	3	16	17	15	20,2	0,9375	18,9375	321,9375
C	3	16	19	14	19,28571429	0,875	16,875	320,625
C	3	16	21	12	17,75	0,75	13,3125	279,5625
C	3	16	23	10	30,2	0,625	18,875	434,125
C	3	16	25	10	24	0,625	15	375
C	3	16	27	10	33	0,625	20,625	556,875
C	3	16	29	10	18,1	0,625	11,3125	328,0625
C	3	16	31	8	16,125	0,5	8,0625	249,9375
C	3	16	33	7	19,57142857	0,4375	8,5625	282,5625
C	3	16	35	7	10,28571429	0,4375	4,5	157,5
C	3	16	37	7	10,28571429	0,4375	4,5	166,5
C	3	16	39	6	10	0,375	3,75	146,25
C	3	16	41	6	11	0,375	4,125	169,125
C	3	16	43	6	3,666666667	0,375	1,375	59,125
C	3	16	45	4	0	0,25	0	0
C	3	16	47	2	0	0,125	0	0
C	3	16	49	2	0,5	0,125	0,0625	3,0625

Suplemmentary material

C	3	16	51	0	0	0	0	0
C	4	16	3	16	19,875	1	19,875	59,625
C	4	16	5	16	15,75	1	15,75	78,75
C	4	16	7	16	17,875	1	17,875	125,125
C	4	16	9	16	20,375	1	20,375	183,375
C	4	16	11	16	17,4375	1	17,4375	191,8125
C	4	16	13	16	16,25	1	16,25	211,25
C	4	16	15	16	16,75	1	16,75	251,25
C	4	16	17	16	14,0625	1	14,0625	239,0625
C	4	16	19	15	21,6	0,9375	20,25	384,75
C	4	16	21	15	29,46666667	0,9375	27,625	580,125
C	4	16	23	15	21,86666667	0,9375	20,5	471,5
C	4	16	25	15	18,46666667	0,9375	17,3125	432,8125
C	4	16	27	15	21,73333333	0,9375	20,375	550,125
C	4	16	29	15	16,8	0,9375	15,75	456,75
C	4	16	31	15	20,46666667	0,9375	19,1875	594,8125
C	4	16	33	15	16,33333333	0,9375	15,3125	505,3125
C	4	16	35	11	10,45454545	0,6875	7,1875	251,5625
C	4	16	37	9	19,22222222	0,5625	10,8125	400,0625
C	4	16	39	8	16,5	0,5	8,25	321,75
C	4	16	41	8	20,875	0,5	10,4375	427,9375
C	4	16	43	8	9,125	0,5	4,5625	196,1875
C	4	16	45	5	3,6	0,3125	1,125	50,625
C	4	16	47	5	6,8	0,3125	2,125	99,875
C	4	16	49	4	2	0,25	0,5	24,5
C	4	16	51	3	1,666666667	0,1875	0,3125	15,9375
C	4	16	52	0	0	0	0	0
C	5	16	3	16	6,75	1	6,75	20,25
C	5	16	5	16	16,625	1	16,625	83,125
C	5	16	7	15	16,66666667	0,9375	15,625	109,375
C	5	16	9	15	22,26666667	0,9375	20,875	187,875
C	5	16	11	15	17,66666667	0,9375	16,5625	182,1875
C	5	16	13	15	30,66666667	0,9375	28,75	373,75
C	5	16	15	15	12,26666667	0,9375	11,5	172,5
C	5	16	17	15	15,93333333	0,9375	14,9375	253,9375
C	5	16	19	13	11	0,8125	8,9375	169,8125
C	5	16	21	13	17,53846154	0,8125	14,25	299,25
C	5	16	23	12	28,66666667	0,75	21,5	494,5
C	5	16	25	12	17,16666667	0,75	12,875	321,875
C	5	16	27	12	25,25	0,75	18,9375	511,3125
C	5	16	29	12	19,83333333	0,75	14,875	431,375
C	5	16	31	10	14	0,625	8,75	271,25
C	5	16	33	10	22,8	0,625	14,25	470,25
C	5	16	35	10	8,9	0,625	5,5625	194,6875
C	5	16	37	10	8,4	0,625	5,25	194,25

Suplemmentary material

C	5	16	39	9	18,77777778	0,5625	10,5625	411,9375
C	5	16	41	9	21,66666667	0,5625	12,1875	499,6875
C	5	16	43	9	16,44444444	0,5625	9,25	397,75
C	5	16	45	9	11	0,5625	6,1875	278,4375
C	5	16	47	7	9,428571429	0,4375	4,125	193,875
C	5	16	49	7	8,571428571	0,4375	3,75	183,75
C	5	16	51	3	12,33333333	0,1875	2,3125	117,9375
C	5	16	53	3	4,333333333	0,1875	0,8125	43,0625
C	5	16	55	3	11,33333333	0,1875	2,125	116,875
C	5	16	57	3	0	0,1875	0	0
C	5	16	59	2	0	0,125	0	0
C	5	16	61	0	0	0	0	0
C	6	16	3	16	6,875	1	6,875	20,625
C	6	16	5	16	21,1875	1	21,1875	105,9375
C	6	16	7	16	15,0625	1	15,0625	105,4375
C	6	16	9	16	32,625	1	32,625	293,625
C	6	16	11	16	29,25	1	29,25	321,75
C	6	16	13	16	13,5	1	13,5	175,5
C	6	16	15	16	17,6875	1	17,6875	265,3125
C	6	16	17	16	12	1	12	204
C	6	16	19	16	17,75	1	17,75	337,25
C	6	16	21	16	13,3125	1	13,3125	279,5625
C	6	16	23	16	27,5625	1	27,5625	633,9375
C	6	16	25	16	13,9375	1	13,9375	348,4375
C	6	16	27	16	24,8125	1	24,8125	669,9375
C	6	16	29	16	18,875	1	18,875	547,375
C	6	16	31	10	23,2	0,625	14,5	449,5
C	6	16	33	10	25,3	0,625	15,8125	521,8125
C	6	16	35	10	16,1	0,625	10,0625	352,1875
C	6	16	37	10	5,2	0,625	3,25	120,25
C	6	16	39	5	20,6	0,3125	6,4375	251,0625
C	6	16	41	5	24	0,3125	7,5	307,5
C	6	16	43	5	10,8	0,3125	3,375	145,125
C	6	16	45	5	2,2	0,3125	0,6875	30,9375
C	6	16	47	3	0	0,1875	0	0
C	6	16	49	2	1	0,125	0,125	6,125
C	6	16	51	1	0	0,0625	0	0
C	6	16	53	0	0	0	0	0
C	8	16	3	16	1,4375	1	1,4375	4,3125
C	8	16	5	16	7,0625	1	7,0625	35,3125
C	8	16	7	16	13,3125	1	13,3125	93,1875
C	8	16	9	16	36,5625	1	36,5625	329,0625
C	8	16	11	16	16,75	1	16,75	184,25
C	8	16	13	16	22,0625	1	22,0625	286,8125
C	8	16	15	16	23,6875	1	23,6875	355,3125

Suplemmentary material

C	8	16	17	16	18,6875	1	18,6875	317,6875
C	8	16	19	16	16,5625	1	16,5625	314,6875
C	8	16	21	16	20,9375	1	20,9375	439,6875
C	8	16	23	16	16,125	1	16,125	370,875
C	8	16	25	16	17,125	1	17,125	428,125
C	8	16	27	16	19,625	1	19,625	529,875
C	8	16	29	16	13,6875	1	13,6875	396,9375
C	8	16	31	15	11,46666667	0,9375	10,75	333,25
C	8	16	33	15	10,66666667	0,9375	10	330
C	8	16	35	15	11,2	0,9375	10,5	367,5
C	8	16	37	15	6,666666667	0,9375	6,25	231,25
C	8	16	39	15	9,8	0,9375	9,1875	358,3125
C	8	16	41	14	10,28571429	0,875	9	369
C	8	16	43	13	11	0,8125	8,9375	384,3125
C	8	16	45	13	7,769230769	0,8125	6,3125	284,0625
C	8	16	47	9	4,333333333	0,5625	2,4375	114,5625
C	8	16	49	7	0	0,4375	0	0
C	8	16	51	3	2	0,1875	0,375	19,125
C	8	16	53	3	0	0,1875	0	0
C	8	16	55	1	0	0,0625	0	0
C	8	16	57	0	0	0	0	0
C	9	16	3	16	13,3125	1	13,3125	39,9375
C	9	16	5	16	13,8125	1	13,8125	69,0625
C	9	16	7	16	25,25	1	25,25	176,75
C	9	16	9	16	32,9375	1	32,9375	296,4375
C	9	16	11	16	27,8125	1	27,8125	305,9375
C	9	16	13	16	22,5625	1	22,5625	293,3125
C	9	16	15	16	20	1	20	300
C	9	16	17	16	23,0625	1	23,0625	392,0625
C	9	16	19	16	24,4375	1	24,4375	464,3125
C	9	16	21	16	21,5	1	21,5	451,5
C	9	16	23	16	22,8125	1	22,8125	524,6875
C	9	16	25	16	27,375	1	27,375	684,375
C	9	16	27	14	18,92857143	0,875	16,5625	447,1875
C	9	16	29	14	16,28571429	0,875	14,25	413,25
C	9	16	31	13	17,53846154	0,8125	14,25	441,75
C	9	16	33	13	8,307692308	0,8125	6,75	222,75
C	9	16	35	13	7,384615385	0,8125	6	210
C	9	16	37	13	2,538461538	0,8125	2,0625	76,3125
C	9	16	39	6	0,5	0,375	0,1875	7,3125
C	9	16	41	5	0,2	0,3125	0,0625	2,5625
C	9	16	43	5	0	0,3125	0	0
C	9	16	45	4	0	0,25	0	0
C	9	16	47	4	0	0,25	0	0
C	9	16	49	4	0	0,25	0	0

Suplemmentary material

C	9	16	51	4	0	0,25	0	0
C	9	16	53	4	0	0,25	0	0
C	9	16	55	3	0	0,1875	0	0
C	9	16	57	0	0	0	0	0
V	1	2	3	2	0	1	0	0
V	1	2	5	2	12,5	1	12,5	62,5
V	1	2	7	2	9,5	1	9,5	66,5
V	1	2	9	2	40,5	1	40,5	364,5
V	1	2	11	2	39,5	1	39,5	434,5
V	1	2	13	2	37,5	1	37,5	487,5
V	1	2	15	2	39	1	39	585
V	1	2	17	2	20,5	1	20,5	348,5
V	1	2	19	2	14	1	14	266
V	1	2	21	2	1,5	1	1,5	31,5
V	1	2	23	1	1	0,5	0,5	11,5
V	1	2	25	1	0	0,5	0	0
V	1	2	27	1	6	0,5	3	81
V	1	2	29	1	9	0,5	4,5	130,5
V	1	2	31	1	0	0,5	0	0
V	1	2	33	1	0	0,5	0	0
V	1	2	35	1	0	0,5	0	0
V	1	2	37	1	0	0,5	0	0
V	1	2	39	1	0	0,5	0	0
V	1	2	41	1	0	0,5	0	0
V	1	2	43	0	0	0	0	0
V	2	2	3	2	3	1	3	9
V	2	2	5	2	7,5	1	7,5	37,5
V	2	2	7	2	5,5	1	5,5	38,5
V	2	2	9	2	6,5	1	6,5	58,5
V	2	2	11	2	1	1	1	11
V	2	2	13	2	1,5	1	1,5	19,5
V	2	2	15	2	0	1	0	0
V	2	2	17	1	11	0,5	5,5	93,5
V	2	2	19	1	3	0,5	1,5	28,5
V	2	2	21	1	21	0,5	10,5	220,5
V	2	2	23	1	20	0,5	10	230
V	2	2	25	1	23	0,5	11,5	287,5
V	2	2	27	1	9	0,5	4,5	121,5
V	2	2	29	1	13	0,5	6,5	188,5
V	2	2	31	0	0	0	0	0
V	3	2	3	2	0	1	0	0
V	3	2	5	2	1,5	1	1,5	7,5
V	3	2	7	2	6,5	1	6,5	45,5
V	3	2	9	2	0,5	1	0,5	4,5
V	3	2	11	2	1,5	1	1,5	16,5

Suplemmentary material

V	3	2	13	2	1	1	1	13
V	3	2	15	1	12	0,5	6	90
V	3	2	17	1	5	0,5	2,5	42,5
V	3	2	19	1	15	0,5	7,5	142,5
V	3	2	21	1	0	0,5	0	0
V	3	2	23	1	0	0,5	0	0
V	3	2	25	1	0	0,5	0	0
V	3	2	27	1	0	0,5	0	0
V	3	2	29	1	0	0,5	0	0
V	3	2	31	0	0	0	0	0
V	4	2	3	2	2,5	1	2,5	7,5
V	4	2	5	2	24,5	1	24,5	122,5
V	4	2	7	2	1,5	1	1,5	10,5
V	4	2	9	2	0	1	0	0
V	4	2	11	1	0	0,5	0	0
V	4	2	13	1	0	0,5	0	0
V	4	2	1	0	0	0	0	0
V	5	2	3	2	3	1	3	9
V	5	2	5	2	11,5	1	11,5	57,5
V	5	2	7	2	14,5	1	14,5	101,5
V	5	2	9	2	8,5	1	8,5	76,5
V	5	2	11	2	13,5	1	13,5	148,5
V	5	2	13	2	14,5	1	14,5	188,5
V	5	2	15	2	7	1	7	105
V	5	2	17	2	17,5	1	17,5	297,5
V	5	2	19	2	11	1	11	209
V	5	2	21	2	11	1	11	231
V	5	2	23	2	6	1	6	138
V	5	2	25	2	2,5	1	2,5	62,5
V	5	2	27	2	0	1	0	0
V	5	2	29	2	0	1	0	0
V	5	2	31	2	0	1	0	0
V	5	2	33	2	0	1	0	0
V	5	2	35	2	0	1	0	0
V	5	2	37	2	0	1	0	0
V	5	2	39	2	0	1	0	0
V	5	2	41	2	0	1	0	0
V	5	2	43	2	0	1	0	0
V	5	2	45	2	0	1	0	0
V	5	2	47	1	0	0,5	0	0
V	5	2	49	1	0	0,5	0	0
V	5	2	51	0	0	0	0	0
V	6	2	3	2	3	1	3	9
V	6	2	5	2	12,5	1	12,5	62,5
V	6	2	7	2	1,5	1	1,5	10,5

Suplemmentary material

V	6	2	9	2	3,5	1	3,5	31,5
V	6	2	11	2	2	1	2	22
V	6	2	13	2	0,5	1	0,5	6,5
V	6	2	15	2	1	1	1	15
V	6	2	17	2	4	1	4	68
V	6	2	19	1	4	0,5	2	38
V	6	2	21	1	3	0,5	1,5	31,5
V	6	2	23	1	0	0,5	0	0
V	6	2	25	1	0	0,5	0	0
V	6	2	27	1	0	0,5	0	0
V	6	2	29	1	0	0,5	0	0
V	6	2	31	1	0	0,5	0	0
V	6	2	33	1	0	0,5	0	0
V	6	2	35	0	0	0	0	0
V	7	2	3	2	0	1	0	0
V	7	2	5	2	28,5	1	28,5	142,5
V	7	2	7	2	20,5	1	20,5	143,5
V	7	2	9	2	43	1	43	387
V	7	2	11	2	25	1	25	275
V	7	2	13	2	28,5	1	28,5	370,5
V	7	2	15	2	21,5	1	21,5	322,5
V	7	2	17	2	14	1	14	238
V	7	2	19	2	6	1	6	114
V	7	2	21	2	0	1	0	0
V	7	2	23	2	0	1	0	0
V	7	2	25	2	0	1	0	0
V	7	2	27	2	0	1	0	0
V	7	2	29	0	0	0	0	0
V	9	2	3	2	0,5	1	0,5	1,5
V	9	2	5	2	2,5	1	2,5	12,5
V	9	2	7	2	1	1	1	7
V	9	2	9	2	2,5	1	2,5	22,5
V	9	2	11	2	0,5	1	0,5	5,5
V	9	2	13	2	4	1	4	52
V	9	2	15	2	10,5	1	10,5	157,5
V	9	2	17	2	1,5	1	1,5	25,5
V	9	2	19	1	3	0,5	1,5	28,5
V	9	2	21	1	12	0,5	6	126
V	9	2	23	1	7	0,5	3,5	80,5
V	9	2	25	1	7	0,5	3,5	87,5
V	9	2	27	1	13	0,5	6,5	175,5
V	9	2	29	1	11	0,5	5,5	159,5
V	9	2	31	1	7	0,5	3,5	108,5
V	9	2	33	1	4	0,5	2	66
V	9	2	35	1	0	0,5	0	0

Suplemmentary material

V	9	2	37	1	0	0,5	0	0
V	9	2	39	0	0	0	0	0
V	10	2	3	2	0	1	0	0
V	10	2	5	2	30	1	30	150
V	10	2	7	2	27	1	27	189
V	10	2	9	2	30	1	30	270
V	10	2	11	2	43,5	1	43,5	478,5
V	10	2	13	2	42,5	1	42,5	552,5
V	10	2	15	2	42,5	1	42,5	637,5
V	10	2	17	2	11,5	1	11,5	195,5
V	10	2	19	2	16,5	1	16,5	313,5
V	10	2	21	2	5	1	5	105
V	10	2	23	2	0	1	0	0
V	10	2	25	2	0,5	1	0,5	12,5
V	10	2	27	2	0	1	0	0
V	10	2	29	2	0	1	0	0
V	10	2	31	2	0	1	0	0
V	10	2	33	2	0	1	0	0
V	10	2	35	0	0	0	0	0
V	1	4	3	4	2,5	1	2,5	7,5
V	1	4	5	4	18,75	1	18,75	93,75
V	1	4	7	4	46,25	1	46,25	323,75
V	1	4	9	4	27,5	1	27,5	247,5
V	1	4	11	4	41,5	1	41,5	456,5
V	1	4	13	4	35,25	1	35,25	458,25
V	1	4	15	4	30,25	1	30,25	453,75
V	1	4	17	4	21,5	1	21,5	365,5
V	1	4	19	4	17,75	1	17,75	337,25
V	1	4	21	4	2,5	1	2,5	52,5
V	1	4	23	4	4,75	1	4,75	109,25
V	1	4	25	3	0	0,75	0	0
V	1	4	27	3	0	0,75	0	0
V	1	4	29	2	0	0,5	0	0
V	1	4	31	2	0	0,5	0	0
V	1	4	33	2	0	0,5	0	0
V	1	4	35	0	0	0	0	0
V	2	4	3	4	6,25	1	6,25	18,75
V	2	4	5	4	23,25	1	23,25	116,25
V	2	4	7	4	32,75	1	32,75	229,25
V	2	4	9	4	14,75	1	14,75	132,75
V	2	4	11	4	40	1	40	440
V	2	4	13	4	35,25	1	35,25	458,25
V	2	4	15	4	32,25	1	32,25	483,75
V	2	4	17	4	8,5	1	8,5	144,5
V	2	4	19	4	15	1	15	285

Suplemmentary material

V	2	4	21	4	7,5	1	7,5	157,5
V	2	4	23	2	3,5	0,5	1,75	40,25
V	2	4	25	2	4,5	0,5	2,25	56,25
V	2	4	27	2	6,5	0,5	3,25	87,75
V	2	4	29	2	7,5	0,5	3,75	108,75
V	2	4	31	2	3	0,5	1,5	46,5
V	2	4	33	0	0	0	0	0
V	3	4	3	4	1,25	1	1,25	3,75
V	3	4	5	4	13	1	13	65
V	3	4	7	4	25,25	1	25,25	176,75
V	3	4	9	4	20,5	1	20,5	184,5
V	3	4	11	4	25	1	25	275
V	3	4	13	4	24,75	1	24,75	321,75
V	3	4	15	4	16,25	1	16,25	243,75
V	3	4	17	4	12	1	12	204
V	3	4	19	4	5,5	1	5,5	104,5
V	3	4	21	4	0	1	0	0
V	3	4	23	4	0	1	0	0
V	3	4	25	2	0	0,5	0	0
V	3	4	27	2	0	0,5	0	0
V	3	4	29	2	0	0,5	0	0
V	3	4	31	0	0	0	0	0
V	4	4	3	4	1,75	1	1,75	5,25
V	4	4	5	4	9	1	9	45
V	4	4	7	4	7,75	1	7,75	54,25
V	4	4	9	4	12,25	1	12,25	110,25
V	4	4	11	4	42,5	1	42,5	467,5
V	4	4	13	4	23	1	23	299
V	4	4	15	2	63,5	0,5	31,75	476,25
V	4	4	17	2	73	0,5	36,5	620,5
V	4	4	19	2	51	0,5	25,5	484,5
V	4	4	21	2	43,5	0,5	21,75	456,75
V	4	4	23	2	32,5	0,5	16,25	373,75
V	4	4	25	1	36	0,25	9	225
V	4	4	27	1	17	0,25	4,25	114,75
V	4	4	29	1	16	0,25	4	116
V	4	4	31	1	0	0,25	0	0
V	4	4	33	1	0	0,25	0	0
V	4	4	35	1	0	0,25	0	0
V	4	4	37	1	0	0,25	0	0
V	4	4	39	1	0	0,25	0	0
V	4	4	41	1	0	0,25	0	0
V	4	4	43	0	0	0	0	0
V	5	4	3	4	0	1	0	0
V	5	4	5	4	6,5	1	6,5	32,5

Suplemmentary material

V	5	4	7	4	17	1	17	119
V	5	4	9	4	10,75	1	10,75	96,75
V	5	4	11	3	0,333333333	0,75	0,25	2,75
V	5	4	13	3	0,333333333	0,75	0,25	3,25
V	5	4	15	2	1,5	0,5	0,75	11,25
V	5	4	17	2	2,5	0,5	1,25	21,25
V	5	4	19	2	1	0,5	0,5	9,5
V	5	4	21	1	0	0,25	0	0
V	5	4	23	0	0	0	0	0
V	6	4	3	4	0,5	1	0,5	1,5
V	6	4	5	4	8	1	8	40
V	6	4	7	4	9,25	1	9,25	64,75
V	6	4	9	4	17,5	1	17,5	157,5
V	6	4	11	4	22	1	22	242
V	6	4	13	4	18,75	1	18,75	243,75
V	6	4	15	3	18,66666667	0,75	14	210
V	6	4	17	2	15,5	0,5	7,75	131,75
V	6	4	19	2	20	0,5	10	190
V	6	4	21	2	17	0,5	8,5	178,5
V	6	4	23	2	18,5	0,5	9,25	212,75
V	6	4	25	2	9	0,5	4,5	112,5
V	6	4	27	2	0	0,5	0	0
V	6	4	29	1	1	0,25	0,25	7,25
V	6	4	31	1	0	0,25	0	0
V	6	4	33	0	0	0	0	0
V	10	4	3	4	0	1	0	0
V	10	4	5	4	20,75	1	20,75	103,75
V	10	4	7	4	4,25	1	4,25	29,75
V	10	4	9	4	18,5	1	18,5	166,5
V	10	4	11	4	15,25	1	15,25	167,75
V	10	4	13	4	18	1	18	234
V	10	4	15	3	11	0,75	8,25	123,75
V	10	4	17	3	19,33333333	0,75	14,5	246,5
V	10	4	19	3	24,33333333	0,75	18,25	346,75
V	10	4	21	3	27,66666667	0,75	20,75	435,75
V	10	4	23	3	16,66666667	0,75	12,5	287,5
V	10	4	25	3	6,333333333	0,75	4,75	118,75
V	10	4	27	3	0	0,75	0	0
V	10	4	29	1	0	0,25	0	0
V	10	4	31	0	0	0	0	0
V	1	8	3	8	2,75	1	2,75	8,25
V	1	8	5	8	4,25	1	4,25	21,25
V	1	8	7	8	22,75	1	22,75	159,25
V	1	8	9	8	26,75	1	26,75	240,75
V	1	8	11	8	25,625	1	25,625	281,875

Suplemmentary material

V	1	8	13	8	13,625	1	13,625	177,125
V	1	8	15	8	22,75	1	22,75	341,25
V	1	8	17	8	13,5	1	13,5	229,5
V	1	8	19	8	10,125	1	10,125	192,375
V	1	8	21	8	4,875	1	4,875	102,375
V	1	8	23	8	0,5	1	0,5	11,5
V	1	8	25	7	0,428571429	0,875	0,375	9,375
V	1	8	27	7	0,142857143	0,875	0,125	3,375
V	1	8	29	5	4	0,625	2,5	72,5
V	1	8	31	4	0,5	0,5	0,25	7,75
V	1	8	33	3	0	0,375	0	0
V	1	8	35	3	0	0,375	0	0
V	1	8	37	2	0	0,25	0	0
V	1	8	39	2	0	0,25	0	0
V	1	8	41	0	0	0	0	0
V	2	8	3	8	1,125	1	1,125	3,375
V	2	8	5	8	6,25	1	6,25	31,25
V	2	8	7	8	8,875	1	8,875	62,125
V	2	8	9	8	8	1	8	72
V	2	8	11	8	8,25	1	8,25	90,75
V	2	8	13	8	12,625	1	12,625	164,125
V	2	8	15	8	11,5	1	11,5	172,5
V	2	8	17	8	7,5	1	7,5	127,5
V	2	8	19	8	7	1	7	133
V	2	8	21	8	8,25	1	8,25	173,25
V	2	8	23	8	2,625	1	2,625	60,375
V	2	8	25	8	2,25	1	2,25	56,25
V	2	8	27	8	2	1	2	54
V	2	8	29	8	0,625	1	0,625	18,125
V	2	8	31	3	0	0,375	0	0
V	2	8	33	2	0	0,25	0	0
V	2	8	35	1	0	0,125	0	0
V	2	8	37	0	0	0	0	0
V	3	8	3	8	1,625	1	1,625	4,875
V	3	8	5	8	6,125	1	6,125	30,625
V	3	8	7	8	22,5	1	22,5	157,5
V	3	8	9	8	16	1	16	144
V	3	8	11	8	22,875	1	22,875	251,625
V	3	8	13	8	22	1	22	286
V	3	8	15	8	19,5	1	19,5	292,5
V	3	8	17	8	19,625	1	19,625	333,625
V	3	8	19	8	19,875	1	19,875	377,625
V	3	8	21	8	11,25	1	11,25	236,25
V	3	8	23	8	10,25	1	10,25	235,75
V	3	8	25	8	6	1	6	150

Suplemmentary material

V	3	8	27	8	4,75	1	4,75	128,25
V	3	8	29	7	5,714285714	0,875	5	145
V	3	8	31	6	4,833333333	0,75	3,625	112,375
V	3	8	33	4	4,75	0,5	2,375	78,375
V	3	8	35	4	1,25	0,5	0,625	21,875
V	3	8	37	3	0	0,375	0	0
V	3	8	39	2	0	0,25	0	0
V	3	8	41	1	0	0,125	0	0
V	3	8	43	1	0	0,125	0	0
V	3	8	45	0	0	0	0	0
V	4	8	3	8	1,125	1	1,125	3,375
V	4	8	5	8	8,5	1	8,5	42,5
V	4	8	7	8	9,25	1	9,25	64,75
V	4	8	9	8	22,125	1	22,125	199,125
V	4	8	11	8	20,25	1	20,25	222,75
V	4	8	13	8	11,125	1	11,125	144,625
V	4	8	15	7	11,71428571	0,875	10,25	153,75
V	4	8	17	7	2,142857143	0,875	1,875	31,875
V	4	8	19	7	3,285714286	0,875	2,875	54,625
V	4	8	21	7	2,428571429	0,875	2,125	44,625
V	4	8	23	7	2,142857143	0,875	1,875	43,125
V	4	8	25	4	5,75	0,5	2,875	71,875
V	4	8	27	4	6,25	0,5	3,125	84,375
V	4	8	29	4	6,25	0,5	3,125	90,625
V	4	8	31	3	3,666666667	0,375	1,375	42,625
V	4	8	33	2	0	0,25	0	0
V	4	8	35	2	0	0,25	0	0
V	4	8	37	2	0	0,25	0	0
V	4	8	39	1	0	0,125	0	0
V	4	8	41	1	0	0,125	0	0
V	4	8	43	0	0	0	0	0
V	5	8	3	8	0,625	1	0,625	1,875
V	5	8	5	6	7,333333333	0,75	5,5	27,5
V	5	8	7	6	5,333333333	0,75	4	28
V	5	8	9	6	14,66666667	0,75	11	99
V	5	8	11	6	24,66666667	0,75	18,5	203,5
V	5	8	13	6	4,333333333	0,75	3,25	42,25
V	5	8	15	6	25,33333333	0,75	19	285
V	5	8	17	6	26,16666667	0,75	19,625	333,625
V	5	8	19	5	11,6	0,625	7,25	137,75
V	5	8	21	5	6,2	0,625	3,875	81,375
V	5	8	23	5	3,2	0,625	2	46
V	5	8	25	5	1,4	0,625	0,875	21,875
V	5	8	27	5	2,8	0,625	1,75	47,25
V	5	8	29	5	0,4	0,625	0,25	7,25

Suplemmentary material

V	5	8	31	5	0,6	0,625	0,375	11,625
V	5	8	33	5	0	0,625	0	0
V	5	8	35	4	0	0,5	0	0
V	5	8	37	3	0	0,375	0	0
V	5	8	39	2	0	0,25	0	0
V	5	8	41	1	0	0,125	0	0
V	5	8	43	1	0	0,125	0	0
V	5	8	45	0	0	0	0	0
V	6	8	3	8	1,75	1	1,75	5,25
V	6	8	5	8	15,875	1	15,875	79,375
V	6	8	7	8	12,625	1	12,625	88,375
V	6	8	9	8	15,75	1	15,75	141,75
V	6	8	11	8	11,5	1	11,5	126,5
V	6	8	13	8	12,25	1	12,25	159,25
V	6	8	15	8	3,625	1	3,625	54,375
V	6	8	17	6	9,833333333	0,75	7,375	125,375
V	6	8	19	6	5,166666667	0,75	3,875	73,625
V	6	8	21	6	3,166666667	0,75	2,375	49,875
V	6	8	23	5	1	0,625	0,625	14,375
V	6	8	25	5	0,2	0,625	0,125	3,125
V	6	8	27	4	0,5	0,5	0,25	6,75
V	6	8	29	4	0	0,5	0	0
V	6	8	31	1	0	0,125	0	0
V	6	8	33	1	0	0,125	0	0
V	6	8	35	0	0	0	0	0
V	7	8	3	8	0,125	1	0,125	0,375
V	7	8	5	8	13,5	1	13,5	67,5
V	7	8	7	8	16,875	1	16,875	118,125
V	7	8	9	8	8,375	1	8,375	75,375
V	7	8	11	8	5,125	1	5,125	56,375
V	7	8	13	8	3,75	1	3,75	48,75
V	7	8	15	8	2,875	1	2,875	43,125
V	7	8	17	6	7,5	0,75	5,625	95,625
V	7	8	19	6	11,66666667	0,75	8,75	166,25
V	7	8	21	6	6,833333333	0,75	5,125	107,625
V	7	8	23	6	7	0,75	5,25	120,75
V	7	8	25	5	3,8	0,625	2,375	59,375
V	7	8	27	5	0,4	0,625	0,25	6,75
V	7	8	29	5	0	0,625	0	0
V	7	8	31	2	2	0,25	0,5	15,5
V	7	8	33	2	1,5	0,25	0,375	12,375
V	7	8	35	1	1	0,125	0,125	4,375
V	7	8	37	1	0	0,125	0	0
V	7	8	39	1	0	0,125	0	0
V	7	8	41	0	0	0	0	0

Suplemmentary material

V	8	8	3	8	0,25	1	0,25	0,75
V	8	8	5	8	4,375	1	4,375	21,875
V	8	8	7	8	9,125	1	9,125	63,875
V	8	8	9	8	16	1	16	144
V	8	8	11	8	30,125	1	30,125	331,375
V	8	8	13	8	11,75	1	11,75	152,75
V	8	8	15	8	12,25	1	12,25	183,75
V	8	8	17	8	10,75	1	10,75	182,75
V	8	8	19	8	6,625	1	6,625	125,875
V	8	8	21	8	4,5	1	4,5	94,5
V	8	8	23	8	3,625	1	3,625	83,375
V	8	8	25	7	3	0,875	2,625	65,625
V	8	8	27	6	2,666666667	0,75	2	54
V	8	8	29	6	3,333333333	0,75	2,5	72,5
V	8	8	31	5	2,8	0,625	1,75	54,25
V	8	8	33	5	0	0,625	0	0
V	8	8	35	3	0	0,375	0	0
V	8	8	37	3	0	0,375	0	0
V	8	8	39	0	0	0	0	0
V	9	8	3	8	0,25	1	0,25	0,75
V	9	8	5	8	9,125	1	9,125	45,625
V	9	8	7	8	5,625	1	5,625	39,375
V	9	8	9	8	9,625	1	9,625	86,625
V	9	8	11	8	8,625	1	8,625	94,875
V	9	8	13	5	15,2	0,625	9,5	123,5
V	9	8	15	5	12	0,625	7,5	112,5
V	9	8	17	5	9,2	0,625	5,75	97,75
V	9	8	19	4	11,5	0,5	5,75	109,25
V	9	8	21	4	10	0,5	5	105
V	9	8	23	3	1	0,375	0,375	8,625
V	9	8	25	3	2	0,375	0,75	18,75
V	9	8	27	3	0	0,375	0	0
V	9	8	29	3	0	0,375	0	0
V	9	8	31	3	0	0,375	0	0
V	9	8	33	3	0	0,375	0	0
V	9	8	35	0	0	0	0	0
V	10	8	3	8	1,375	1	1,375	4,125
V	10	8	5	8	9,375	1	9,375	46,875
V	10	8	7	8	19,875	1	19,875	139,125
V	10	8	9	8	15,875	1	15,875	142,875
V	10	8	11	8	16,875	1	16,875	185,625
V	10	8	13	8	20,625	1	20,625	268,125
V	10	8	15	8	15	1	15	225
V	10	8	17	8	16,375	1	16,375	278,375
V	10	8	19	7	19,71428571	0,875	17,25	327,75

Suplemmentary material

V	10	8	21	7	9,285714286	0,875	8,125	170,625
V	10	8	23	7	4,714285714	0,875	4,125	94,875
V	10	8	25	6	3,5	0,75	2,625	65,625
V	10	8	27	6	4,5	0,75	3,375	91,125
V	10	8	29	4	0	0,5	0	0
V	10	8	31	4	0	0,5	0	0
V	10	8	33	4	0	0,5	0	0
V	10	8	35	4	0	0,5	0	0
V	10	8	37	0	0	0	0	0
V	1	16	3	16	0	1	0	0
V	1	16	5	13	9,307692308	0,8125	7,5625	37,8125
V	1	16	7	13	13,30769231	0,8125	10,8125	75,6875
V	1	16	9	13	10,84615385	0,8125	8,8125	79,3125
V	1	16	11	13	13	0,8125	10,5625	116,1875
V	1	16	13	13	13,53846154	0,8125	11	143
V	1	16	15	13	7,384615385	0,8125	6	90
V	1	16	17	12	9,166666667	0,75	6,875	116,875
V	1	16	19	12	4,5	0,75	3,375	64,125
V	1	16	21	11	4,272727273	0,6875	2,9375	61,6875
V	1	16	23	11	4,545454545	0,6875	3,125	71,875
V	1	16	25	10	0,5	0,625	0,3125	7,8125
V	1	16	27	8	3	0,5	1,5	40,5
V	1	16	29	8	0,375	0,5	0,1875	5,4375
V	1	16	31	8	0,25	0,5	0,125	3,875
V	1	16	33	6	0	0,375	0	0
V	1	16	35	5	0	0,3125	0	0
V	1	16	37	3	0	0,1875	0	0
V	1	16	39	1	0	0,0625	0	0
V	1	16	41	1	0	0,0625	0	0
V	1	16	43	0	0	0	0	0
V	1	16	45	0	0	0	0	0
V	1	16	47	0	0	0	0	0
V	1	16	49	0	0	0	0	0
V	1	16	51	0	0	0	0	0
V	1	16	53	0	0	0	0	0
V	1	16	55	0	0	0	0	0
V	1	16	57	0	0	0	0	0
V	2	16	3	16	0	1	0	0
V	2	16	5	15	2,466666667	0,9375	2,3125	11,5625
V	2	16	7	14	6,142857143	0,875	5,375	37,625
V	2	16	9	14	6,928571429	0,875	6,0625	54,5625
V	2	16	11	14	7	0,875	6,125	67,375
V	2	16	13	14	4,785714286	0,875	4,1875	54,4375
V	2	16	15	14	4,285714286	0,875	3,75	56,25
V	2	16	17	14	2,214285714	0,875	1,9375	32,9375

Suplemmentary material

V	2	16	19	13	3,230769231	0,8125	2,625	49,875
V	2	16	21	13	0,615384615	0,8125	0,5	10,5
V	2	16	23	12	1,166666667	0,75	0,875	20,125
V	2	16	25	9	0,444444444	0,5625	0,25	6,25
V	2	16	27	9	1	0,5625	0,5625	15,1875
V	2	16	29	8	0,875	0,5	0,4375	12,6875
V	2	16	31	7	2	0,4375	0,875	27,125
V	2	16	33	6	4,166666667	0,375	1,5625	51,5625
V	2	16	35	6	0,166666667	0,375	0,0625	2,1875
V	2	16	37	4	0	0,25	0	0
V	2	16	39	4	0	0,25	0	0
V	2	16	41	1	0	0,0625	0	0
V	2	16	43	1	0	0,0625	0	0
V	2	16	45	1	0	0,0625	0	0
V	2	16	47	0	0	0	0	0
V	3	16	3	15	0	0,9375	0	0
V	3	16	5	14	3,928571429	0,875	3,4375	17,1875
V	3	16	7	14	9	0,875	7,875	55,125
V	3	16	9	14	8,785714286	0,875	7,6875	69,1875
V	3	16	11	14	9,285714286	0,875	8,125	89,375
V	3	16	13	14	8,928571429	0,875	7,8125	101,5625
V	3	16	15	12	4,25	0,75	3,1875	47,8125
V	3	16	17	12	5	0,75	3,75	63,75
V	3	16	19	10	2,6	0,625	1,625	30,875
V	3	16	21	10	1,8	0,625	1,125	23,625
V	3	16	23	10	2,3	0,625	1,4375	33,0625
V	3	16	25	8	0,5	0,5	0,25	6,25
V	3	16	27	8	0	0,5	0	0
V	3	16	29	7	0	0,4375	0	0
V	3	16	31	6	0	0,375	0	0
V	3	16	33	4	0	0,25	0	0
V	3	16	35	4	0	0,25	0	0
V	3	16	37	2	0	0,125	0	0
V	3	16	39	0	0	0	0	0
V	4	16	3	16	0,1875	1	0,1875	0,5625
V	4	16	5	13	5,615384615	0,8125	4,5625	22,8125
V	4	16	7	13	8,230769231	0,8125	6,6875	46,8125
V	4	16	9	12	11,66666667	0,75	8,75	78,75
V	4	16	11	12	8,333333333	0,75	6,25	68,75
V	4	16	13	11	6,181818182	0,6875	4,25	55,25
V	4	16	15	11	2,363636364	0,6875	1,625	24,375
V	4	16	17	10	0,8	0,625	0,5	8,5
V	4	16	19	10	1,1	0,625	0,6875	13,0625
V	4	16	21	8	0,5	0,5	0,25	5,25
V	4	16	23	8	0,625	0,5	0,3125	7,1875

Suplemmentary material

V	4	16	25	6	0,166666667	0,375	0,0625	1,5625
V	4	16	27	5	0	0,3125	0	0
V	4	16	29	5	0	0,3125	0	0
V	4	16	31	4	0	0,25	0	0
V	4	16	33	4	0	0,25	0	0
V	4	16	35	4	0	0,25	0	0
V	4	16	37	4	0	0,25	0	0
V	4	16	39	3	0	0,1875	0	0
V	4	16	41	3	0	0,1875	0	0
V	4	16	43	2	0	0,125	0	0
V	4	16	45	2	0	0,125	0	0
V	4	16	47	1	0	0,0625	0	0
V	4	16	49	1	0	0,0625	0	0
V	4	16	51	0	0	0	0	0
V	5	16	3	16	4,1875	1	4,1875	12,5625
V	5	16	5	16	15,3125	1	15,3125	76,5625
V	5	16	7	15	17,53333333	0,9375	16,4375	115,0625
V	5	16	9	15	23,4	0,9375	21,9375	197,4375
V	5	16	11	15	26,2	0,9375	24,5625	270,1875
V	5	16	13	15	22,13333333	0,9375	20,75	269,75
V	5	16	15	15	16,13333333	0,9375	15,125	226,875
V	5	16	17	13	19,15384615	0,8125	15,5625	264,5625
V	5	16	19	13	11,92307692	0,8125	9,6875	184,0625
V	5	16	21	12	5,416666667	0,75	4,0625	85,3125
V	5	16	23	12	6,75	0,75	5,0625	116,4375
V	5	16	25	12	5,583333333	0,75	4,1875	104,6875
V	5	16	27	10	4	0,625	2,5	67,5
V	5	16	29	9	3,777777778	0,5625	2,125	61,625
V	5	16	31	8	3,625	0,5	1,8125	56,1875
V	5	16	33	7	1,714285714	0,4375	0,75	24,75
V	5	16	35	5	0,2	0,3125	0,0625	2,1875
V	5	16	37	5	0	0,3125	0	0
V	5	16	39	3	0	0,1875	0	0
V	5	16	41	1	0	0,0625	0	0
V	5	16	43	1	0	0,0625	0	0
V	5	16	45	0	0	0	0	0
V	6	16	3	16	0,0625	1	0,0625	0,1875
V	6	16	5	16	0,5	1	0,5	2,5
V	6	16	7	14	1,642857143	0,875	1,4375	10,0625
V	6	16	9	14	4,428571429	0,875	3,875	34,875
V	6	16	11	14	0,5	0,875	0,4375	4,8125
V	6	16	13	12	4,583333333	0,75	3,4375	44,6875
V	6	16	15	11	3,363636364	0,6875	2,3125	34,6875
V	6	16	17	11	4,636363636	0,6875	3,1875	54,1875
V	6	16	19	9	5	0,5625	2,8125	53,4375

Suplemmentary material

V	6	16	21	9	0,666666667	0,5625	0,375	7,875
V	6	16	23	9	2	0,5625	1,125	25,875
V	6	16	25	6	1,833333333	0,375	0,6875	17,1875
V	6	16	27	6	1,666666667	0,375	0,625	16,875
V	6	16	29	5	4,8	0,3125	1,5	43,5
V	6	16	31	5	2,2	0,3125	0,6875	21,3125
V	6	16	33	3	6	0,1875	1,125	37,125
V	6	16	35	3	3,333333333	0,1875	0,625	21,875
V	6	16	37	2	0	0,125	0	0
V	6	16	39	2	5,5	0,125	0,6875	26,8125
V	6	16	41	2	0,5	0,125	0,0625	2,5625
V	6	16	43	1	0	0,0625	0	0
V	6	16	45	1	0	0,0625	0	0
V	6	16	47	1	0	0,0625	0	0
V	6	16	49	0	0	0	0	0
V	7	16	3	16	0,1875	1	0,1875	0,5625
V	7	16	5	16	2,5625	1	2,5625	12,8125
V	7	16	7	16	7,625	1	7,625	53,375
V	7	16	9	16	5,4375	1	5,4375	48,9375
V	7	16	11	16	8,6875	1	8,6875	95,5625
V	7	16	13	16	2,9375	1	2,9375	38,1875
V	7	16	15	16	5,3125	1	5,3125	79,6875
V	7	16	17	13	4,769230769	0,8125	3,875	65,875
V	7	16	19	12	1,083333333	0,75	0,8125	15,4375
V	7	16	21	12	1,5	0,75	1,125	23,625
V	7	16	23	9	0,666666667	0,5625	0,375	8,625
V	7	16	25	8	0,5	0,5	0,25	6,25
V	7	16	27	7	0	0,4375	0	0
V	7	16	29	6	0,333333333	0,375	0,125	3,625
V	7	16	31	4	0	0,25	0	0
V	7	16	33	1	0	0,0625	0	0
V	7	16	35	0	0	0	0	0
V	8	16	3	16	0,75	1	0,75	2,25
V	8	16	5	16	5,4375	1	5,4375	27,1875
V	8	16	7	16	7,4375	1	7,4375	52,0625
V	8	16	9	16	10,875	1	10,875	97,875
V	8	16	11	16	9,1875	1	9,1875	101,0625
V	8	16	13	16	9,5	1	9,5	123,5
V	8	16	15	16	8,125	1	8,125	121,875
V	8	16	17	16	10,75	1	10,75	182,75
V	8	16	19	14	7	0,875	6,125	116,375
V	8	16	21	14	4,285714286	0,875	3,75	78,75
V	8	16	23	14	2,285714286	0,875	2	46
V	8	16	25	14	2,428571429	0,875	2,125	53,125
V	8	16	27	13	0,923076923	0,8125	0,75	20,25

Suplemmentary material

V	8	16	29	12	3,583333333	0,75	2,6875	77,9375
V	8	16	31	9	0	0,5625	0	0
V	8	16	33	6	2,166666667	0,375	0,8125	26,8125
V	8	16	35	3	0	0,1875	0	0
V	8	16	37	3	0	0,1875	0	0
V	8	16	39	0	0	0	0	0
V	10	16	3	16	0,1875	1	0,1875	0,5625
V	10	16	5	16	9,25	1	9,25	46,25
V	10	16	7	16	10	1	10	70
V	10	16	9	15	12,4	0,9375	11,625	104,625
V	10	16	11	15	16,46666667	0,9375	15,4375	169,8125
V	10	16	13	14	16,57142857	0,875	14,5	188,5
V	10	16	15	14	14	0,875	12,25	183,75
V	10	16	17	14	10,21428571	0,875	8,9375	151,9375
V	10	16	19	14	5,5	0,875	4,8125	91,4375
V	10	16	21	14	2,5	0,875	2,1875	45,9375
V	10	16	23	14	1,571428571	0,875	1,375	31,625
V	10	16	25	12	1,75	0,75	1,3125	32,8125
V	10	16	27	12	0,916666667	0,75	0,6875	18,5625
V	10	16	29	10	0	0,625	0	0
V	10	16	31	10	0	0,625	0	0
V	10	16	33	7	0	0,4375	0	0
V	10	16	35	4	0	0,25	0	0
V	10	16	37	2	0	0,125	0	0
V	10	16	39	0	0	0	0	0
CC	1	2	3	2	0	1	0	0
CC	1	2	5	2	12,5	1	12,5	62,5
CC	1	2	7	2	11	1	11	77
CC	1	2	9	2	6,5	1	6,5	58,5
CC	1	2	11	2	2,5	1	2,5	27,5
CC	1	2	13	1	1	0,5	0,5	6,5
CC	1	2	15	1	1	0,5	0,5	7,5
CC	1	2	17	1	0	0,5	0	0
CC	1	2	1	0	0	0	0	0
CC	2	2	3	2	24	1	24	72
CC	2	2	5	2	33	1	33	165
CC	2	2	7	2	28	1	28	196
CC	2	2	9	2	31	1	31	279
CC	2	2	11	2	35,5	1	35,5	390,5
CC	2	2	13	2	25	1	25	325
CC	2	2	15	2	38	1	38	570
CC	2	2	17	2	15	1	15	255
CC	2	2	19	2	0	1	0	0
CC	2	2	21	1	2	0,5	1	21
CC	2	2	23	0	0	0	0	0

Suplemmentary material

CC	4	2	3	2	18,5	1	18,5	55,5
CC	4	2	5	2	15	1	15	75
CC	4	2	7	2	34	1	34	238
CC	4	2	9	2	37,5	1	37,5	337,5
CC	4	2	11	2	44	1	44	484
CC	4	2	13	2	38,5	1	38,5	500,5
CC	4	2	15	2	47	1	47	705
CC	4	2	17	2	20,5	1	20,5	348,5
CC	4	2	19	2	28,5	1	28,5	541,5
CC	4	2	21	2	14	1	14	294
CC	4	2	23	2	13,5	1	13,5	310,5
CC	4	2	25	2	5	1	5	125
CC	4	2	27	2	3,5	1	3,5	94,5
CC	4	2	29	2	0	1	0	0
CC	4	2	31	2	0	1	0	0
CC	4	2	33	2	0	1	0	0
CC	4	2	35	1	0	0,5	0	0
CC	4	2	37	1	0	0,5	0	0
CC	4	2	39	1	0	0,5	0	0
CC	4	2	41	1	0	0,5	0	0
CC	4	2	43	1	0	0,5	0	0
CC	4	2	45	1	0	0,5	0	0
CC	4	2	47	0	0	0	0	0
CC	5	2	3	2	0	1	0	0
CC	5	2	5	2	64	1	64	320
CC	5	2	7	2	67	1	67	469
CC	5	2	9	2	35,5	1	35,5	319,5
CC	5	2	11	2	35	1	35	385
CC	5	2	13	2	33	1	33	429
CC	5	2	15	2	27	1	27	405
CC	5	2	17	1	52	0,5	26	442
CC	5	2	19	1	54	0,5	27	513
CC	5	2	21	1	43	0,5	21,5	451,5
CC	5	2	23	1	2	0,5	1	23
CC	5	2	25	0	0	0	0	0
CC	6	2	3	2	25	1	25	75
CC	6	2	5	2	88,5	1	88,5	442,5
CC	6	2	7	2	65	1	65	455
CC	6	2	9	2	54,5	1	54,5	490,5
CC	6	2	11	2	45	1	45	495
CC	6	2	13	2	40,5	1	40,5	526,5
CC	6	2	15	2	24	1	24	360
CC	6	2	17	2	40	1	40	680
CC	6	2	19	2	22	1	22	418
CC	6	2	21	2	32	1	32	672

Suplemmentary material

CC	6	2	23	2	34	1	34	782
CC	6	2	25	1	0	0,5	0	0
CC	6	2	27	0	0	0	0	0
CC	7	2	3	2	25	1	25	75
CC	7	2	5	2	37	1	37	185
CC	7	2	7	2	67	1	67	469
CC	7	2	9	2	62,5	1	62,5	562,5
CC	7	2	11	2	52,5	1	52,5	577,5
CC	7	2	13	2	51	1	51	663
CC	7	2	15	2	54	1	54	810
CC	7	2	17	2	45	1	45	765
CC	7	2	19	2	35,5	1	35,5	674,5
CC	7	2	21	2	55	1	55	1155
CC	7	2	23	2	45,5	1	45,5	1046,5
CC	7	2	25	2	35	1	35	875
CC	7	2	27	2	30,5	1	30,5	823,5
CC	7	2	29	1	17	0,5	8,5	246,5
CC	7	2	31	1	28	0,5	14	434
CC	7	2	33	1	4	0,5	2	66
CC	7	2	35	1	2	0,5	1	35
CC	7	2	37	0	0	0	0	0
CC	10	2	3	2	0,5	1	0,5	1,5
CC	10	2	5	2	40	1	40	200
CC	10	2	7	2	54,5	1	54,5	381,5
CC	10	2	9	2	50,5	1	50,5	454,5
CC	10	2	11	2	64	1	64	704
CC	10	2	13	2	39,5	1	39,5	513,5
CC	10	2	15	2	35	1	35	525
CC	10	2	17	2	32	1	32	544
CC	10	2	19	2	25	1	25	475
CC	10	2	21	2	25,5	1	25,5	535,5
CC	10	2	23	2	23,5	1	23,5	540,5
CC	10	2	25	2	28	1	28	700
CC	10	2	27	2	21	1	21	567
CC	10	2	29	2	23	1	23	667
CC	10	2	31	1	45	0,5	22,5	697,5
CC	10	2	33	1	47	0,5	23,5	775,5
CC	10	2	35	1	29	0,5	14,5	507,5
CC	10	2	37	1	6	0,5	3	111
CC	10	2	39	1	2	0,5	1	39
CC	10	2	41	1	0	0,5	0	0
CC	10	2	43	1	0	0,5	0	0
CC	10	2	45	1	0	0,5	0	0
CC	10	2	47	1	0	0,5	0	0
CC	10	2	49	1	0	0,5	0	0

Suplemmentary material

CC	10	2	51	1	0	0,5	0	0
CC	10	2	53	1	0	0,5	0	0
CC	10	2	55	0	0	0	0	0
CC	1	4	3	4	22	1	22	66
CC	1	4	5	4	48,75	1	48,75	243,75
CC	1	4	7	4	40,25	1	40,25	281,75
CC	1	4	9	4	51,75	1	51,75	465,75
CC	1	4	11	4	42,5	1	42,5	467,5
CC	1	4	13	4	38	1	38	494
CC	1	4	15	4	39,5	1	39,5	592,5
CC	1	4	17	4	44,75	1	44,75	760,75
CC	1	4	19	4	37,25	1	37,25	707,75
CC	1	4	21	4	41,75	1	41,75	876,75
CC	1	4	23	3	46,66666667	0,75	35	805
CC	1	4	25	3	32,66666667	0,75	24,5	612,5
CC	1	4	27	2	30,5	0,5	15,25	411,75
CC	1	4	29	2	10	0,5	5	145
CC	1	4	31	2	16,5	0,5	8,25	255,75
CC	1	4	33	2	35	0,5	17,5	577,5
CC	1	4	35	2	28	0,5	14	490
CC	1	4	37	2	19	0,5	9,5	351,5
CC	1	4	39	2	18	0,5	9	351
CC	1	4	41	2	10	0,5	5	205
CC	1	4	43	2	9,5	0,5	4,75	204,25
CC	1	4	45	2	0	0,5	0	0
CC	1	4	47	1	0	0,25	0	0
CC	1	4	49	1	0	0,25	0	0
CC	1	4	51	0	0	0	0	0
CC	2	4	3	4	12,5	1	12,5	37,5
CC	2	4	5	4	34,25	1	34,25	171,25
CC	2	4	7	4	34,5	1	34,5	241,5
CC	2	4	9	4	27,25	1	27,25	245,25
CC	2	4	11	4	26,25	1	26,25	288,75
CC	2	4	13	4	21	1	21	273
CC	2	4	15	4	24,25	1	24,25	363,75
CC	2	4	17	4	23,75	1	23,75	403,75
CC	2	4	19	4	13,5	1	13,5	256,5
CC	2	4	21	4	21,25	1	21,25	446,25
CC	2	4	23	4	21,75	1	21,75	500,25
CC	2	4	25	4	12	1	12	300
CC	2	4	27	3	3,333333333	0,75	2,5	67,5
CC	2	4	29	1	0	0,25	0	0
CC	2	4	31	1	0	0,25	0	0
CC	2	4	33	1	0	0,25	0	0
CC	2	4	35	1	0	0,25	0	0

Suplemmentary material

CC	2	4	37	1	0	0,25	0	0
CC	2	4	39	1	0	0,25	0	0
CC	2	4	41	1	0	0,25	0	0
CC	2	4	43	1	0	0,25	0	0
CC	2	4	45	1	0	0,25	0	0
CC	2	4	47	1	0	0,25	0	0
CC	2	4	49	1	0	0,25	0	0
CC	2	4	51	0	0	0	0	0
CC	3	4	3	4	29,75	1	29,75	89,25
CC	3	4	5	4	79,25	1	79,25	396,25
CC	3	4	7	4	40,75	1	40,75	285,25
CC	3	4	9	4	41,25	1	41,25	371,25
CC	3	4	11	4	31	1	31	341
CC	3	4	13	4	28	1	28	364
CC	3	4	15	4	18,75	1	18,75	281,25
CC	3	4	17	4	25	1	25	425
CC	3	4	19	4	31	1	31	589
CC	3	4	21	4	33,25	1	33,25	698,25
CC	3	4	23	4	21	1	21	483
CC	3	4	25	4	18	1	18	450
CC	3	4	27	4	25,75	1	25,75	695,25
CC	3	4	29	4	6	1	6	174
CC	3	4	31	3	12,33333333	0,75	9,25	286,75
CC	3	4	33	3	10	0,75	7,5	247,5
CC	3	4	35	3	4	0,75	3	105
CC	3	4	37	3	10,66666667	0,75	8	296
CC	3	4	39	3	0	0,75	0	0
CC	3	4	41	3	0	0,75	0	0
CC	3	4	43	2	0	0,5	0	0
CC	3	4	45	2	0	0,5	0	0
CC	3	4	47	2	0	0,5	0	0
CC	3	4	49	2	0	0,5	0	0
CC	3	4	51	1	0	0,25	0	0
CC	3	4	53	1	0	0,25	0	0
CC	3	4	55	1	0	0,25	0	0
CC	3	4	57	0	0	0	0	0
CC	5	4	3	4	16,75	1	16,75	50,25
CC	5	4	5	3	53,66666667	0,75	40,25	201,25
CC	5	4	7	3	40,33333333	0,75	30,25	211,75
CC	5	4	9	3	40	0,75	30	270
CC	5	4	11	3	38,33333333	0,75	28,75	316,25
CC	5	4	13	3	25,33333333	0,75	19	247
CC	5	4	15	3	26,33333333	0,75	19,75	296,25
CC	5	4	17	3	30,66666667	0,75	23	391
CC	5	4	19	3	16,66666667	0,75	12,5	237,5

Suplemmentary material

CC	5	4	21	3	20,33333333	0,75	15,25	320,25
CC	5	4	23	3	17,33333333	0,75	13	299
CC	5	4	25	3	23,66666667	0,75	17,75	443,75
CC	5	4	27	3	17	0,75	12,75	344,25
CC	5	4	29	3	13,66666667	0,75	10,25	297,25
CC	5	4	31	3	21	0,75	15,75	488,25
CC	5	4	33	3	17,66666667	0,75	13,25	437,25
CC	5	4	35	2	18	0,5	9	315
CC	5	4	37	2	16,5	0,5	8,25	305,25
CC	5	4	39	1	0	0,25	0	0
CC	5	4	41	1	2	0,25	0,5	20,5
CC	5	4	43	1	1	0,25	0,25	10,75
CC	5	4	45	1	0	0,25	0	0
CC	5	4	47	1	0	0,25	0	0
CC	5	4	49	1	0	0,25	0	0
CC	5	4	51	0	0	0	0	0
CC	6	4	3	4	15,5	1	15,5	46,5
CC	6	4	5	4	27	1	27	135
CC	6	4	7	4	58,25	1	58,25	407,75
CC	6	4	9	4	18,5	1	18,5	166,5
CC	6	4	11	4	33,25	1	33,25	365,75
CC	6	4	13	4	18,75	1	18,75	243,75
CC	6	4	15	4	22,5	1	22,5	337,5
CC	6	4	17	4	27,75	1	27,75	471,75
CC	6	4	19	4	25	1	25	475
CC	6	4	21	4	11,25	1	11,25	236,25
CC	6	4	23	4	10,5	1	10,5	241,5
CC	6	4	25	4	11,25	1	11,25	281,25
CC	6	4	27	4	24	1	24	648
CC	6	4	29	3	8	0,75	6	174
CC	6	4	31	2	26,5	0,5	13,25	410,75
CC	6	4	33	2	21,5	0,5	10,75	354,75
CC	6	4	35	1	13	0,25	3,25	113,75
CC	6	4	37	1	14	0,25	3,5	129,5
CC	6	4	39	1	0	0,25	0	0
CC	6	4	41	1	0	0,25	0	0
CC	6	4	43	0	0	0	0	0
CC	7	4	3	4	16,5	1	16,5	49,5
CC	7	4	5	4	27,25	1	27,25	136,25
CC	7	4	7	3	46,66666667	0,75	35	245
CC	7	4	9	3	44,33333333	0,75	33,25	299,25
CC	7	4	11	3	21,33333333	0,75	16	176
CC	7	4	13	2	43,5	0,5	21,75	282,75
CC	7	4	15	2	7	0,5	3,5	52,5
CC	7	4	17	2	9	0,5	4,5	76,5

Suplemmentary material

CC	7	4	19	2	30,5	0,5	15,25	289,75
CC	7	4	21	2	24	0,5	12	252
CC	7	4	23	2	17,5	0,5	8,75	201,25
CC	7	4	25	2	14	0,5	7	175
CC	7	4	27	2	14,5	0,5	7,25	195,75
CC	7	4	29	2	7	0,5	3,5	101,5
CC	7	4	31	1	20	0,25	5	155
CC	7	4	33	1	5	0,25	1,25	41,25
CC	7	4	35	1	5	0,25	1,25	43,75
CC	7	4	37	1	8	0,25	2	74
CC	7	4	39	1	1	0,25	0,25	9,75
CC	7	4	41	1	0	0,25	0	0
CC	7	4	43	0	0	0	0	0
CC	8	4	3	4	19,75	1	19,75	59,25
CC	8	4	5	4	30	1	30	150
CC	8	4	7	4	25	1	25	175
CC	8	4	9	4	21,25	1	21,25	191,25
CC	8	4	11	4	25,25	1	25,25	277,75
CC	8	4	13	4	22,5	1	22,5	292,5
CC	8	4	15	4	21,25	1	21,25	318,75
CC	8	4	17	4	23,25	1	23,25	395,25
CC	8	4	19	4	24,5	1	24,5	465,5
CC	8	4	21	3	40	0,75	30	630
CC	8	4	23	3	45	0,75	33,75	776,25
CC	8	4	25	3	52,33333333	0,75	39,25	981,25
CC	8	4	27	3	42	0,75	31,5	850,5
CC	8	4	29	3	35,33333333	0,75	26,5	768,5
CC	8	4	31	3	43,33333333	0,75	32,5	1007,5
CC	8	4	33	3	28,66666667	0,75	21,5	709,5
CC	8	4	35	3	32,33333333	0,75	24,25	848,75
CC	8	4	37	3	20,66666667	0,75	15,5	573,5
CC	8	4	39	3	6,666666667	0,75	5	195
CC	8	4	41	2	8	0,5	4	164
CC	8	4	43	1	0	0,25	0	0
CC	8	4	45	0	0	0	0	0
CC	9	4	3	4	22,5	1	22,5	67,5
CC	9	4	5	4	30,25	1	30,25	151,25
CC	9	4	7	4	29,5	1	29,5	206,5
CC	9	4	9	4	31,75	1	31,75	285,75
CC	9	4	11	4	43,5	1	43,5	478,5
CC	9	4	13	4	22	1	22	286
CC	9	4	15	4	26,25	1	26,25	393,75
CC	9	4	17	4	16,75	1	16,75	284,75
CC	9	4	19	4	13	1	13	247
CC	9	4	21	4	7,75	1	7,75	162,75

Suplemmentary material

CC	9	4	23	3	5,666666667	0,75	4,25	97,75
CC	9	4	25	3	8	0,75	6	150
CC	9	4	27	3	2,666666667	0,75	2	54
CC	9	4	29	3	0	0,75	0	0
CC	9	4	31	2	0	0,5	0	0
CC	9	4	33	2	0	0,5	0	0
CC	9	4	35	2	0	0,5	0	0
CC	9	4	37	1	0	0,25	0	0
CC	9	4	39	1	0	0,25	0	0
CC	9	4	41	1	0	0,25	0	0
CC	9	4	43	1	0	0,25	0	0
CC	9	4	45	1	0	0,25	0	0
CC	9	4	47	0	0	0	0	0
CC	10	4	3	4	18	1	18	54
CC	10	4	5	4	26	1	26	130
CC	10	4	7	4	53	1	53	371
CC	10	4	9	3	43,66666667	0,75	32,75	294,75
CC	10	4	11	3	41,66666667	0,75	31,25	343,75
CC	10	4	13	3	22	0,75	16,5	214,5
CC	10	4	15	3	47	0,75	35,25	528,75
CC	10	4	17	3	33	0,75	24,75	420,75
CC	10	4	19	3	34	0,75	25,5	484,5
CC	10	4	21	3	22,66666667	0,75	17	357
CC	10	4	23	3	24,33333333	0,75	18,25	419,75
CC	10	4	25	3	14	0,75	10,5	262,5
CC	10	4	27	3	16,66666667	0,75	12,5	337,5
CC	10	4	29	3	0	0,75	0	0
CC	10	4	31	3	2,333333333	0,75	1,75	54,25
CC	10	4	33	3	0	0,75	0	0
CC	10	4	35	3	2	0,75	1,5	52,5
CC	10	4	37	3	1,333333333	0,75	1	37
CC	10	4	39	2	0,5	0,5	0,25	9,75
CC	10	4	41	0	0	0	0	0
CC	1	8	3	8	12	1	12	36
CC	1	8	5	8	30,125	1	30,125	150,625
CC	1	8	7	8	32,5	1	32,5	227,5
CC	1	8	9	8	29,75	1	29,75	267,75
CC	1	8	11	8	37	1	37	407
CC	1	8	13	8	35,375	1	35,375	459,875
CC	1	8	15	8	15,625	1	15,625	234,375
CC	1	8	17	8	21,125	1	21,125	359,125
CC	1	8	19	8	22,375	1	22,375	425,125
CC	1	8	21	8	28,5	1	28,5	598,5
CC	1	8	23	8	16,75	1	16,75	385,25
CC	1	8	25	8	26,125	1	26,125	653,125

Suplemmentary material

CC	1	8	27	8	12,875	1	12,875	347,625
CC	1	8	29	8	17,25	1	17,25	500,25
CC	1	8	31	7	15,28571429	0,875	13,375	414,625
CC	1	8	33	7	8,857142857	0,875	7,75	255,75
CC	1	8	35	7	15,71428571	0,875	13,75	481,25
CC	1	8	37	7	7,714285714	0,875	6,75	249,75
CC	1	8	39	7	6,428571429	0,875	5,625	219,375
CC	1	8	41	7	0	0,875	0	0
CC	1	8	43	4	1,25	0,5	0,625	26,875
CC	1	8	45	2	0	0,25	0	0
CC	1	8	47	0	0	0	0	0
CC	2	8	3	8	8	1	8	24
CC	2	8	5	8	28,125	1	28,125	140,625
CC	2	8	7	8	16,75	1	16,75	117,25
CC	2	8	9	8	28,125	1	28,125	253,125
CC	2	8	11	8	19,125	1	19,125	210,375
CC	2	8	13	7	29,85714286	0,875	26,125	339,625
CC	2	8	15	7	14,71428571	0,875	12,875	193,125
CC	2	8	17	7	21,42857143	0,875	18,75	318,75
CC	2	8	19	7	14,14285714	0,875	12,375	235,125
CC	2	8	21	7	19,71428571	0,875	17,25	362,25
CC	2	8	23	6	19	0,75	14,25	327,75
CC	2	8	25	5	10,8	0,625	6,75	168,75
CC	2	8	27	5	7,8	0,625	4,875	131,625
CC	2	8	29	5	7,8	0,625	4,875	141,375
CC	2	8	31	5	10,6	0,625	6,625	205,375
CC	2	8	33	5	12,4	0,625	7,75	255,75
CC	2	8	35	5	4,6	0,625	2,875	100,625
CC	2	8	37	5	1,8	0,625	1,125	41,625
CC	2	8	39	5	3,2	0,625	2	78
CC	2	8	41	4	1,25	0,5	0,625	25,625
CC	2	8	43	4	0	0,5	0	0
CC	2	8	45	4	0	0,5	0	0
CC	2	8	47	2	0	0,25	0	0
CC	2	8	49	2	0	0,25	0	0
CC	2	8	51	1	0	0,125	0	0
CC	2	8	53	0	0	0	0	0
CC	3	8	3	8	35,75	1	35,75	107,25
CC	3	8	5	8	51	1	51	255
CC	3	8	7	8	37,25	1	37,25	260,75
CC	3	8	9	8	33,25	1	33,25	299,25
CC	3	8	11	8	31,875	1	31,875	350,625
CC	3	8	13	8	31,375	1	31,375	407,875
CC	3	8	15	8	33	1	33	495
CC	3	8	17	8	21,875	1	21,875	371,875

Suplemmentary material

CC	3	8	19	8	18,75	1	18,75	356,25
CC	3	8	21	8	17,125	1	17,125	359,625
CC	3	8	23	8	19,875	1	19,875	457,125
CC	3	8	25	8	24,25	1	24,25	606,25
CC	3	8	27	8	17,25	1	17,25	465,75
CC	3	8	29	6	15,5	0,75	11,625	337,125
CC	3	8	31	6	11,83333333	0,75	8,875	275,125
CC	3	8	33	6	7,666666667	0,75	5,75	189,75
CC	3	8	35	6	14,16666667	0,75	10,625	371,875
CC	3	8	37	6	8,166666667	0,75	6,125	226,625
CC	3	8	39	6	5,666666667	0,75	4,25	165,75
CC	3	8	41	6	2,166666667	0,75	1,625	66,625
CC	3	8	43	4	4,75	0,5	2,375	102,125
CC	3	8	45	1	15	0,125	1,875	84,375
CC	3	8	47	1	5	0,125	0,625	29,375
CC	3	8	49	1	2	0,125	0,25	12,25
CC	3	8	51	1	0	0,125	0	0
CC	3	8	53	1	0	0,125	0	0
CC	3	8	55	1	0	0,125	0	0
CC	3	8	57	1	0	0,125	0	0
CC	3	8	59	1	0	0,125	0	0
CC	3	8	61	1	0	0,125	0	0
CC	3	8	63	0	0	0	0	0
CC	4	8	3	8	35,875	1	35,875	107,625
CC	4	8	5	8	32,5	1	32,5	162,5
CC	4	8	7	7	36,28571429	0,875	31,75	222,25
CC	4	8	9	7	46,42857143	0,875	40,625	365,625
CC	4	8	11	7	31,28571429	0,875	27,375	301,125
CC	4	8	13	7	38,71428571	0,875	33,875	440,375
CC	4	8	15	7	34,57142857	0,875	30,25	453,75
CC	4	8	17	7	17,85714286	0,875	15,625	265,625
CC	4	8	19	7	28,57142857	0,875	25	475
CC	4	8	21	7	27,42857143	0,875	24	504
CC	4	8	23	7	25,85714286	0,875	22,625	520,375
CC	4	8	25	7	28,42857143	0,875	24,875	621,875
CC	4	8	27	7	13	0,875	11,375	307,125
CC	4	8	29	7	8,428571429	0,875	7,375	213,875
CC	4	8	31	7	2,571428571	0,875	2,25	69,75
CC	4	8	33	3	4,333333333	0,375	1,625	53,625
CC	4	8	35	2	8	0,25	2	70
CC	4	8	37	2	12,5	0,25	3,125	115,625
CC	4	8	39	2	6,5	0,25	1,625	63,375
CC	4	8	41	2	2	0,25	0,5	20,5
CC	4	8	43	2	2,5	0,25	0,625	26,875
CC	4	8	45	1	0	0,125	0	0

Suplemmentary material

CC	4	8	47	1	0	0,125	0	0
CC	4	8	49	0	0	0	0	0
CC	5	8	3	8	26,375	1	26,375	79,125
CC	5	8	5	8	18,5	1	18,5	92,5
CC	5	8	7	8	38,875	1	38,875	272,125
CC	5	8	9	8	29,375	1	29,375	264,375
CC	5	8	11	7	26,71428571	0,875	23,375	257,125
CC	5	8	13	7	24,57142857	0,875	21,5	279,5
CC	5	8	15	7	25,14285714	0,875	22	330
CC	5	8	17	7	14,28571429	0,875	12,5	212,5
CC	5	8	19	7	25,42857143	0,875	22,25	422,75
CC	5	8	21	7	13,85714286	0,875	12,125	254,625
CC	5	8	23	6	27,5	0,75	20,625	474,375
CC	5	8	25	6	18,16666667	0,75	13,625	340,625
CC	5	8	27	6	21,5	0,75	16,125	435,375
CC	5	8	29	6	25,83333333	0,75	19,375	561,875
CC	5	8	31	5	28,8	0,625	18	558
CC	5	8	33	5	33,6	0,625	21	693
CC	5	8	35	5	18	0,625	11,25	393,75
CC	5	8	37	4	25,5	0,5	12,75	471,75
CC	5	8	39	4	9	0,5	4,5	175,5
CC	5	8	41	4	4,75	0,5	2,375	97,375
CC	5	8	43	3	0	0,375	0	0
CC	5	8	45	2	9	0,25	2,25	101,25
CC	5	8	47	1	35	0,125	4,375	205,625
CC	5	8	49	1	20	0,125	2,5	122,5
CC	5	8	51	0	0	0	0	0
CC	6	8	3	8	34,875	1	34,875	104,625
CC	6	8	5	6	47,33333333	0,75	35,5	177,5
CC	6	8	7	6	66	0,75	49,5	346,5
CC	6	8	9	6	26,16666667	0,75	19,625	176,625
CC	6	8	11	6	45,83333333	0,75	34,375	378,125
CC	6	8	13	6	48	0,75	36	468
CC	6	8	15	6	52,33333333	0,75	39,25	588,75
CC	6	8	17	6	52,83333333	0,75	39,625	673,625
CC	6	8	19	6	44,33333333	0,75	33,25	631,75
CC	6	8	21	6	44,5	0,75	33,375	700,875
CC	6	8	23	6	26	0,75	19,5	448,5
CC	6	8	25	6	10,16666667	0,75	7,625	190,625
CC	6	8	27	6	10	0,75	7,5	202,5
CC	6	8	29	6	11,16666667	0,75	8,375	242,875
CC	6	8	31	6	10,33333333	0,75	7,75	240,25
CC	6	8	33	5	4,8	0,625	3	99
CC	6	8	35	5	4	0,625	2,5	87,5
CC	6	8	37	4	7,75	0,5	3,875	143,375

Suplemmentary material

CC	6	8	39	4	6,5	0,5	3,25	126,75
CC	6	8	41	3	2,666666667	0,375	1	41
CC	6	8	43	2	10	0,25	2,5	107,5
CC	6	8	45	2	18,5	0,25	4,625	208,125
CC	6	8	47	2	3	0,25	0,75	35,25
CC	6	8	49	2	2	0,25	0,5	24,5
CC	6	8	51	2	2,5	0,25	0,625	31,875
CC	6	8	53	2	0	0,25	0	0
CC	6	8	55	1	0	0,125	0	0
CC	6	8	57	0	0	0	0	0
CC	7	8	3	8	33	1	33	99
CC	7	8	5	8	25,875	1	25,875	129,375
CC	7	8	7	8	23,875	1	23,875	167,125
CC	7	8	9	8	35,625	1	35,625	320,625
CC	7	8	11	8	20,625	1	20,625	226,875
CC	7	8	13	8	32,75	1	32,75	425,75
CC	7	8	15	8	28,5	1	28,5	427,5
CC	7	8	17	8	17,625	1	17,625	299,625
CC	7	8	19	8	27,125	1	27,125	515,375
CC	7	8	21	8	25,25	1	25,25	530,25
CC	7	8	23	8	25,125	1	25,125	577,875
CC	7	8	25	8	17,25	1	17,25	431,25
CC	7	8	27	8	7,5	1	7,5	202,5
CC	7	8	29	8	21,75	1	21,75	630,75
CC	7	8	31	7	22,57142857	0,875	19,75	612,25
CC	7	8	33	7	9,142857143	0,875	8	264
CC	7	8	35	7	9	0,875	7,875	275,625
CC	7	8	37	7	4,428571429	0,875	3,875	143,375
CC	7	8	39	6	3,666666667	0,75	2,75	107,25
CC	7	8	41	6	19	0,75	14,25	584,25
CC	7	8	43	4	3	0,5	1,5	64,5
CC	7	8	45	2	0,5	0,25	0,125	5,625
CC	7	8	47	1	7	0,125	0,875	41,125
CC	7	8	49	0	0	0	0	0
CC	8	8	3	8	7,25	1	7,25	21,75
CC	8	8	5	6	28,83333333	0,75	21,625	108,125
CC	8	8	7	6	20	0,75	15	105
CC	8	8	9	6	16,33333333	0,75	12,25	110,25
CC	8	8	11	6	15,16666667	0,75	11,375	125,125
CC	8	8	13	6	27,83333333	0,75	20,875	271,375
CC	8	8	15	6	29,5	0,75	22,125	331,875
CC	8	8	17	6	19,66666667	0,75	14,75	250,75
CC	8	8	19	6	14,16666667	0,75	10,625	201,875
CC	8	8	21	5	10,8	0,625	6,75	141,75
CC	8	8	23	5	18,4	0,625	11,5	264,5

Suplemmentary material

CC	8	8	25	4	22,5	0,5	11,25	281,25
CC	8	8	27	4	16,75	0,5	8,375	226,125
CC	8	8	29	4	14,5	0,5	7,25	210,25
CC	8	8	31	4	4,5	0,5	2,25	69,75
CC	8	8	33	3	9	0,375	3,375	111,375
CC	8	8	35	3	7	0,375	2,625	91,875
CC	8	8	37	3	4,333333333	0,375	1,625	60,125
CC	8	8	39	3	6	0,375	2,25	87,75
CC	8	8	41	3	13	0,375	4,875	199,875
CC	8	8	43	2	4	0,25	1	43
CC	8	8	45	2	1	0,25	0,25	11,25
CC	8	8	47	2	1,5	0,25	0,375	17,625
CC	8	8	49	1	0	0,125	0	0
CC	8	8	51	1	0	0,125	0	0
CC	8	8	53	1	0	0,125	0	0
CC	8	8	55	0	0	0	0	0
CC	1	16	3	16	7,25	1	7,25	21,75
CC	1	16	5	16	30,3125	1	30,3125	151,5625
CC	1	16	7	16	29,6875	1	29,6875	207,8125
CC	1	16	9	16	29,25	1	29,25	263,25
CC	1	16	11	16	29,0625	1	29,0625	319,6875
CC	1	16	13	16	36,125	1	36,125	469,625
CC	1	16	15	16	34	1	34	510
CC	1	16	17	16	32,6875	1	32,6875	555,6875
CC	1	16	19	16	19,9375	1	19,9375	378,8125
CC	1	16	21	16	19,875	1	19,875	417,375
CC	1	16	23	16	15,1875	1	15,1875	349,3125
CC	1	16	25	16	17,0625	1	17,0625	426,5625
CC	1	16	27	15	15,73333333	0,9375	14,75	398,25
CC	1	16	29	15	8,133333333	0,9375	7,625	221,125
CC	1	16	31	13	8,461538462	0,8125	6,875	213,125
CC	1	16	33	11	4	0,6875	2,75	90,75
CC	1	16	35	11	9,454545455	0,6875	6,5	227,5
CC	1	16	37	11	7,818181818	0,6875	5,375	198,875
CC	1	16	39	9	2	0,5625	1,125	43,875
CC	1	16	41	7	5,428571429	0,4375	2,375	97,375
CC	1	16	43	7	2,571428571	0,4375	1,125	48,375
CC	1	16	45	6	1,666666667	0,375	0,625	28,125
CC	1	16	47	6	8,666666667	0,375	3,25	152,75
CC	1	16	49	4	4,75	0,25	1,1875	58,1875
CC	1	16	51	0	0	0	0	0
CC	2	16	3	16	3,1875	1	3,1875	9,5625
CC	2	16	5	13	19,23076923	0,8125	15,625	78,125
CC	2	16	7	13	25,38461538	0,8125	20,625	144,375
CC	2	16	9	12	26,66666667	0,75	20	180

Suplemmentary material

CC	2	16	11	10	21,1	0,625	13,1875	145,0625
CC	2	16	13	10	26,6	0,625	16,625	216,125
CC	2	16	15	10	24,9	0,625	15,5625	233,4375
CC	2	16	17	10	20,7	0,625	12,9375	219,9375
CC	2	16	19	10	22,3	0,625	13,9375	264,8125
CC	2	16	21	10	25,4	0,625	15,875	333,375
CC	2	16	23	10	17,9	0,625	11,1875	257,3125
CC	2	16	25	10	25,4	0,625	15,875	396,875
CC	2	16	27	10	14,6	0,625	9,125	246,375
CC	2	16	29	10	10,9	0,625	6,8125	197,5625
CC	2	16	31	9	13,66666667	0,5625	7,6875	238,3125
CC	2	16	33	8	13,25	0,5	6,625	218,625
CC	2	16	35	8	6,375	0,5	3,1875	111,5625
CC	2	16	37	7	5	0,4375	2,1875	80,9375
CC	2	16	39	7	2,857142857	0,4375	1,25	48,75
CC	2	16	41	7	2,714285714	0,4375	1,1875	48,6875
CC	2	16	43	6	0	0,375	0	0
CC	2	16	45	5	2	0,3125	0,625	28,125
CC	2	16	47	4	0	0,25	0	0
CC	2	16	49	3	0	0,1875	0	0
CC	2	16	51	2	0	0,125	0	0
CC	2	16	53	2	0	0,125	0	0
CC	2	16	55	1	0	0,0625	0	0
CC	2	16	57	0	0	0	0	0
CC	3	16	3	16	11,9375	1	11,9375	35,8125
CC	3	16	5	16	16,625	1	16,625	83,125
CC	3	16	7	16	19,8125	1	19,8125	138,6875
CC	3	16	9	16	18,75	1	18,75	168,75
CC	3	16	11	15	16	0,9375	15	165
CC	3	16	13	15	17,4	0,9375	16,3125	212,0625
CC	3	16	15	15	15,46666667	0,9375	14,5	217,5
CC	3	16	17	15	13,4	0,9375	12,5625	213,5625
CC	3	16	19	14	12,85714286	0,875	11,25	213,75
CC	3	16	21	14	12,07142857	0,875	10,5625	221,8125
CC	3	16	23	14	9,642857143	0,875	8,4375	194,0625
CC	3	16	25	14	8,357142857	0,875	7,3125	182,8125
CC	3	16	27	14	9,214285714	0,875	8,0625	217,6875
CC	3	16	29	14	11,57142857	0,875	10,125	293,625
CC	3	16	31	14	13,21428571	0,875	11,5625	358,4375
CC	3	16	33	14	11,5	0,875	10,0625	332,0625
CC	3	16	35	12	12,75	0,75	9,5625	334,6875
CC	3	16	37	11	13,36363636	0,6875	9,1875	339,9375
CC	3	16	39	11	9,090909091	0,6875	6,25	243,75
CC	3	16	41	8	8	0,5	4	164
CC	3	16	43	4	3	0,25	0,75	32,25

Suplemmentary material

CC	3	16	45	2	6	0,125	0,75	33,75
CC	3	16	47	2	0	0,125	0	0
CC	3	16	49	2	0	0,125	0	0
CC	3	16	51	2	0	0,125	0	0
CC	3	16	53	2	0	0,125	0	0
CC	3	16	55	0	0	0	0	0
CC	4	16	3	16	19,75	1	19,75	59,25
CC	4	16	5	16	23	1	23	115
CC	4	16	7	16	19,125	1	19,125	133,875
CC	4	16	9	16	15,6875	1	15,6875	141,1875
CC	4	16	11	16	19,875	1	19,875	218,625
CC	4	16	13	16	20,6875	1	20,6875	268,9375
CC	4	16	15	16	18,75	1	18,75	281,25
CC	4	16	17	16	17,4375	1	17,4375	296,4375
CC	4	16	19	16	19,5	1	19,5	370,5
CC	4	16	21	16	13,625	1	13,625	286,125
CC	4	16	23	16	11,125	1	11,125	255,875
CC	4	16	25	16	13,6875	1	13,6875	342,1875
CC	4	16	27	16	12,8125	1	12,8125	345,9375
CC	4	16	29	12	15	0,75	11,25	326,25
CC	4	16	31	11	14,18181818	0,6875	9,75	302,25
CC	4	16	33	11	15,54545455	0,6875	10,6875	352,6875
CC	4	16	35	11	13,72727273	0,6875	9,4375	330,3125
CC	4	16	37	11	13,36363636	0,6875	9,1875	339,9375
CC	4	16	39	10	13,1	0,625	8,1875	319,3125
CC	4	16	41	8	10,375	0,5	5,1875	212,6875
CC	4	16	43	6	5,166666667	0,375	1,9375	83,3125
CC	4	16	45	6	1,166666667	0,375	0,4375	19,6875
CC	4	16	47	6	1,166666667	0,375	0,4375	20,5625
CC	4	16	49	4	0	0,25	0	0
CC	4	16	51	2	0	0,125	0	0
CC	4	16	53	2	0	0,125	0	0
CC	4	16	55	0	0	0	0	0
CC	5	16	3	16	17,6875	1	17,6875	53,0625
CC	5	16	5	16	24,9375	1	24,9375	124,6875
CC	5	16	7	16	14,25	1	14,25	99,75
CC	5	16	9	16	20,25	1	20,25	182,25
CC	5	16	11	16	21,75	1	21,75	239,25
CC	5	16	13	16	10,125	1	10,125	131,625
CC	5	16	15	16	19,6875	1	19,6875	295,3125
CC	5	16	17	14	20,71428571	0,875	18,125	308,125
CC	5	16	19	13	21,61538462	0,8125	17,5625	333,6875
CC	5	16	21	12	23,58333333	0,75	17,6875	371,4375
CC	5	16	23	12	18,25	0,75	13,6875	314,8125
CC	5	16	25	12	28	0,75	21	525

Suplemmentary material

CC	5	16	27	12	22,83333333	0,75	17,125	462,375
CC	5	16	29	12	15,08333333	0,75	11,3125	328,0625
CC	5	16	31	10	20,5	0,625	12,8125	397,1875
CC	5	16	33	9	14,22222222	0,5625	8	264
CC	5	16	35	7	3,714285714	0,4375	1,625	56,875
CC	5	16	37	6	1,333333333	0,375	0,5	18,5
CC	5	16	39	4	5,5	0,25	1,375	53,625
CC	5	16	41	2	5	0,125	0,625	25,625
CC	5	16	43	2	21,5	0,125	2,6875	115,5625
CC	5	16	45	2	6	0,125	0,75	33,75
CC	5	16	47	2	12,5	0,125	1,5625	73,4375
CC	5	16	49	1	3	0,0625	0,1875	9,1875
CC	5	16	51	1	0	0,0625	0	0
CC	5	16	53	0	0	0	0	0
CC	6	16	3	16	2,125	1	2,125	6,375
CC	6	16	5	13	8,307692308	0,8125	6,75	33,75
CC	6	16	7	13	34,38461538	0,8125	27,9375	195,5625
CC	6	16	9	13	22,84615385	0,8125	18,5625	167,0625
CC	6	16	11	13	27,15384615	0,8125	22,0625	242,6875
CC	6	16	13	13	21,53846154	0,8125	17,5	227,5
CC	6	16	15	13	22,07692308	0,8125	17,9375	269,0625
CC	6	16	17	13	34,15384615	0,8125	27,75	471,75
CC	6	16	19	13	26,53846154	0,8125	21,5625	409,6875
CC	6	16	21	13	16,15384615	0,8125	13,125	275,625
CC	6	16	23	13	17,15384615	0,8125	13,9375	320,5625
CC	6	16	25	12	17,66666667	0,75	13,25	331,25
CC	6	16	27	11	15,45454545	0,6875	10,625	286,875
CC	6	16	29	11	16,18181818	0,6875	11,125	322,625
CC	6	16	31	11	6,727272727	0,6875	4,625	143,375
CC	6	16	33	9	12,44444444	0,5625	7	231
CC	6	16	35	8	8	0,5	4	140
CC	6	16	37	7	3	0,4375	1,3125	48,5625
CC	6	16	39	6	8,5	0,375	3,1875	124,3125
CC	6	16	41	6	3,166666667	0,375	1,1875	48,6875
CC	6	16	43	5	0,4	0,3125	0,125	5,375
CC	6	16	45	4	2,25	0,25	0,5625	25,3125
CC	6	16	47	3	0,333333333	0,1875	0,0625	2,9375
CC	6	16	49	1	0	0,0625	0	0
CC	6	16	51	0	0	0	0	0
CC	7	16	3	16	6,375	1	6,375	19,125
CC	7	16	5	14	14,35714286	0,875	12,5625	62,8125
CC	7	16	7	14	21,42857143	0,875	18,75	131,25
CC	7	16	9	14	12,42857143	0,875	10,875	97,875
CC	7	16	11	13	10,07692308	0,8125	8,1875	90,0625
CC	7	16	13	13	10,92307692	0,8125	8,875	115,375

Suplemmentary material

CC	7	16	15	13	18,07692308	0,8125	14,6875	220,3125
CC	7	16	17	13	13,92307692	0,8125	11,3125	192,3125
CC	7	16	19	13	6,153846154	0,8125	5	95
CC	7	16	21	13	3,153846154	0,8125	2,5625	53,8125
CC	7	16	23	13	6,230769231	0,8125	5,0625	116,4375
CC	7	16	25	13	0,538461538	0,8125	0,4375	10,9375
CC	7	16	27	12	4,416666667	0,75	3,3125	89,4375
CC	7	16	29	10	4,3	0,625	2,6875	77,9375
CC	7	16	31	10	1,5	0,625	0,9375	29,0625
CC	7	16	33	10	1,6	0,625	1	33
CC	7	16	35	10	0,2	0,625	0,125	4,375
CC	7	16	37	9	0	0,5625	0	0
CC	7	16	39	6	4,333333333	0,375	1,625	63,375
CC	7	16	41	4	0,25	0,25	0,0625	2,5625
CC	7	16	43	2	0,5	0,125	0,0625	2,6875
CC	7	16	45	1	0	0,0625	0	0
CC	7	16	47	1	0	0,0625	0	0
CC	7	16	39	0	0	0	0	0
CC	8	16	3	16	9,5625	1	9,5625	28,6875
CC	8	16	5	15	15,46666667	0,9375	14,5	72,5
CC	8	16	7	15	24,8	0,9375	23,25	162,75
CC	8	16	9	14	16,07142857	0,875	14,0625	126,5625
CC	8	16	11	14	10	0,875	8,75	96,25
CC	8	16	13	14	19,78571429	0,875	17,3125	225,0625
CC	8	16	15	14	17,28571429	0,875	15,125	226,875
CC	8	16	17	14	15,5	0,875	13,5625	230,5625
CC	8	16	19	14	6,285714286	0,875	5,5	104,5
CC	8	16	21	14	11,35714286	0,875	9,9375	208,6875
CC	8	16	23	14	13,71428571	0,875	12	276
CC	8	16	25	12	7	0,75	5,25	131,25
CC	8	16	27	12	3,25	0,75	2,4375	65,8125
CC	8	16	29	12	1,833333333	0,75	1,375	39,875
CC	8	16	31	9	9,777777778	0,5625	5,5	170,5
CC	8	16	33	9	5	0,5625	2,8125	92,8125
CC	8	16	35	7	7,285714286	0,4375	3,1875	111,5625
CC	8	16	37	7	6	0,4375	2,625	97,125
CC	8	16	39	4	1,5	0,25	0,375	14,625
CC	8	16	41	4	1	0,25	0,25	10,25
CC	8	16	43	3	1	0,1875	0,1875	8,0625
CC	8	16	45	3	0	0,1875	0	0
CC	8	16	47	3	0	0,1875	0	0
CC	8	16	49	0	0	0	0	0
CV	2	2	3	2	1	1	1	3
CV	2	2	5	2	3,5	1	3,5	17,5
CV	2	2	7	2	58	1	58	406

Suplemmentary material

CV	2	2	9	2	50,5	1	50,5	454,5
CV	2	2	11	2	30	1	30	330
CV	2	2	13	2	49	1	49	637
CV	2	2	15	2	23,5	1	23,5	352,5
CV	2	2	17	2	23	1	23	391
CV	2	2	19	2	38,5	1	38,5	731,5
CV	2	2	21	2	41	1	41	861
CV	2	2	23	2	33	1	33	759
CV	2	2	25	2	25	1	25	625
CV	2	2	27	2	22	1	22	594
CV	2	2	29	2	10	1	10	290
CV	2	2	31	2	11	1	11	341
CV	2	2	33	2	15,5	1	15,5	511,5
CV	2	2	35	2	28,5	1	28,5	997,5
CV	2	2	37	2	3,5	1	3,5	129,5
CV	2	2	39	2	2	1	2	78
CV	2	2	41	1	0	0,5	0	0
CV	2	2	43	1	0	0,5	0	0
CV	2	2	45	1	0	0,5	0	0
CV	2	2	47	1	0	0,5	0	0
CV	2	2	49	0	0	0	0	0
CV	3	2	3	2	51,5	1	51,5	154,5
CV	3	2	5	2	64,5	1	64,5	322,5
CV	3	2	7	2	84,5	1	84,5	591,5
CV	3	2	9	2	88	1	88	792
CV	3	2	11	2	46	1	46	506
CV	3	2	13	2	53	1	53	689
CV	3	2	15	2	65,5	1	65,5	982,5
CV	3	2	17	2	38,5	1	38,5	654,5
CV	3	2	19	2	48	1	48	912
CV	3	2	21	2	48,5	1	48,5	1018,5
CV	3	2	23	1	33	0,5	16,5	379,5
CV	3	2	25	1	52	0,5	26	650
CV	3	2	27	1	20	0,5	10	270
CV	3	2	29	1	12	0,5	6	174
CV	3	2	31	1	17	0,5	8,5	263,5
CV	3	2	33	1	0	0,5	0	0
CV	3	2	35	1	0	0,5	0	0
CV	3	2	37	1	0	0,5	0	0
CV	3	2	39	1	0	0,5	0	0
CV	3	2	41	1	0	0,5	0	0
CV	3	2	43	1	0	0,5	0	0
CV	3	2	45	1	0	0,5	0	0
CV	3	2	47	1	0	0,5	0	0
CV	3	2	49	1	0	0,5	0	0

Suplemmentary material

CV	3	2	51	1	0	0,5	0	0
CV	3	2	53	0	0	0	0	0
CV	4	2	3	2	1,5	1	1,5	4,5
CV	4	2	5	2	1,5	1	1,5	7,5
CV	4	2	7	2	17,5	1	17,5	122,5
CV	4	2	9	2	19,5	1	19,5	175,5
CV	4	2	11	2	9,5	1	9,5	104,5
CV	4	2	13	2	2,5	1	2,5	32,5
CV	4	2	15	2	2	1	2	30
CV	4	2	17	2	10,5	1	10,5	178,5
CV	4	2	19	2	3,5	1	3,5	66,5
CV	4	2	21	2	7,5	1	7,5	157,5
CV	4	2	23	2	17	1	17	391
CV	4	2	25	2	2,5	1	2,5	62,5
CV	4	2	27	2	1,5	1	1,5	40,5
CV	4	2	29	2	1,5	1	1,5	43,5
CV	4	2	31	2	4	1	4	124
CV	4	2	33	2	2	1	2	66
CV	4	2	35	2	0,5	1	0,5	17,5
CV	4	2	37	2	1	1	1	37
CV	4	2	39	1	0	0,5	0	0
CV	4	2	41	0	0	0	0	0
CV	5	2	3	2	16	1	16	48
CV	5	2	5	2	77,5	1	77,5	387,5
CV	5	2	7	2	53,5	1	53,5	374,5
CV	5	2	9	2	74	1	74	666
CV	5	2	11	2	49	1	49	539
CV	5	2	13	2	43	1	43	559
CV	5	2	15	2	31	1	31	465
CV	5	2	17	2	36	1	36	612
CV	5	2	19	2	48,5	1	48,5	921,5
CV	5	2	21	2	35,5	1	35,5	745,5
CV	5	2	23	2	41,5	1	41,5	954,5
CV	5	2	25	2	23,5	1	23,5	587,5
CV	5	2	27	2	50,5	1	50,5	1363,5
CV	5	2	29	2	40	1	40	1160
CV	5	2	31	2	35,5	1	35,5	1100,5
CV	5	2	33	2	30	1	30	990
CV	5	2	35	2	15	1	15	525
CV	5	2	37	2	16	1	16	592
CV	5	2	39	2	1	1	1	39
CV	5	2	41	1	6	0,5	3	123
CV	5	2	43	1	8	0,5	4	172
CV	5	2	45	1	2	0,5	1	45
CV	5	2	47	1	0	0,5	0	0

Suplemmentary material

CV	5	2	49	0	0	0	0	0
CV	6	2	3	2	34	1	34	102
CV	6	2	5	2	39	1	39	195
CV	6	2	7	2	36	1	36	252
CV	6	2	9	2	40,5	1	40,5	364,5
CV	6	2	11	2	46,5	1	46,5	511,5
CV	6	2	13	2	29,5	1	29,5	383,5
CV	6	2	15	2	43,5	1	43,5	652,5
CV	6	2	17	2	19,5	1	19,5	331,5
CV	6	2	19	2	48	1	48	912
CV	6	2	21	2	45	1	45	945
CV	6	2	23	2	21	1	21	483
CV	6	2	25	2	31,5	1	31,5	787,5
CV	6	2	27	2	12,5	1	12,5	337,5
CV	6	2	29	2	8,5	1	8,5	246,5
CV	6	2	31	2	0	1	0	0
CV	6	2	33	2	6	1	6	198
CV	6	2	35	2	10	1	10	350
CV	6	2	37	2	10,5	1	10,5	388,5
CV	6	2	39	2	0	1	0	0
CV	6	2	41	1	12	0,5	6	246
CV	6	2	43	1	0	0,5	0	0
CV	6	2	45	1	0	0,5	0	0
CV	6	2	47	1	0	0,5	0	0
CV	6	2	49	1	0	0,5	0	0
CV	6	2	51	0	0	0	0	0
CV	7	2	3	2	1,5	1	1,5	4,5
CV	7	2	5	2	18	1	18	90
CV	7	2	7	2	57	1	57	399
CV	7	2	9	2	58,5	1	58,5	526,5
CV	7	2	11	2	1,5	1	1,5	16,5
CV	7	2	13	2	20	1	20	260
CV	7	2	15	2	63	1	63	945
CV	7	2	17	2	24,5	1	24,5	416,5
CV	7	2	19	2	36,5	1	36,5	693,5
CV	7	2	21	2	20	1	20	420
CV	7	2	23	2	20,5	1	20,5	471,5
CV	7	2	25	1	10	0,5	5	125
CV	7	2	27	1	0	0,5	0	0
CV	7	2	29	1	1	0,5	0,5	14,5
CV	7	2	31	1	2	0,5	1	31
CV	7	2	33	1	4	0,5	2	66
CV	7	2	35	1	9	0,5	4,5	157,5
CV	7	2	37	1	2	0,5	1	37
CV	7	2	39	1	0	0,5	0	0

Suplemmentary material

CV	7	2	41	1	0	0,5	0	0
CV	7	2	43	0	0	0	0	0
CV	8	2	3	2	10,5	1	10,5	31,5
CV	8	2	5	2	39	1	39	195
CV	8	2	7	2	42,5	1	42,5	297,5
CV	8	2	9	2	5,5	1	5,5	49,5
CV	8	2	11	2	38	1	38	418
CV	8	2	13	2	46,5	1	46,5	604,5
CV	8	2	15	2	39	1	39	585
CV	8	2	17	2	43	1	43	731
CV	8	2	19	2	59	1	59	1121
CV	8	2	21	2	20,5	1	20,5	430,5
CV	8	2	23	1	48	0,5	24	552
CV	8	2	25	1	71	0,5	35,5	887,5
CV	8	2	27	1	49	0,5	24,5	661,5
CV	8	2	29	1	24	0,5	12	348
CV	8	2	31	1	17	0,5	8,5	263,5
CV	8	2	33	1	47	0,5	23,5	775,5
CV	8	2	35	1	54	0,5	27	945
CV	8	2	37	1	21	0,5	10,5	388,5
CV	8	2	39	1	0	0,5	0	0
CV	8	2	41	1	3	0,5	1,5	61,5
CV	8	2	43	0	0	0	0	0
CV	1	4	3	4	0,5	1	0,5	1,5
CV	1	4	5	4	46,25	1	46,25	231,25
CV	1	4	7	4	61,25	1	61,25	428,75
CV	1	4	9	4	42,25	1	42,25	380,25
CV	1	4	11	4	45,25	1	45,25	497,75
CV	1	4	13	4	34,5	1	34,5	448,5
CV	1	4	15	4	27,75	1	27,75	416,25
CV	1	4	17	4	38,25	1	38,25	650,25
CV	1	4	19	4	36	1	36	684
CV	1	4	21	4	40,5	1	40,5	850,5
CV	1	4	23	4	18,75	1	18,75	431,25
CV	1	4	25	4	24	1	24	600
CV	1	4	27	4	14,75	1	14,75	398,25
CV	1	4	29	4	7	1	7	203
CV	1	4	31	4	5,5	1	5,5	170,5
CV	1	4	33	4	0,75	1	0,75	24,75
CV	1	4	35	4	2,25	1	2,25	78,75
CV	1	4	37	3	7	0,75	5,25	194,25
CV	1	4	39	2	5,5	0,5	2,75	107,25
CV	1	4	41	2	6	0,5	3	123
CV	1	4	43	2	1,5	0,5	0,75	32,25
CV	1	4	45	1	0	0,25	0	0

Suplemmentary material

CV	1	4	47	1	2	0,25	0,5	23,5
CV	1	4	49	1	0	0,25	0	0
CV	1	4	51	1	2	0,25	0,5	25,5
CV	1	4	53	0	0	0	0	0
CV	2	4	3	4	2,25	1	2,25	6,75
CV	2	4	5	4	26,25	1	26,25	131,25
CV	2	4	7	4	35,25	1	35,25	246,75
CV	2	4	9	4	44,25	1	44,25	398,25
CV	2	4	11	4	24,5	1	24,5	269,5
CV	2	4	13	4	46	1	46	598
CV	2	4	15	3	60	0,75	45	675
CV	2	4	17	3	75,66666667	0,75	56,75	964,75
CV	2	4	19	3	46,33333333	0,75	34,75	660,25
CV	2	4	21	3	59,66666667	0,75	44,75	939,75
CV	2	4	23	3	39,33333333	0,75	29,5	678,5
CV	2	4	25	3	30	0,75	22,5	562,5
CV	2	4	27	3	22	0,75	16,5	445,5
CV	2	4	29	3	17	0,75	12,75	369,75
CV	2	4	31	3	9,666666667	0,75	7,25	224,75
CV	2	4	33	3	5,333333333	0,75	4	132
CV	2	4	35	2	12,5	0,5	6,25	218,75
CV	2	4	37	2	4,5	0,5	2,25	83,25
CV	2	4	39	2	0	0,5	0	0
CV	2	4	41	1	0	0,25	0	0
CV	2	4	43	1	0	0,25	0	0
CV	2	4	45	0	0	0	0	0
CV	3	4	3	4	15	1	15	45
CV	3	4	5	4	46	1	46	230
CV	3	4	7	3	46,33333333	0,75	34,75	243,25
CV	3	4	9	3	74	0,75	55,5	499,5
CV	3	4	11	3	42,33333333	0,75	31,75	349,25
CV	3	4	13	3	22,33333333	0,75	16,75	217,75
CV	3	4	15	3	29,33333333	0,75	22	330
CV	3	4	17	3	19,66666667	0,75	14,75	250,75
CV	3	4	19	3	24,33333333	0,75	18,25	346,75
CV	3	4	21	3	18,33333333	0,75	13,75	288,75
CV	3	4	23	3	7,666666667	0,75	5,75	132,25
CV	3	4	25	3	6,333333333	0,75	4,75	118,75
CV	3	4	27	1	8	0,25	2	54
CV	3	4	29	1	11	0,25	2,75	79,75
CV	3	4	31	1	5	0,25	1,25	38,75
CV	3	4	33	1	0	0,25	0	0
CV	3	4	35	1	0	0,25	0	0
CV	3	4	37	0	0	0	0	0
CV	4	4	3	4	27,5	1	27,5	82,5

Suplemmentary material

CV	4	4	5	4	48,75	1	48,75	243,75
CV	4	4	7	4	34,75	1	34,75	243,25
CV	4	4	9	4	37	1	37	333
CV	4	4	11	4	34,25	1	34,25	376,75
CV	4	4	13	4	10,75	1	10,75	139,75
CV	4	4	15	3	26,66666667	0,75	20	300
CV	4	4	17	3	12,66666667	0,75	9,5	161,5
CV	4	4	19	3	24	0,75	18	342
CV	4	4	21	3	30	0,75	22,5	472,5
CV	4	4	23	3	16,66666667	0,75	12,5	287,5
CV	4	4	25	3	16,33333333	0,75	12,25	306,25
CV	4	4	27	3	9,666666667	0,75	7,25	195,75
CV	4	4	29	3	10,33333333	0,75	7,75	224,75
CV	4	4	31	3	5	0,75	3,75	116,25
CV	4	4	33	2	2,5	0,5	1,25	41,25
CV	4	4	35	1	1	0,25	0,25	8,75
CV	4	4	37	1	0	0,25	0	0
CV	4	4	39	1	0	0,25	0	0
CV	4	4	41	1	0	0,25	0	0
CV	4	4	43	0	0	0	0	0
CV	6	4	3	4	0	1	0	0
CV	6	4	5	4	35	1	35	175
CV	6	4	7	4	34	1	34	238
CV	6	4	9	4	18,75	1	18,75	168,75
CV	6	4	11	4	32	1	32	352
CV	6	4	13	4	27,25	1	27,25	354,25
CV	6	4	15	4	21,75	1	21,75	326,25
CV	6	4	17	4	17,75	1	17,75	301,75
CV	6	4	19	4	12,75	1	12,75	242,25
CV	6	4	21	3	23,66666667	0,75	17,75	372,75
CV	6	4	23	3	11,66666667	0,75	8,75	201,25
CV	6	4	25	3	23	0,75	17,25	431,25
CV	6	4	27	3	10,66666667	0,75	8	216
CV	6	4	29	2	20	0,5	10	290
CV	6	4	31	2	8,5	0,5	4,25	131,75
CV	6	4	33	2	1,5	0,5	0,75	24,75
CV	6	4	35	2	0	0,5	0	0
CV	6	4	37	1	1	0,25	0,25	9,25
CV	6	4	39	0	0	0	0	0
CV	8	4	3	4	6,75	1	6,75	20,25
CV	8	4	5	4	27,75	1	27,75	138,75
CV	8	4	7	4	36,75	1	36,75	257,25
CV	8	4	9	4	46,5	1	46,5	418,5
CV	8	4	11	4	33,25	1	33,25	365,75
CV	8	4	13	4	48,75	1	48,75	633,75

Suplemmentary material

CV	8	4	15	4	28	1	28	420
CV	8	4	17	3	12,33333333	0,75	9,25	157,25
CV	8	4	19	3	27,33333333	0,75	20,5	389,5
CV	8	4	21	3	21,33333333	0,75	16	336
CV	8	4	23	3	16	0,75	12	276
CV	8	4	25	3	17	0,75	12,75	318,75
CV	8	4	27	3	17,66666667	0,75	13,25	357,75
CV	8	4	29	3	9,333333333	0,75	7	203
CV	8	4	31	3	3	0,75	2,25	69,75
CV	8	4	33	3	0,333333333	0,75	0,25	8,25
CV	8	4	35	3	0	0,75	0	0
CV	8	4	37	2	0	0,5	0	0
CV	8	4	39	1	0	0,25	0	0
CV	8	4	41	1	0	0,25	0	0
CV	8	4	43	0	0	0	0	0
CV	1	8	3	8	4,875	1	4,875	14,625
CV	1	8	5	8	40,875	1	40,875	204,375
CV	1	8	7	8	33,75	1	33,75	236,25
CV	1	8	9	8	36,25	1	36,25	326,25
CV	1	8	11	8	38,5	1	38,5	423,5
CV	1	8	13	8	22	1	22	286
CV	1	8	15	8	36,5	1	36,5	547,5
CV	1	8	17	8	29,5	1	29,5	501,5
CV	1	8	19	8	41,25	1	41,25	783,75
CV	1	8	21	8	36,25	1	36,25	761,25
CV	1	8	23	8	28,25	1	28,25	649,75
CV	1	8	25	8	22,75	1	22,75	568,75
CV	1	8	27	7	29,28571429	0,875	25,625	691,875
CV	1	8	29	7	29,71428571	0,875	26	754
CV	1	8	31	7	9,571428571	0,875	8,375	259,625
CV	1	8	33	6	12	0,75	9	297
CV	1	8	35	6	12	0,75	9	315
CV	1	8	37	6	7,166666667	0,75	5,375	198,875
CV	1	8	39	6	6,5	0,75	4,875	190,125
CV	1	8	41	6	2	0,75	1,5	61,5
CV	1	8	43	4	0	0,5	0	0
CV	1	8	45	2	0	0,25	0	0
CV	1	8	47	1	0	0,125	0	0
CV	1	8	49	1	0	0,125	0	0
CV	1	8	51	1	0	0,125	0	0
CV	1	8	53	1	0	0,125	0	0
CV	1	8	55	1	0	0,125	0	0
CV	1	8	57	1	0	0,125	0	0
CV	1	8	59	0	0	0	0	0
CV	2	8	3	8	9,5	1	9,5	28,5

Suplemmentary material

CV	2	8	5	8	25,75	1	25,75	128,75
CV	2	8	7	8	18,125	1	18,125	126,875
CV	2	8	9	8	35,375	1	35,375	318,375
CV	2	8	11	8	24,25	1	24,25	266,75
CV	2	8	13	8	16,625	1	16,625	216,125
CV	2	8	15	8	22,375	1	22,375	335,625
CV	2	8	17	8	22	1	22	374
CV	2	8	19	8	9,25	1	9,25	175,75
CV	2	8	21	8	10,125	1	10,125	212,625
CV	2	8	23	7	8	0,875	7	161
CV	2	8	25	7	1	0,875	0,875	21,875
CV	2	8	27	6	1	0,75	0,75	20,25
CV	2	8	29	6	4,333333333	0,75	3,25	94,25
CV	2	8	31	6	0,5	0,75	0,375	11,625
CV	2	8	33	6	0	0,75	0	0
CV	2	8	35	5	0,6	0,625	0,375	13,125
CV	2	8	37	5	0,6	0,625	0,375	13,875
CV	2	8	39	5	0	0,625	0	0
CV	2	8	41	4	0	0,5	0	0
CV	2	8	43	4	0	0,5	0	0
CV	2	8	45	4	0	0,5	0	0
CV	2	8	47	1	0	0,125	0	0
CV	2	8	49	0	0	0	0	0
CV	3	8	3	4	18,75	0,5	9,375	28,125
CV	3	8	5	4	16,5	0,5	8,25	41,25
CV	3	8	7	4	12,75	0,5	6,375	44,625
CV	3	8	9	4	17,25	0,5	8,625	77,625
CV	3	8	11	3	19,33333333	0,375	7,25	79,75
CV	3	8	13	2	28,5	0,25	7,125	92,625
CV	3	8	15	2	14	0,25	3,5	52,5
CV	3	8	17	2	16	0,25	4	68
CV	3	8	19	1	14	0,125	1,75	33,25
CV	3	8	21	0	0	0	0	0
CV	4	8	3	8	6,75	1	6,75	20,25
CV	4	8	5	8	36,625	1	36,625	183,125
CV	4	8	7	8	31	1	31	217
CV	4	8	9	8	34,875	1	34,875	313,875
CV	4	8	11	8	38,5	1	38,5	423,5
CV	4	8	13	8	30,375	1	30,375	394,875
CV	4	8	15	8	24,625	1	24,625	369,375
CV	4	8	17	8	19,25	1	19,25	327,25
CV	4	8	19	8	24	1	24	456
CV	4	8	21	8	19	1	19	399
CV	4	8	23	8	15,625	1	15,625	359,375
CV	4	8	25	6	13,33333333	0,75	10	250

Suplemmentary material

CV	4	8	27	5	22	0,625	13,75	371,25
CV	4	8	29	5	16,8	0,625	10,5	304,5
CV	4	8	31	5	26,2	0,625	16,375	507,625
CV	4	8	33	4	18,75	0,5	9,375	309,375
CV	4	8	35	4	17	0,5	8,5	297,5
CV	4	8	37	4	19	0,5	9,5	351,5
CV	4	8	39	4	14,5	0,5	7,25	282,75
CV	4	8	41	4	11	0,5	5,5	225,5
CV	4	8	43	4	6	0,5	3	129
CV	4	8	45	4	3,75	0,5	1,875	84,375
CV	4	8	47	1	4	0,125	0,5	23,5
CV	4	8	49	0	0	0	0	0
CV	5	8	3	8	1	1	1	3
CV	5	8	5	8	17,25	1	17,25	86,25
CV	5	8	7	8	23,5	1	23,5	164,5
CV	5	8	9	8	14,5	1	14,5	130,5
CV	5	8	11	8	17,875	1	17,875	196,625
CV	5	8	13	7	16,71428571	0,875	14,625	190,125
CV	5	8	15	7	15,14285714	0,875	13,25	198,75
CV	5	8	17	7	19	0,875	16,625	282,625
CV	5	8	19	7	7,142857143	0,875	6,25	118,75
CV	5	8	21	7	6,714285714	0,875	5,875	123,375
CV	5	8	23	6	13,66666667	0,75	10,25	235,75
CV	5	8	25	6	18,66666667	0,75	14	350
CV	5	8	27	6	15,33333333	0,75	11,5	310,5
CV	5	8	29	5	15	0,625	9,375	271,875
CV	5	8	31	5	15,6	0,625	9,75	302,25
CV	5	8	33	5	20,2	0,625	12,625	416,625
CV	5	8	35	4	12,5	0,5	6,25	218,75
CV	5	8	37	3	15,66666667	0,375	5,875	217,375
CV	5	8	39	3	3,666666667	0,375	1,375	53,625
CV	5	8	41	2	0	0,25	0	0
CV	5	8	43	2	0	0,25	0	0
CV	5	8	45	1	0	0,125	0	0
CV	5	8	47	1	0	0,125	0	0
CV	5	8	49	1	0	0,125	0	0
CV	5	8	51	1	0	0,125	0	0
CV	5	8	53	1	0	0,125	0	0
CV	5	8	55	1	0	0,125	0	0
CV	5	8	57	1	0	0,125	0	0
CV	5	8	59	0	0	0	0	0
CV	6	8	3	8	0,25	1	0,25	0,75
CV	6	8	5	7	28,85714286	0,875	25,25	126,25
CV	6	8	7	7	41,14285714	0,875	36	252
CV	6	8	9	6	28,33333333	0,75	21,25	191,25

Suplemmentary material

CV	6	8	11	6	39,66666667	0,75	29,75	327,25
CV	6	8	13	6	26,66666667	0,75	20	260
CV	6	8	15	6	17,33333333	0,75	13	195
CV	6	8	17	6	31,33333333	0,75	23,5	399,5
CV	6	8	19	6	47	0,75	35,25	669,75
CV	6	8	21	6	15,5	0,75	11,625	244,125
CV	6	8	23	6	25,33333333	0,75	19	437
CV	6	8	25	6	22,83333333	0,75	17,125	428,125
CV	6	8	27	6	20,16666667	0,75	15,125	408,375
CV	6	8	29	6	31,66666667	0,75	23,75	688,75
CV	6	8	31	6	23,33333333	0,75	17,5	542,5
CV	6	8	33	6	8	0,75	6	198
CV	6	8	35	5	3,6	0,625	2,25	78,75
CV	6	8	37	5	1,8	0,625	1,125	41,625
CV	6	8	39	5	1,4	0,625	0,875	34,125
CV	6	8	41	2	0	0,25	0	0
CV	6	8	43	2	0	0,25	0	0
CV	6	8	45	2	0	0,25	0	0
CV	6	8	47	1	0	0,125	0	0
CV	6	8	49	1	0	0,125	0	0
CV	6	8	51	1	0	0,125	0	0
CV	6	8	53	1	0	0,125	0	0
CV	6	8	55	1	0	0,125	0	0
CV	6	8	57	0	0	0	0	0
CV	7	8	3	8	0,125	1	0,125	0,375
CV	7	8	5	8	19	1	19	95
CV	7	8	7	7	16,71428571	0,875	14,625	102,375
CV	7	8	9	6	13,66666667	0,75	10,25	92,25
CV	7	8	11	6	14,66666667	0,75	11	121
CV	7	8	13	6	31,33333333	0,75	23,5	305,5
CV	7	8	15	6	25,5	0,75	19,125	286,875
CV	7	8	17	6	17,66666667	0,75	13,25	225,25
CV	7	8	19	6	20,5	0,75	15,375	292,125
CV	7	8	21	5	9	0,625	5,625	118,125
CV	7	8	23	5	21,6	0,625	13,5	310,5
CV	7	8	25	5	20,8	0,625	13	325
CV	7	8	27	5	10,6	0,625	6,625	178,875
CV	7	8	29	5	23,2	0,625	14,5	420,5
CV	7	8	31	4	15,25	0,5	7,625	236,375
CV	7	8	33	4	16,5	0,5	8,25	272,25
CV	7	8	35	4	15,25	0,5	7,625	266,875
CV	7	8	37	4	5,25	0,5	2,625	97,125
CV	7	8	39	4	12,5	0,5	6,25	243,75
CV	7	8	41	4	5,5	0,5	2,75	112,75
CV	7	8	43	4	6,75	0,5	3,375	145,125

Suplemmentary material

CV	7	8	45	4	0,5	0,5	0,25	11,25
CV	7	8	47	1	0	0,125	0	0
CV	7	8	49	1	2	0,125	0,25	12,25
CV	7	8	51	0	0	0	0	0
CV	8	8	3	8	0,375	1	0,375	1,125
CV	8	8	5	8	18,125	1	18,125	90,625
CV	8	8	7	7	37,28571429	0,875	32,625	228,375
CV	8	8	9	5	46,2	0,625	28,875	259,875
CV	8	8	11	5	40,6	0,625	25,375	279,125
CV	8	8	13	5	25,4	0,625	15,875	206,375
CV	8	8	15	4	37	0,5	18,5	277,5
CV	8	8	17	4	35,75	0,5	17,875	303,875
CV	8	8	19	4	50	0,5	25	475
CV	8	8	21	4	26,25	0,5	13,125	275,625
CV	8	8	23	4	41,75	0,5	20,875	480,125
CV	8	8	25	4	15,25	0,5	7,625	190,625
CV	8	8	27	4	30	0,5	15	405
CV	8	8	29	3	17,66666667	0,375	6,625	192,125
CV	8	8	31	3	18	0,375	6,75	209,25
CV	8	8	33	3	16,33333333	0,375	6,125	202,125
CV	8	8	35	3	2	0,375	0,75	26,25
CV	8	8	37	3	0,666666667	0,375	0,25	9,25
CV	8	8	39	1	0	0,125	0	0
CV	8	8	41	1	3	0,125	0,375	15,375
CV	8	8	43	1	4	0,125	0,5	21,5
CV	8	8	45	1	0	0,125	0	0
CV	8	8	47	1	0	0,125	0	0
CV	8	8	49	1	0	0,125	0	0
CV	8	8	51	0	0	0	0	0
CV	1	16	3	16	5	1	5	15
CV	1	16	5	15	16,26666667	0,9375	15,25	76,25
CV	1	16	7	12	42,83333333	0,75	32,125	224,875
CV	1	16	9	12	37,83333333	0,75	28,375	255,375
CV	1	16	11	12	21,25	0,75	15,9375	175,3125
CV	1	16	13	11	34,54545455	0,6875	23,75	308,75
CV	1	16	15	11	21,54545455	0,6875	14,8125	222,1875
CV	1	16	17	11	31,72727273	0,6875	21,8125	370,8125
CV	1	16	19	11	37,81818182	0,6875	26	494
CV	1	16	21	11	27	0,6875	18,5625	389,8125
CV	1	16	23	10	16,8	0,625	10,5	241,5
CV	1	16	25	10	16,3	0,625	10,1875	254,6875
CV	1	16	27	10	14,7	0,625	9,1875	248,0625
CV	1	16	29	10	13,6	0,625	8,5	246,5
CV	1	16	31	9	6	0,5625	3,375	104,625
CV	1	16	33	9	6,333333333	0,5625	3,5625	117,5625

Suplemmentary material

CV	1	16	35	9	6,555555556	0,5625	3,6875	129,0625
CV	1	16	37	7	4,285714286	0,4375	1,875	69,375
CV	1	16	39	4	10	0,25	2,5	97,5
CV	1	16	41	2	16,5	0,125	2,0625	84,5625
CV	1	16	43	2	9,5	0,125	1,1875	51,0625
CV	1	16	45	2	0	0,125	0	0
CV	1	16	47	2	8,5	0,125	1,0625	49,9375
CV	1	16	49	2	2	0,125	0,25	12,25
CV	1	16	51	2	0	0,125	0	0
CV	1	16	53	2	0	0,125	0	0
CV	1	16	55	1	0	0,0625	0	0
CV	1	16	57	0	0	0	0	0
CV	2	16	3	16	0,25	1	0,25	0,75
CV	2	16	5	13	8,538461538	0,8125	6,9375	34,6875
CV	2	16	7	13	16,61538462	0,8125	13,5	94,5
CV	2	16	9	10	21,1	0,625	13,1875	118,6875
CV	2	16	11	10	23,1	0,625	14,4375	158,8125
CV	2	16	13	10	22,2	0,625	13,875	180,375
CV	2	16	15	10	22,4	0,625	14	210
CV	2	16	17	10	35,8	0,625	22,375	380,375
CV	2	16	19	10	17,4	0,625	10,875	206,625
CV	2	16	21	10	26,1	0,625	16,3125	342,5625
CV	2	16	23	10	13,8	0,625	8,625	198,375
CV	2	16	25	10	11,8	0,625	7,375	184,375
CV	2	16	27	10	9	0,625	5,625	151,875
CV	2	16	29	10	9	0,625	5,625	163,125
CV	2	16	31	10	6,6	0,625	4,125	127,875
CV	2	16	33	10	1,4	0,625	0,875	28,875
CV	2	16	35	10	0,7	0,625	0,4375	15,3125
CV	2	16	37	8	0,5	0,5	0,25	9,25
CV	2	16	39	4	1	0,25	0,25	9,75
CV	2	16	41	4	0	0,25	0	0
CV	2	16	43	4	4,25	0,25	1,0625	45,6875
CV	2	16	45	1	0	0,0625	0	0
CV	2	16	47	1	0	0,0625	0	0
CV	2	16	49	1	8	0,0625	0,5	24,5
CV	2	16	51	1	8	0,0625	0,5	25,5
CV	2	16	53	0	0	0	0	0
CV	3	16	3	16	14,125	1	14,125	42,375
CV	3	16	5	14	17,57142857	0,875	15,375	76,875
CV	3	16	7	14	21,78571429	0,875	19,0625	133,4375
CV	3	16	9	14	22,07142857	0,875	19,3125	173,8125
CV	3	16	11	14	17,85714286	0,875	15,625	171,875
CV	3	16	13	14	16,78571429	0,875	14,6875	190,9375
CV	3	16	15	12	18,08333333	0,75	13,5625	203,4375

Suplemmentary material

CV	3	16	17	12	24,25	0,75	18,1875	309,1875
CV	3	16	19	12	18	0,75	13,5	256,5
CV	3	16	21	12	18,5	0,75	13,875	291,375
CV	3	16	23	12	16,25	0,75	12,1875	280,3125
CV	3	16	25	11	10,45454545	0,6875	7,1875	179,6875
CV	3	16	27	11	11,90909091	0,6875	8,1875	221,0625
CV	3	16	29	10	11,6	0,625	7,25	210,25
CV	3	16	31	9	7,666666667	0,5625	4,3125	133,6875
CV	3	16	33	9	3,111111111	0,5625	1,75	57,75
CV	3	16	35	7	0,714285714	0,4375	0,3125	10,9375
CV	3	16	37	4	1,75	0,25	0,4375	16,1875
CV	3	16	39	4	0,75	0,25	0,1875	7,3125
CV	3	16	41	2	0	0,125	0	0
CV	3	16	43	2	0,5	0,125	0,0625	2,6875
CV	3	16	45	2	1	0,125	0,125	5,625
CV	3	16	47	2	0,5	0,125	0,0625	2,9375
CV	3	16	49	2	2	0,125	0,25	12,25
CV	3	16	51	2	0	0,125	0	0
CV	3	16	53	0	0	0	0	0
CV	4	16	3	16	14,1875	1	14,1875	42,5625
CV	4	16	5	16	12,625	1	12,625	63,125
CV	4	16	7	16	20,5	1	20,5	143,5
CV	4	16	9	16	22,1875	1	22,1875	199,6875
CV	4	16	11	15	25,46666667	0,9375	23,875	262,625
CV	4	16	13	15	24,73333333	0,9375	23,1875	301,4375
CV	4	16	15	15	27,46666667	0,9375	25,75	386,25
CV	4	16	17	15	27	0,9375	25,3125	430,3125
CV	4	16	19	15	21,53333333	0,9375	20,1875	383,5625
CV	4	16	21	11	25,54545455	0,6875	17,5625	368,8125
CV	4	16	23	11	23,54545455	0,6875	16,1875	372,3125
CV	4	16	25	10	9,5	0,625	5,9375	148,4375
CV	4	16	27	10	11,5	0,625	7,1875	194,0625
CV	4	16	29	10	10,9	0,625	6,8125	197,5625
CV	4	16	31	9	8,555555556	0,5625	4,8125	149,1875
CV	4	16	33	9	10,11111111	0,5625	5,6875	187,6875
CV	4	16	35	8	6,625	0,5	3,3125	115,9375
CV	4	16	37	5	5,4	0,3125	1,6875	62,4375
CV	4	16	39	5	3,8	0,3125	1,1875	46,3125
CV	4	16	41	5	0,8	0,3125	0,25	10,25
CV	4	16	43	5	0,4	0,3125	0,125	5,375
CV	4	16	45	5	0,4	0,3125	0,125	5,625
CV	4	16	47	2	3	0,125	0,375	17,625
CV	4	16	49	2	3,5	0,125	0,4375	21,4375
CV	4	16	51	2	2	0,125	0,25	12,75
CV	4	16	53	1	0	0,0625	0	0

Suplemmentary material

CV	4	16	55	0	0	0	0	0
CV	5	16	3	16	5,625	1	5,625	16,875
CV	5	16	5	16	9,75	1	9,75	48,75
CV	5	16	7	15	23,2	0,9375	21,75	152,25
CV	5	16	9	15	19,6	0,9375	18,375	165,375
CV	5	16	11	14	22,71428571	0,875	19,875	218,625
CV	5	16	13	14	22,5	0,875	19,6875	255,9375
CV	5	16	15	14	14,14285714	0,875	12,375	185,625
CV	5	16	17	14	13,78571429	0,875	12,0625	205,0625
CV	5	16	19	13	23,69230769	0,8125	19,25	365,75
CV	5	16	21	13	16,69230769	0,8125	13,5625	284,8125
CV	5	16	23	13	9,230769231	0,8125	7,5	172,5
CV	5	16	25	13	14,69230769	0,8125	11,9375	298,4375
CV	5	16	27	13	12,15384615	0,8125	9,875	266,625
CV	5	16	29	13	11,76923077	0,8125	9,5625	277,3125
CV	5	16	31	13	16,38461538	0,8125	13,3125	412,6875
CV	5	16	33	11	5,545454545	0,6875	3,8125	125,8125
CV	5	16	35	11	5,181818182	0,6875	3,5625	124,6875
CV	5	16	37	11	4,727272727	0,6875	3,25	120,25
CV	5	16	39	8	0	0,5	0	0
CV	5	16	41	7	1	0,4375	0,4375	17,9375
CV	5	16	43	7	0	0,4375	0	0
CV	5	16	45	7	0	0,4375	0	0
CV	5	16	47	5	0	0,3125	0	0
CV	5	16	49	3	0	0,1875	0	0
CV	5	16	51	0	0	0	0	0
CV	6	16	3	16	7,4375	1	7,4375	22,3125
CV	6	16	5	16	24,0625	1	24,0625	120,3125
CV	6	16	7	15	19	0,9375	17,8125	124,6875
CV	6	16	9	15	27,66666667	0,9375	25,9375	233,4375
CV	6	16	11	15	20,73333333	0,9375	19,4375	213,8125
CV	6	16	13	15	23	0,9375	21,5625	280,3125
CV	6	16	15	15	8,066666667	0,9375	7,5625	113,4375
CV	6	16	17	15	19,4	0,9375	18,1875	309,1875
CV	6	16	19	15	16,73333333	0,9375	15,6875	298,0625
CV	6	16	21	15	16,46666667	0,9375	15,4375	324,1875
CV	6	16	23	15	11,33333333	0,9375	10,625	244,375
CV	6	16	25	15	3,666666667	0,9375	3,4375	85,9375
CV	6	16	27	14	9,214285714	0,875	8,0625	217,6875
CV	6	16	29	13	10,92307692	0,8125	8,875	257,375
CV	6	16	31	13	9,153846154	0,8125	7,4375	230,5625
CV	6	16	33	11	13,90909091	0,6875	9,5625	315,5625
CV	6	16	35	11	0	0,6875	0	0
CV	6	16	37	10	0,9	0,625	0,5625	20,8125
CV	6	16	39	9	0	0,5625	0	0

Suplemmentary material

CV	6	16	41	6	0	0,375	0	0
CV	6	16	43	5	1,4	0,3125	0,4375	18,8125
CV	6	16	45	4	0	0,25	0	0
CV	6	16	47	2	0	0,125	0	0
CV	6	16	49	2	0	0,125	0	0
CV	6	16	51	1	0	0,0625	0	0
CV	6	16	53	0	0	0	0	0
CV	7	16	3	16	4,25	1	4,25	12,75
CV	7	16	5	16	15,3125	1	15,3125	76,5625
CV	7	16	7	16	18,375	1	18,375	128,625
CV	7	16	9	16	13,75	1	13,75	123,75
CV	7	16	11	16	14,6875	1	14,6875	161,5625
CV	7	16	13	16	16,25	1	16,25	211,25
CV	7	16	15	16	17,125	1	17,125	256,875
CV	7	16	17	16	15,3125	1	15,3125	260,3125
CV	7	16	19	16	18,9375	1	18,9375	359,8125
CV	7	16	21	16	20,6875	1	20,6875	434,4375
CV	7	16	23	15	21,26666667	0,9375	19,9375	458,5625
CV	7	16	25	12	17,75	0,75	13,3125	332,8125
CV	7	16	27	12	13	0,75	9,75	263,25
CV	7	16	29	12	14,33333333	0,75	10,75	311,75
CV	7	16	31	12	18,16666667	0,75	13,625	422,375
CV	7	16	33	12	5,25	0,75	3,9375	129,9375
CV	7	16	35	12	6,333333333	0,75	4,75	166,25
CV	7	16	37	6	9,833333333	0,375	3,6875	136,4375
CV	7	16	39	5	6,2	0,3125	1,9375	75,5625
CV	7	16	41	5	3,6	0,3125	1,125	46,125
CV	7	16	43	5	0	0,3125	0	0
CV	7	16	45	2	0	0,125	0	0
CV	7	16	47	0	0	0	0	0
CV	8	16	3	16	5,9375	1	5,9375	17,8125
CV	8	16	5	16	14,625	1	14,625	73,125
CV	8	16	7	16	11,125	1	11,125	77,875
CV	8	16	9	15	18,8	0,9375	17,625	158,625
CV	8	16	11	15	19,06666667	0,9375	17,875	196,625
CV	8	16	13	15	14,8	0,9375	13,875	180,375
CV	8	16	15	15	25,06666667	0,9375	23,5	352,5
CV	8	16	17	15	15,46666667	0,9375	14,5	246,5
CV	8	16	19	15	14,06666667	0,9375	13,1875	250,5625
CV	8	16	21	14	17,92857143	0,875	15,6875	329,4375
CV	8	16	23	14	10,92857143	0,875	9,5625	219,9375
CV	8	16	25	13	17,69230769	0,8125	14,375	359,375
CV	8	16	27	13	14	0,8125	11,375	307,125
CV	8	16	29	13	17	0,8125	13,8125	400,5625
CV	8	16	31	13	17,23076923	0,8125	14	434

Suplemmentary material

CV	8	16	33	13	10,92307692	0,8125	8,875	292,875
CV	8	16	35	13	7,769230769	0,8125	6,3125	220,9375
CV	8	16	37	12	7,916666667	0,75	5,9375	219,6875
CV	8	16	39	12	4	0,75	3	117
CV	8	16	41	12	1,583333333	0,75	1,1875	48,6875
CV	8	16	43	12	0,166666667	0,75	0,125	5,375
CV	8	16	45	7	0,571428571	0,4375	0,25	11,25
CV	8	16	47	3	0,666666667	0,1875	0,125	5,875
CV	8	16	49	3	1,333333333	0,1875	0,25	12,25
CV	8	16	51	1	0	0,0625	0	0
CV	8	16	53	0	0	0	0	0
VV	1	2	3	2	1	1	1	3
VV	1	2	5	2	43,5	1	43,5	217,5
VV	1	2	7	2	20,5	1	20,5	143,5
VV	1	2	9	2	25	1	25	225
VV	1	2	11	2	27	1	27	297
VV	1	2	13	1	11	0,5	5,5	71,5
VV	1	2	15	1	0	0,5	0	0
VV	1	2	17	1	0	0,5	0	0
VV	1	2	19	1	2	0,5	1	19
VV	1	2	21	1	0	0,5	0	0
VV	1	2	23	1	0	0,5	0	0
VV	1	2	25	1	0	0,5	0	0
VV	1	2	27	1	0	0,5	0	0
VV	1	2	29	0	0	0	0	0
VV	2	2	3	2	0	1	0	0
VV	2	2	5	2	20,5	1	20,5	102,5
VV	2	2	7	2	47,5	1	47,5	332,5
VV	2	2	9	2	44,5	1	44,5	400,5
VV	2	2	11	2	67	1	67	737
VV	2	2	13	2	52	1	52	676
VV	2	2	15	2	45	1	45	675
VV	2	2	17	2	35,5	1	35,5	603,5
VV	2	2	19	2	41	1	41	779
VV	2	2	21	2	31	1	31	651
VV	2	2	23	2	42,5	1	42,5	977,5
VV	2	2	25	2	35	1	35	875
VV	2	2	27	2	30	1	30	810
VV	2	2	29	2	12,5	1	12,5	362,5
VV	2	2	31	2	19	1	19	589
VV	2	2	33	1	37	0,5	18,5	610,5
VV	2	2	44	0	0	0	0	0
VV	3	2	3	2	1,5	1	1,5	4,5
VV	3	2	5	2	43,5	1	43,5	217,5
VV	3	2	7	2	48	1	48	336

Suplemmentary material

VV	3	2	9	2	32	1	32	288
VV	3	2	11	2	43,5	1	43,5	478,5
VV	3	2	13	2	38	1	38	494
VV	3	2	15	2	41	1	41	615
VV	3	2	17	2	30,5	1	30,5	518,5
VV	3	2	19	1	84	0,5	42	798
VV	3	2	21	1	28	0,5	14	294
VV	3	2	23	1	39	0,5	19,5	448,5
VV	3	2	25	1	21	0,5	10,5	262,5
VV	3	2	27	1	8	0,5	4	108
VV	3	2	29	0	0	0	0	0
VV	4	2	3	2	2,5	1	2,5	7,5
VV	4	2	5	2	43	1	43	215
VV	4	2	7	2	61	1	61	427
VV	4	2	9	2	44	1	44	396
VV	4	2	11	2	46	1	46	506
VV	4	2	13	2	40,5	1	40,5	526,5
VV	4	2	15	2	31,5	1	31,5	472,5
VV	4	2	17	2	23,5	1	23,5	399,5
VV	4	2	19	2	25	1	25	475
VV	4	2	21	1	27	0,5	13,5	283,5
VV	4	2	23	1	5	0,5	2,5	57,5
VV	4	2	25	1	0	0,5	0	0
VV	4	2	27	1	0	0,5	0	0
VV	4	2	29	1	0	0,5	0	0
VV	4	2	31	1	0	0,5	0	0
VV	4	2	33	0	0	0	0	0
VV	5	2	3	2	5,5	1	5,5	16,5
VV	5	2	5	2	39	1	39	195
VV	5	2	7	2	37	1	37	259
VV	5	2	9	2	46,5	1	46,5	418,5
VV	5	2	11	2	16,5	1	16,5	181,5
VV	5	2	13	2	35	1	35	455
VV	5	2	15	2	31	1	31	465
VV	5	2	17	2	21,5	1	21,5	365,5
VV	5	2	19	2	15,5	1	15,5	294,5
VV	5	2	21	2	22	1	22	462
VV	5	2	23	2	33	1	33	759
VV	5	2	25	2	30	1	30	750
VV	5	2	27	2	17	1	17	459
VV	5	2	29	2	1,5	1	1,5	43,5
VV	5	2	31	1	0	0,5	0	0
VV	5	2	33	1	0	0,5	0	0
VV	5	2	35	1	0	0,5	0	0
VV	5	2	37	0	0	0	0	0

Suplemmentary material

VV	6	2	3	2	0	1	0	0
VV	6	2	5	1	0	0,5	0	0
VV	6	2	7	1	4	0,5	2	14
VV	6	2	9	1	0	0,5	0	0
VV	6	2	11	1	0	0,5	0	0
VV	6	2	13	1	1	0,5	0,5	6,5
VV	6	2	15	1	1	0,5	0,5	7,5
VV	6	2	17	1	0	0,5	0	0
VV	6	2	19	1	0	0,5	0	0
VV	6	2	21	0	0	0	0	0
VV	7	2	3	2	3,5	1	3,5	10,5
VV	7	2	5	2	0,5	1	0,5	2,5
VV	7	2	7	2	23	1	23	161
VV	7	2	9	2	36	1	36	324
VV	7	2	11	2	26,5	1	26,5	291,5
VV	7	2	13	2	29	1	29	377
VV	7	2	15	2	35	1	35	525
VV	7	2	17	2	21	1	21	357
VV	7	2	19	2	34	1	34	646
VV	7	2	21	2	35	1	35	735
VV	7	2	23	2	26,5	1	26,5	609,5
VV	7	2	25	2	25,5	1	25,5	637,5
VV	7	2	27	2	6,5	1	6,5	175,5
VV	7	2	29	1	0	0,5	0	0
VV	7	2	31	0	0	0	0	0
VV	1	4	3	4	5,5	1	5,5	16,5
VV	1	4	5	4	21,75	1	21,75	108,75
VV	1	4	7	4	37,5	1	37,5	262,5
VV	1	4	9	4	28,5	1	28,5	256,5
VV	1	4	11	4	29,5	1	29,5	324,5
VV	1	4	13	4	25	1	25	325
VV	1	4	15	3	31	0,75	23,25	348,75
VV	1	4	17	3	31	0,75	23,25	395,25
VV	1	4	19	2	38,5	0,5	19,25	365,75
VV	1	4	21	2	22	0,5	11	231
VV	1	4	23	2	23	0,5	11,5	264,5
VV	1	4	25	2	20,5	0,5	10,25	256,25
VV	1	4	27	2	1,5	0,5	0,75	20,25
VV	1	4	29	0	0	0	0	0
VV	2	4	3	4	6	1	6	18
VV	2	4	5	4	24,5	1	24,5	122,5
VV	2	4	7	4	42,75	1	42,75	299,25
VV	2	4	9	3	27,33333333	0,75	20,5	184,5
VV	2	4	11	3	27	0,75	20,25	222,75
VV	2	4	13	3	36	0,75	27	351

Suplemmentary material

VV	2	4	15	3	27	0,75	20,25	303,75
VV	2	4	17	2	41,5	0,5	20,75	352,75
VV	2	4	19	2	21	0,5	10,5	199,5
VV	2	4	21	2	6	0,5	3	63
VV	2	4	23	2	2	0,5	1	23
VV	2	4	25	1	1	0,25	0,25	6,25
VV	2	4	27	1	0	0,25	0	0
VV	2	4	29	1	0	0,25	0	0
VV	2	4	31	1	0	0,25	0	0
VV	2	4	33	0	0	0	0	0
VV	3	4	3	4	1,75	1	1,75	5,25
VV	3	4	5	4	4,75	1	4,75	23,75
VV	3	4	7	4	12	1	12	84
VV	3	4	9	4	15,25	1	15,25	137,25
VV	3	4	11	4	14,75	1	14,75	162,25
VV	3	4	13	3	16,33333333	0,75	12,25	159,25
VV	3	4	15	3	8,333333333	0,75	6,25	93,75
VV	3	4	17	3	10,66666667	0,75	8	136
VV	3	4	19	3	17,66666667	0,75	13,25	251,75
VV	3	4	21	2	47,5	0,5	23,75	498,75
VV	3	4	23	2	27,5	0,5	13,75	316,25
VV	3	4	25	2	6	0,5	3	75
VV	3	4	27	2	2,5	0,5	1,25	33,75
VV	3	4	29	2	3	0,5	1,5	43,5
VV	3	4	31	2	5,5	0,5	2,75	85,25
VV	3	4	33	2	4	0,5	2	66
VV	3	4	35	2	9,5	0,5	4,75	166,25
VV	3	4	37	2	11	0,5	5,5	203,5
VV	3	4	39	2	14	0,5	7	273
VV	3	4	41	2	18,5	0,5	9,25	379,25
VV	3	4	43	2	22	0,5	11	473
VV	3	4	45	2	6,5	0,5	3,25	146,25
VV	3	4	47	1	25	0,25	6,25	293,75
VV	3	4	49	1	12	0,25	3	147
VV	3	4	51	1	2	0,25	0,5	25,5
VV	3	4	53	1	8	0,25	2	106
VV	3	4	55	0	0	0	0	0
VV	4	4	3	4	0,25	1	0,25	0,75
VV	4	4	5	4	5,25	1	5,25	26,25
VV	4	4	7	4	13,25	1	13,25	92,75
VV	4	4	9	4	18,75	1	18,75	168,75
VV	4	4	11	4	23,5	1	23,5	258,5
VV	4	4	13	4	24,75	1	24,75	321,75
VV	4	4	15	4	17,75	1	17,75	266,25
VV	4	4	17	4	16,5	1	16,5	280,5

Suplemmentary material

VV	4	4	19	4	16,75	1	16,75	318,25
VV	4	4	21	4	10	1	10	210
VV	4	4	23	4	6,25	1	6,25	143,75
VV	4	4	25	4	0,75	1	0,75	18,75
VV	4	4	27	4	2	1	2	54
VV	4	4	29	4	0,75	1	0,75	21,75
VV	4	4	31	3	5	0,75	3,75	116,25
VV	4	4	33	3	1,333333333	0,75	1	33
VV	4	4	35	3	0,666666667	0,75	0,5	17,5
VV	4	4	37	2	0	0,5	0	0
VV	4	4	39	2	0	0,5	0	0
VV	4	4	41	2	0	0,5	0	0
VV	4	4	43	0	0	0	0	0
VV	5	4	3	4	14,75	1	14,75	44,25
VV	5	4	5	4	23	1	23	115
VV	5	4	7	4	44,25	1	44,25	309,75
VV	5	4	9	4	28	1	28	252
VV	5	4	11	4	27	1	27	297
VV	5	4	13	4	16,5	1	16,5	214,5
VV	5	4	15	3	55,66666667	0,75	41,75	626,25
VV	5	4	17	3	31,33333333	0,75	23,5	399,5
VV	5	4	19	3	49,66666667	0,75	37,25	707,75
VV	5	4	21	3	42,66666667	0,75	32	672
VV	5	4	23	3	51,66666667	0,75	38,75	891,25
VV	5	4	25	2	40	0,5	20	500
VV	5	4	27	1	46	0,25	11,5	310,5
VV	5	4	29	1	35	0,25	8,75	253,75
VV	5	4	31	1	21	0,25	5,25	162,75
VV	5	4	33	1	16	0,25	4	132
VV	5	4	35	0	0	0	0	0
VV	6	4	3	4	3,75	1	3,75	11,25
VV	6	4	5	4	28,5	1	28,5	142,5
VV	6	4	7	4	39,75	1	39,75	278,25
VV	6	4	9	4	31	1	31	279
VV	6	4	11	4	28,25	1	28,25	310,75
VV	6	4	13	4	19,75	1	19,75	256,75
VV	6	4	15	4	26	1	26	390
VV	6	4	17	4	23,5	1	23,5	399,5
VV	6	4	19	4	24,75	1	24,75	470,25
VV	6	4	21	4	32	1	32	672
VV	6	4	23	2	31,5	0,5	15,75	362,25
VV	6	4	25	1	12	0,25	3	75
VV	6	4	27	0	0	0	0	0
VV	7	4	3	4	6,5	1	6,5	19,5
VV	7	4	5	4	35,25	1	35,25	176,25

Suplemmentary material

VV	7	4	7	4	44,75	1	44,75	313,25
VV	7	4	9	4	38,25	1	38,25	344,25
VV	7	4	11	4	24,75	1	24,75	272,25
VV	7	4	13	4	32,5	1	32,5	422,5
VV	7	4	15	4	12	1	12	180
VV	7	4	17	4	37	1	37	629
VV	7	4	19	4	3,75	1	3,75	71,25
VV	7	4	21	3	61,66666667	0,75	46,25	971,25
VV	7	4	23	3	4	0,75	3	69
VV	7	4	25	3	2,666666667	0,75	2	50
VV	7	4	27	2	0,5	0,5	0,25	6,75
VV	7	4	29	2	0,5	0,5	0,25	7,25
VV	7	4	31	2	2,5	0,5	1,25	38,75
VV	7	4	33	1	1	0,25	0,25	8,25
VV	7	4	35	1	14	0,25	3,5	122,5
VV	7	4	37	0	0	0	0	0
VV	8	4	3	4	8,75	1	8,75	26,25
VV	8	4	5	4	25	1	25	125
VV	8	4	7	4	36,25	1	36,25	253,75
VV	8	4	9	4	39,25	1	39,25	353,25
VV	8	4	11	4	40,25	1	40,25	442,75
VV	8	4	13	4	33,5	1	33,5	435,5
VV	8	4	15	4	31,5	1	31,5	472,5
VV	8	4	17	4	27,5	1	27,5	467,5
VV	8	4	19	4	24,75	1	24,75	470,25
VV	8	4	21	4	23,25	1	23,25	488,25
VV	8	4	23	4	8,75	1	8,75	201,25
VV	8	4	25	4	3,75	1	3,75	93,75
VV	8	4	27	3	0,333333333	0,75	0,25	6,75
VV	8	4	29	3	0	0,75	0	0
VV	8	4	31	3	0	0,75	0	0
VV	8	4	33	3	0	0,75	0	0
VV	8	4	35	3	0,333333333	0,75	0,25	8,75
VV	8	4	37	3	0	0,75	0	0
VV	8	4	39	3	0	0,75	0	0
VV	8	4	41	3	0	0,75	0	0
VV	8	4	43	3	0	0,75	0	0
VV	8	4	45	3	0	0,75	0	0
VV	8	4	47	2	0	0,5	0	0
VV	8	4	49	1	0	0,25	0	0
VV	8	4	51	0	0	0	0	0
VV	1	8	3	8	6,75	1	6,75	20,25
VV	1	8	5	8	30,75	1	30,75	153,75
VV	1	8	7	8	20,375	1	20,375	142,625
VV	1	8	9	8	34,5	1	34,5	310,5

Suplemmentary material

VV	1	8	11	8	24,875	1	24,875	273,625
VV	1	8	13	8	14,375	1	14,375	186,875
VV	1	8	15	8	25,125	1	25,125	376,875
VV	1	8	17	8	12,375	1	12,375	210,375
VV	1	8	19	8	17,625	1	17,625	334,875
VV	1	8	21	7	8	0,875	7	147
VV	1	8	23	7	9,571428571	0,875	8,375	192,625
VV	1	8	25	7	5,428571429	0,875	4,75	118,75
VV	1	8	27	6	4,833333333	0,75	3,625	97,875
VV	1	8	29	6	5,5	0,75	4,125	119,625
VV	1	8	31	6	1,666666667	0,75	1,25	38,75
VV	1	8	33	6	1,833333333	0,75	1,375	45,375
VV	1	8	35	5	1,6	0,625	1	35
VV	1	8	37	3	0,333333333	0,375	0,125	4,625
VV	1	8	39	3	0,666666667	0,375	0,25	9,75
VV	1	8	41	1	0	0,125	0	0
VV	1	8	43	0	0	0	0	0
VV	2	8	3	8	2,25	1	2,25	6,75
VV	2	8	5	8	24,875	1	24,875	124,375
VV	2	8	7	7	29,42857143	0,875	25,75	180,25
VV	2	8	9	7	37,85714286	0,875	33,125	298,125
VV	2	8	11	7	23,85714286	0,875	20,875	229,625
VV	2	8	13	6	39,83333333	0,75	29,875	388,375
VV	2	8	15	6	29,83333333	0,75	22,375	335,625
VV	2	8	17	6	22,33333333	0,75	16,75	284,75
VV	2	8	19	5	32,2	0,625	20,125	382,375
VV	2	8	21	4	25	0,5	12,5	262,5
VV	2	8	23	4	39	0,5	19,5	448,5
VV	2	8	25	4	18	0,5	9	225
VV	2	8	27	4	21	0,5	10,5	283,5
VV	2	8	29	4	24,25	0,5	12,125	351,625
VV	2	8	31	3	7,333333333	0,375	2,75	85,25
VV	2	8	33	3	9,666666667	0,375	3,625	119,625
VV	2	8	35	3	2,666666667	0,375	1	35
VV	2	8	37	3	5	0,375	1,875	69,375
VV	2	8	39	3	1	0,375	0,375	14,625
VV	2	8	41	0	0	0	0	0
VV	3	8	3	8	16	1	16	48
VV	3	8	5	8	33	1	33	165
VV	3	8	7	8	17,125	1	17,125	119,875
VV	3	8	9	8	29,25	1	29,25	263,25
VV	3	8	11	8	30,875	1	30,875	339,625
VV	3	8	13	8	21,5	1	21,5	279,5
VV	3	8	15	8	17,375	1	17,375	260,625
VV	3	8	17	8	17,125	1	17,125	291,125

Suplemmentary material

VV	3	8	19	7	20,28571429	0,875	17,75	337,25
VV	3	8	21	7	18	0,875	15,75	330,75
VV	3	8	23	7	21,28571429	0,875	18,625	428,375
VV	3	8	25	7	8,285714286	0,875	7,25	181,25
VV	3	8	27	7	14,71428571	0,875	12,875	347,625
VV	3	8	29	7	9,428571429	0,875	8,25	239,25
VV	3	8	31	7	12,57142857	0,875	11	341
VV	3	8	33	6	9	0,75	6,75	222,75
VV	3	8	35	6	3,833333333	0,75	2,875	100,625
VV	3	8	37	6	2,5	0,75	1,875	69,375
VV	3	8	39	5	2,6	0,625	1,625	63,375
VV	3	8	41	4	0	0,5	0	0
VV	3	8	43	4	0,75	0,5	0,375	16,125
VV	3	8	45	2	1	0,25	0,25	11,25
VV	3	8	47	0	0	0	0	0
VV	4	8	3	8	6,625	1	6,625	19,875
VV	4	8	5	8	23,25	1	23,25	116,25
VV	4	8	7	8	14,25	1	14,25	99,75
VV	4	8	9	7	23,14285714	0,875	20,25	182,25
VV	4	8	11	7	13,71428571	0,875	12	132
VV	4	8	13	7	11,28571429	0,875	9,875	128,375
VV	4	8	15	6	16,16666667	0,75	12,125	181,875
VV	4	8	17	6	15,66666667	0,75	11,75	199,75
VV	4	8	19	6	17,5	0,75	13,125	249,375
VV	4	8	21	5	15,4	0,625	9,625	202,125
VV	4	8	23	5	22,8	0,625	14,25	327,75
VV	4	8	25	5	17,8	0,625	11,125	278,125
VV	4	8	27	4	24	0,5	12	324
VV	4	8	29	4	26,25	0,5	13,125	380,625
VV	4	8	31	4	18,5	0,5	9,25	286,75
VV	4	8	33	4	12,25	0,5	6,125	202,125
VV	4	8	35	4	14	0,5	7	245
VV	4	8	37	4	9,5	0,5	4,75	175,75
VV	4	8	39	4	3,5	0,5	1,75	68,25
VV	4	8	41	2	3	0,25	0,75	30,75
VV	4	8	43	2	1	0,25	0,25	10,75
VV	4	8	45	2	0,5	0,25	0,125	5,625
VV	4	8	47	2	2	0,25	0,5	23,5
VV	4	8	49	1	1	0,125	0,125	6,125
VV	4	8	51	1	0	0,125	0	0
VV	4	8	53	0	0	0	0	0
VV	5	8	3	8	7,375	1	7,375	22,125
VV	5	8	5	8	17,25	1	17,25	86,25
VV	5	8	7	7	24,28571429	0,875	21,25	148,75
VV	5	8	9	7	29,14285714	0,875	25,5	229,5

Suplemmentary material

VV	5	8	11	7	26,42857143	0,875	23,125	254,375
VV	5	8	13	7	29,85714286	0,875	26,125	339,625
VV	5	8	15	7	23,42857143	0,875	20,5	307,5
VV	5	8	17	7	15,85714286	0,875	13,875	235,875
VV	5	8	19	7	19,57142857	0,875	17,125	325,375
VV	5	8	21	7	14	0,875	12,25	257,25
VV	5	8	23	7	15,71428571	0,875	13,75	316,25
VV	5	8	25	7	14,57142857	0,875	12,75	318,75
VV	5	8	27	7	19,28571429	0,875	16,875	455,625
VV	5	8	29	7	13,85714286	0,875	12,125	351,625
VV	5	8	31	6	16,16666667	0,75	12,125	375,875
VV	5	8	33	6	14,16666667	0,75	10,625	350,625
VV	5	8	35	6	11,83333333	0,75	8,875	310,625
VV	5	8	37	5	9,4	0,625	5,875	217,375
VV	5	8	39	2	9,5	0,25	2,375	92,625
VV	5	8	41	1	8	0,125	1	41
VV	5	8	43	1	7	0,125	0,875	37,625
VV	5	8	45	1	0	0,125	0	0
VV	5	8	47	1	0	0,125	0	0
VV	5	8	49	1	0	0,125	0	0
VV	5	8	51	0	0	0	0	0
VV	6	8	3	8	3	1	3	9
VV	6	8	5	8	15,5	1	15,5	77,5
VV	6	8	7	8	12	1	12	84
VV	6	8	9	8	12,125	1	12,125	109,125
VV	6	8	11	8	9,75	1	9,75	107,25
VV	6	8	13	8	8,375	1	8,375	108,875
VV	6	8	15	8	11,375	1	11,375	170,625
VV	6	8	17	8	10,375	1	10,375	176,375
VV	6	8	19	6	20,83333333	0,75	15,625	296,875
VV	6	8	21	6	14	0,75	10,5	220,5
VV	6	8	23	6	9,333333333	0,75	7	161
VV	6	8	25	6	9,5	0,75	7,125	178,125
VV	6	8	27	4	12,5	0,5	6,25	168,75
VV	6	8	29	4	14	0,5	7	203
VV	6	8	31	4	11,5	0,5	5,75	178,25
VV	6	8	33	4	13,5	0,5	6,75	222,75
VV	6	8	35	4	19	0,5	9,5	332,5
VV	6	8	37	2	11,5	0,25	2,875	106,375
VV	6	8	39	2	6	0,25	1,5	58,5
VV	6	8	41	2	4	0,25	1	41
VV	6	8	43	2	2,5	0,25	0,625	26,875
VV	6	8	45	2	2	0,25	0,5	22,5
VV	6	8	47	1	4	0,125	0,5	23,5
VV	6	8	49	1	0	0,125	0	0

Suplemmentary material

VV	6	8	51	0	0	0	0	0
VV	7	8	3	8	8,875	1	8,875	26,625
VV	7	8	5	8	34	1	34	170
VV	7	8	7	8	23,25	1	23,25	162,75
VV	7	8	9	8	34,875	1	34,875	313,875
VV	7	8	11	8	25,625	1	25,625	281,875
VV	7	8	13	7	31,85714286	0,875	27,875	362,375
VV	7	8	15	7	21	0,875	18,375	275,625
VV	7	8	17	7	16,28571429	0,875	14,25	242,25
VV	7	8	19	7	14,57142857	0,875	12,75	242,25
VV	7	8	21	7	7,857142857	0,875	6,875	144,375
VV	7	8	23	7	8,714285714	0,875	7,625	175,375
VV	7	8	25	6	2,5	0,75	1,875	46,875
VV	7	8	27	5	1,2	0,625	0,75	20,25
VV	7	8	29	5	0,2	0,625	0,125	3,625
VV	7	8	31	5	0	0,625	0	0
VV	7	8	33	5	0	0,625	0	0
VV	7	8	35	5	0	0,625	0	0
VV	7	8	37	4	0	0,5	0	0
VV	7	8	39	3	0,666666667	0,375	0,25	9,75
VV	7	8	41	0	0	0	0	0
VV	8	8	3	8	14,5	1	14,5	43,5
VV	8	8	5	7	43,28571429	0,875	37,875	189,375
VV	8	8	7	7	38,57142857	0,875	33,75	236,25
VV	8	8	9	7	35,85714286	0,875	31,375	282,375
VV	8	8	11	7	34,71428571	0,875	30,375	334,125
VV	8	8	13	7	46	0,875	40,25	523,25
VV	8	8	15	7	38,57142857	0,875	33,75	506,25
VV	8	8	17	7	28,57142857	0,875	25	425
VV	8	8	19	6	34,5	0,75	25,875	491,625
VV	8	8	21	5	29,6	0,625	18,5	388,5
VV	8	8	23	4	36,5	0,5	18,25	419,75
VV	8	8	25	4	34,25	0,5	17,125	428,125
VV	8	8	27	4	34	0,5	17	459
VV	8	8	29	4	18,75	0,5	9,375	271,875
VV	8	8	31	2	21	0,25	5,25	162,75
VV	8	8	33	1	59	0,125	7,375	243,375
VV	8	8	35	1	27	0,125	3,375	118,125
VV	8	8	37	1	31	0,125	3,875	143,375
VV	8	8	39	1	83	0,125	10,375	404,625
VV	8	8	41	1	7	0,125	0,875	35,875
VV	8	8	43	1	1	0,125	0,125	5,375
VV	8	8	45	0	0	0	0	0
VV	1	16	3	16	7,25	1	7,25	21,75
VV	1	16	5	12	21,16666667	0,75	15,875	79,375

Suplemmentary material

VV	1	16	7	12	29	0,75	21,75	152,25
VV	1	16	9	12	21,16666667	0,75	15,875	142,875
VV	1	16	11	12	26,08333333	0,75	19,5625	215,1875
VV	1	16	13	12	21,66666667	0,75	16,25	211,25
VV	1	16	15	12	24,08333333	0,75	18,0625	270,9375
VV	1	16	17	12	21,08333333	0,75	15,8125	268,8125
VV	1	16	19	11	8,545454545	0,6875	5,875	111,625
VV	1	16	21	4	33,5	0,25	8,375	175,875
VV	1	16	23	4	11,25	0,25	2,8125	64,6875
VV	1	16	25	4	7	0,25	1,75	43,75
VV	1	16	27	4	3,5	0,25	0,875	23,625
VV	1	16	29	4	4,5	0,25	1,125	32,625
VV	1	16	31	4	8,75	0,25	2,1875	67,8125
VV	1	16	33	4	3,5	0,25	0,875	28,875
VV	1	16	35	4	0,5	0,25	0,125	4,375
VV	1	16	37	4	0,75	0,25	0,1875	6,9375
VV	1	16	39	2	0	0,125	0	0
VV	1	16	41	0	0	0	0	0
VV	2	16	3	16	1,0625	1	1,0625	3,1875
VV	2	16	5	13	12,84615385	0,8125	10,4375	52,1875
VV	2	16	7	13	31,76923077	0,8125	25,8125	180,6875
VV	2	16	9	13	14,69230769	0,8125	11,9375	107,4375
VV	2	16	11	13	20,84615385	0,8125	16,9375	186,3125
VV	2	16	13	13	26,07692308	0,8125	21,1875	275,4375
VV	2	16	15	13	20,69230769	0,8125	16,8125	252,1875
VV	2	16	17	13	17,92307692	0,8125	14,5625	247,5625
VV	2	16	19	12	16,83333333	0,75	12,625	239,875
VV	2	16	21	12	6,833333333	0,75	5,125	107,625
VV	2	16	23	12	10,33333333	0,75	7,75	178,25
VV	2	16	25	12	10,16666667	0,75	7,625	190,625
VV	2	16	27	10	9,8	0,625	6,125	165,375
VV	2	16	29	8	18,75	0,5	9,375	271,875
VV	2	16	31	8	13,5	0,5	6,75	209,25
VV	2	16	33	6	12,66666667	0,375	4,75	156,75
VV	2	16	35	5	7,6	0,3125	2,375	83,125
VV	2	16	37	5	0,4	0,3125	0,125	4,625
VV	2	16	39	2	2	0,125	0,25	9,75
VV	2	16	41	2	0	0,125	0	0
VV	2	16	43	2	0	0,125	0	0
VV	2	16	45	2	0	0,125	0	0
VV	2	16	47	1	0	0,0625	0	0
VV	2	16	49	0	0	0	0	0
VV	3	16	3	16	1	1	1	3
VV	3	16	5	16	2,8125	1	2,8125	14,0625
VV	3	16	7	16	14,75	1	14,75	103,25

Suplemmentary material

VV	3	16	9	16	14,25	1	14,25	128,25
VV	3	16	11	14	24,64285714	0,875	21,5625	237,1875
VV	3	16	13	14	24,14285714	0,875	21,125	274,625
VV	3	16	15	14	21,78571429	0,875	19,0625	285,9375
VV	3	16	17	12	19,58333333	0,75	14,6875	249,6875
VV	3	16	19	12	14,33333333	0,75	10,75	204,25
VV	3	16	21	12	17,91666667	0,75	13,4375	282,1875
VV	3	16	23	12	11,75	0,75	8,8125	202,6875
VV	3	16	25	12	16,66666667	0,75	12,5	312,5
VV	3	16	27	12	10,08333333	0,75	7,5625	204,1875
VV	3	16	29	12	7,25	0,75	5,4375	157,6875
VV	3	16	31	12	8,333333333	0,75	6,25	193,75
VV	3	16	33	12	5,583333333	0,75	4,1875	138,1875
VV	3	16	35	11	3,909090909	0,6875	2,6875	94,0625
VV	3	16	37	8	1,25	0,5	0,625	23,125
VV	3	16	39	6	0,333333333	0,375	0,125	4,875
VV	3	16	41	4	0,25	0,25	0,0625	2,5625
VV	3	16	43	2	1,5	0,125	0,1875	8,0625
VV	3	16	45	0	0	0	0	0
VV	4	16	3	16	5,3125	1	5,3125	15,9375
VV	4	16	5	16	5,9375	1	5,9375	29,6875
VV	4	16	7	16	4,625	1	4,625	32,375
VV	4	16	9	16	4,875	1	4,875	43,875
VV	4	16	11	15	7,4	0,9375	6,9375	76,3125
VV	4	16	13	15	5,6	0,9375	5,25	68,25
VV	4	16	15	15	3,866666667	0,9375	3,625	54,375
VV	4	16	17	15	4,4	0,9375	4,125	70,125
VV	4	16	19	15	2,4	0,9375	2,25	42,75
VV	4	16	21	15	0,933333333	0,9375	0,875	18,375
VV	4	16	23	12	1	0,75	0,75	17,25
VV	4	16	25	10	0,9	0,625	0,5625	14,0625
VV	4	16	27	9	0,333333333	0,5625	0,1875	5,0625
VV	4	16	29	7	0	0,4375	0	0
VV	4	16	31	6	0	0,375	0	0
VV	4	16	33	5	0	0,3125	0	0
VV	4	16	35	4	0	0,25	0	0
VV	4	16	37	4	0	0,25	0	0
VV	4	16	39	3	0	0,1875	0	0
VV	4	16	41	0	0	0	0	0
VV	5	16	3	16	7,625	1	7,625	22,875
VV	5	16	5	15	21,66666667	0,9375	20,3125	101,5625
VV	5	16	7	14	37,28571429	0,875	32,625	228,375
VV	5	16	9	14	26	0,875	22,75	204,75
VV	5	16	11	14	28,42857143	0,875	24,875	273,625
VV	5	16	13	14	27,57142857	0,875	24,125	313,625

Suplemmentary material

VV	5	16	15	14	26,64285714	0,875	23,3125	349,6875
VV	5	16	17	14	10,92857143	0,875	9,5625	162,5625
VV	5	16	19	14	20,35714286	0,875	17,8125	338,4375
VV	5	16	21	13	20,46153846	0,8125	16,625	349,125
VV	5	16	23	13	15,30769231	0,8125	12,4375	286,0625
VV	5	16	25	13	18,84615385	0,8125	15,3125	382,8125
VV	5	16	27	13	16,53846154	0,8125	13,4375	362,8125
VV	5	16	29	10	12,9	0,625	8,0625	233,8125
VV	5	16	31	10	15,4	0,625	9,625	298,375
VV	5	16	33	9	8	0,5625	4,5	148,5
VV	5	16	35	6	4	0,375	1,5	52,5
VV	5	16	37	0	0	0	0	0
VV	6	16	3	16	3,5625	1	3,5625	10,6875
VV	6	16	5	15	20,73333333	0,9375	19,4375	97,1875
VV	6	16	7	15	28,93333333	0,9375	27,125	189,875
VV	6	16	9	15	23,53333333	0,9375	22,0625	198,5625
VV	6	16	11	15	26,06666667	0,9375	24,4375	268,8125
VV	6	16	13	14	26,35714286	0,875	23,0625	299,8125
VV	6	16	15	14	29,78571429	0,875	26,0625	390,9375
VV	6	16	17	14	23,78571429	0,875	20,8125	353,8125
VV	6	16	19	14	30,07142857	0,875	26,3125	499,9375
VV	6	16	21	13	10,38461538	0,8125	8,4375	177,1875
VV	6	16	23	13	18,76923077	0,8125	15,25	350,75
VV	6	16	25	13	20,92307692	0,8125	17	425
VV	6	16	27	13	19,23076923	0,8125	15,625	421,875
VV	6	16	29	12	14,75	0,75	11,0625	320,8125
VV	6	16	31	12	23,66666667	0,75	17,75	550,25
VV	6	16	33	10	13	0,625	8,125	268,125
VV	6	16	35	9	10,44444444	0,5625	5,875	205,625
VV	6	16	37	3	0,666666667	0,1875	0,125	4,625
VV	6	16	39	3	0	0,1875	0	0
VV	6	16	41	2	0,5	0,125	0,0625	2,5625
VV	6	16	43	1	0	0,0625	0	0
VV	6	16	45	0	0	0	0	0
VV	7	16	3	16	5,875	1	5,875	17,625
VV	7	16	5	16	27,1875	1	27,1875	135,9375
VV	7	16	7	15	31,4	0,9375	29,4375	206,0625
VV	7	16	9	15	20,66666667	0,9375	19,375	174,375
VV	7	16	11	15	30,73333333	0,9375	28,8125	316,9375
VV	7	16	13	14	36,21428571	0,875	31,6875	411,9375
VV	7	16	15	14	24	0,875	21	315
VV	7	16	17	14	27	0,875	23,625	401,625
VV	7	16	19	13	22,46153846	0,8125	18,25	346,75
VV	7	16	21	13	10,92307692	0,8125	8,875	186,375
VV	7	16	23	13	23,23076923	0,8125	18,875	434,125

Suplemmentary material

VV	7	16	25	12	19,83333333	0,75	14,875	371,875
VV	7	16	27	11	15,45454545	0,6875	10,625	286,875
VV	7	16	29	11	12,90909091	0,6875	8,875	257,375
VV	7	16	31	9	6,888888889	0,5625	3,875	120,125
VV	7	16	33	8	6	0,5	3	99
VV	7	16	35	6	2	0,375	0,75	26,25
VV	7	16	37	4	0,25	0,25	0,0625	2,3125
VV	7	16	39	4	0,5	0,25	0,125	4,875
VV	7	16	41	4	0	0,25	0	0
VV	7	16	43	2	1,5	0,125	0,1875	8,0625
VV	7	16	45	0	0	0	0	0
VV	8	16	3	16	21,75	1	21,75	65,25
VV	8	16	5	16	24,6875	1	24,6875	123,4375
VV	8	16	7	16	24,625	1	24,625	172,375
VV	8	16	9	16	31,1875	1	31,1875	280,6875
VV	8	16	11	16	17,8125	1	17,8125	195,9375
VV	8	16	13	12	20,16666667	0,75	15,125	196,625
VV	8	16	15	12	19,91666667	0,75	14,9375	224,0625
VV	8	16	17	12	23,66666667	0,75	17,75	301,75
VV	8	16	19	12	22,41666667	0,75	16,8125	319,4375
VV	8	16	21	12	26,66666667	0,75	20	420
VV	8	16	23	12	30	0,75	22,5	517,5
VV	8	16	25	12	12,16666667	0,75	9,125	228,125
VV	8	16	27	12	16,16666667	0,75	12,125	327,375
VV	8	16	29	11	14,09090909	0,6875	9,6875	280,9375
VV	8	16	31	9	6,666666667	0,5625	3,75	116,25
VV	8	16	33	5	10,4	0,3125	3,25	107,25
VV	8	16	35	3	4,333333333	0,1875	0,8125	28,4375
VV	8	16	37	2	13	0,125	1,625	60,125
VV	8	16	39	2	8	0,125	1	39
VV	8	16	41	1	1	0,0625	0,0625	2,5625
VV	8	16	43	1	0	0,0625	0	0
VV	8	16	45	0	0	0	0	0
VC	1	2	3	2	13,5	1	13,5	40,5
VC	1	2	5	2	33	1	33	165
VC	1	2	7	2	28	1	28	196
VC	1	2	9	2	34	1	34	306
VC	1	2	11	2	28,5	1	28,5	313,5
VC	1	2	13	2	42,5	1	42,5	552,5
VC	1	2	15	2	24	1	24	360
VC	1	2	17	2	38,5	1	38,5	654,5
VC	1	2	19	1	33	0,5	16,5	313,5
VC	1	2	21	1	45	0,5	22,5	472,5
VC	1	2	23	1	36	0,5	18	414
VC	1	2	25	1	24	0,5	12	300

Suplemmentary material

VC	1	2	27	1	14	0,5	7	189
VC	1	2	29	1	3	0,5	1,5	43,5
VC	1	2	31	0	0	0	0	0
VC	2	2	3	2	15	1	15	45
VC	2	2	5	2	23	1	23	115
VC	2	2	7	2	37	1	37	259
VC	2	2	9	2	36	1	36	324
VC	2	2	11	2	41,5	1	41,5	456,5
VC	2	2	13	2	43,5	1	43,5	565,5
VC	2	2	15	2	19	1	19	285
VC	2	2	17	2	22,5	1	22,5	382,5
VC	2	2	19	2	12	1	12	228
VC	2	2	21	2	7	1	7	147
VC	2	2	23	2	9,5	1	9,5	218,5
VC	2	2	25	2	24,5	1	24,5	612,5
VC	2	2	27	2	0	1	0	0
VC	2	2	29	1	0	0,5	0	0
VC	2	2	31	0	0	0	0	0
VC	3	2	3	2	20	1	20	60
VC	3	2	5	2	59	1	59	295
VC	3	2	7	2	41	1	41	287
VC	3	2	9	2	38	1	38	342
VC	3	2	11	2	46	1	46	506
VC	3	2	13	2	40	1	40	520
VC	3	2	15	2	37	1	37	555
VC	3	2	17	2	58,5	1	58,5	994,5
VC	3	2	19	2	75,5	1	75,5	1434,5
VC	3	2	21	2	28,5	1	28,5	598,5
VC	3	2	23	2	41	1	41	943
VC	3	2	25	2	31	1	31	775
VC	3	2	27	2	26,5	1	26,5	715,5
VC	3	2	29	2	34,5	1	34,5	1000,5
VC	3	2	31	1	33	0,5	16,5	511,5
VC	3	2	33	1	25	0,5	12,5	412,5
VC	3	2	35	1	9	0,5	4,5	157,5
VC	3	2	37	1	9	0,5	4,5	166,5
VC	3	2	39	0	0	0	0	0
VC	4	2	3	2	15,5	1	15,5	46,5
VC	4	2	5	2	47,5	1	47,5	237,5
VC	4	2	7	2	40,5	1	40,5	283,5
VC	4	2	9	2	48,5	1	48,5	436,5
VC	4	2	11	2	34,5	1	34,5	379,5
VC	4	2	13	2	35,5	1	35,5	461,5
VC	4	2	15	2	31,5	1	31,5	472,5
VC	4	2	17	2	22	1	22	374

Suplemmentary material

VC	4	2	19	2	1,5	1	1,5	28,5
VC	4	2	21	1	4	0,5	2	42
VC	4	2	23	1	0	0,5	0	0
VC	4	2	25	1	2	0,5	1	25
VC	4	2	27	1	0	0,5	0	0
VC	4	2	29	0	0	0	0	0
VC	5	2	3	2	13,5	1	13,5	40,5
VC	5	2	5	2	52,5	1	52,5	262,5
VC	5	2	7	2	38	1	38	266
VC	5	2	9	2	0	1	0	0
VC	5	2	11	2	0	1	0	0
VC	5	2	13	1	0	0,5	0	0
VC	5	2	15	1	0	0,5	0	0
VC	5	2	17	1	0	0,5	0	0
VC	5	2	19	1	0	0,5	0	0
VC	5	2	21	0	0	0	0	0
VC	6	2	3	2	1	1	1	3
VC	6	2	5	2	44,5	1	44,5	222,5
VC	6	2	7	2	26	1	26	182
VC	6	2	9	2	29	1	29	261
VC	6	2	11	2	31,5	1	31,5	346,5
VC	6	2	13	2	29,5	1	29,5	383,5
VC	6	2	15	2	41,5	1	41,5	622,5
VC	6	2	17	2	28	1	28	476
VC	6	2	19	2	14,5	1	14,5	275,5
VC	6	2	21	2	14	1	14	294
VC	6	2	23	2	11	1	11	253
VC	6	2	25	2	5	1	5	125
VC	6	2	27	1	0	0,5	0	0
VC	6	2	29	1	0	0,5	0	0
VC	6	2	31	1	0	0,5	0	0
VC	6	2	33	1	0	0,5	0	0
VC	6	2	35	1	0	0,5	0	0
VC	6	2	37	0	0	0	0	0
VC	7	2	3	2	17	1	17	51
VC	7	2	5	2	54	1	54	270
VC	7	2	7	2	41	1	41	287
VC	7	2	9	2	72	1	72	648
VC	7	2	11	2	38,5	1	38,5	423,5
VC	7	2	13	2	41,5	1	41,5	539,5
VC	7	2	15	2	54	1	54	810
VC	7	2	17	2	54,5	1	54,5	926,5
VC	7	2	19	2	39	1	39	741
VC	7	2	21	2	44,5	1	44,5	934,5
VC	7	2	23	2	42	1	42	966

Suplemmentary material

VC	7	2	25	2	36	1	36	900
VC	7	2	27	2	54,5	1	54,5	1471,5
VC	7	2	29	1	24	0,5	12	348
VC	7	2	31	1	17	0,5	8,5	263,5
VC	7	2	33	0	0	0	0	0
VC	8	2	3	2	11,5	1	11,5	34,5
VC	8	2	5	2	45,5	1	45,5	227,5
VC	8	2	7	2	38,5	1	38,5	269,5
VC	8	2	9	2	42,5	1	42,5	382,5
VC	8	2	11	2	40	1	40	440
VC	8	2	13	2	28,5	1	28,5	370,5
VC	8	2	15	2	30,5	1	30,5	457,5
VC	8	2	17	2	36,5	1	36,5	620,5
VC	8	2	19	2	32,5	1	32,5	617,5
VC	8	2	21	2	31	1	31	651
VC	8	2	23	2	17	1	17	391
VC	8	2	25	2	30	1	30	750
VC	8	2	27	2	25,5	1	25,5	688,5
VC	8	2	29	2	5,5	1	5,5	159,5
VC	8	2	31	2	17,5	1	17,5	542,5
VC	8	2	33	1	17	0,5	8,5	280,5
VC	8	2	35	0	0	0	0	0
VC	1	4	3	4	34,25	1	34,25	102,75
VC	1	4	5	4	35,5	1	35,5	177,5
VC	1	4	7	4	29,25	1	29,25	204,75
VC	1	4	9	4	41,25	1	41,25	371,25
VC	1	4	11	4	48,75	1	48,75	536,25
VC	1	4	13	4	49	1	49	637
VC	1	4	15	4	35	1	35	525
VC	1	4	17	4	29	1	29	493
VC	1	4	19	4	33	1	33	627
VC	1	4	21	4	34	1	34	714
VC	1	4	23	4	17,75	1	17,75	408,25
VC	1	4	25	4	2,75	1	2,75	68,75
VC	1	4	27	4	1,25	1	1,25	33,75
VC	1	4	29	4	4,25	1	4,25	123,25
VC	1	4	31	4	8,5	1	8,5	263,5
VC	1	4	33	4	4,25	1	4,25	140,25
VC	1	4	35	4	7,5	1	7,5	262,5
VC	1	4	37	4	2,25	1	2,25	83,25
VC	1	4	39	4	5,5	1	5,5	214,5
VC	1	4	41	4	0	1	0	0
VC	1	4	43	1	0	0,25	0	0
VC	1	4	45	0	0	0	0	0
VC	2	4	3	4	26	1	26	78

Suplemmentary material

VC	2	4	5	4	46,5	1	46,5	232,5
VC	2	4	7	4	32	1	32	224
VC	2	4	9	4	51	1	51	459
VC	2	4	11	4	31	1	31	341
VC	2	4	13	4	19,25	1	19,25	250,25
VC	2	4	15	4	21,25	1	21,25	318,75
VC	2	4	17	4	17,25	1	17,25	293,25
VC	2	4	19	4	28	1	28	532
VC	2	4	21	4	25	1	25	525
VC	2	4	23	4	14,75	1	14,75	339,25
VC	2	4	25	4	9	1	9	225
VC	2	4	27	4	9,5	1	9,5	256,5
VC	2	4	29	4	8,5	1	8,5	246,5
VC	2	4	31	4	2,75	1	2,75	85,25
VC	2	4	33	4	5	1	5	165
VC	2	4	35	4	2	1	2	70
VC	2	4	37	4	0,5	1	0,5	18,5
VC	2	4	39	4	0	1	0	0
VC	2	4	41	2	0	0,5	0	0
VC	2	4	43	2	0	0,5	0	0
VC	2	4	45	1	0	0,25	0	0
VC	2	4	47	1	0	0,25	0	0
VC	2	4	49	0	0	0	0	0
VC	3	4	3	4	52	1	52	156
VC	3	4	5	4	59,25	1	59,25	296,25
VC	3	4	7	4	66,75	1	66,75	467,25
VC	3	4	9	4	72,25	1	72,25	650,25
VC	3	4	11	4	78,5	1	78,5	863,5
VC	3	4	13	4	68,5	1	68,5	890,5
VC	3	4	15	4	46,25	1	46,25	693,75
VC	3	4	17	4	45,5	1	45,5	773,5
VC	3	4	19	4	63,5	1	63,5	1206,5
VC	3	4	21	4	32	1	32	672
VC	3	4	23	3	35,66666667	0,75	26,75	615,25
VC	3	4	25	3	29	0,75	21,75	543,75
VC	3	4	27	3	16,33333333	0,75	12,25	330,75
VC	3	4	29	3	8,666666667	0,75	6,5	188,5
VC	3	4	31	3	4,333333333	0,75	3,25	100,75
VC	3	4	33	2	8	0,5	4	132
VC	3	4	35	1	0	0,25	0	0
VC	3	4	37	1	0	0,25	0	0
VC	3	4	39	1	0	0,25	0	0
VC	3	4	41	1	0	0,25	0	0
VC	3	4	43	0	0	0	0	0
VC	4	4	3	4	41	1	41	123

Suplemmentary material

VC	4	4	5	4	42,25	1	42,25	211,25
VC	4	4	7	4	51,5	1	51,5	360,5
VC	4	4	9	4	51,75	1	51,75	465,75
VC	4	4	11	4	69	1	69	759
VC	4	4	13	4	30	1	30	390
VC	4	4	15	4	35,75	1	35,75	536,25
VC	4	4	17	4	32,5	1	32,5	552,5
VC	4	4	19	4	34,5	1	34,5	655,5
VC	4	4	21	4	32,25	1	32,25	677,25
VC	4	4	23	4	25,25	1	25,25	580,75
VC	4	4	25	4	26	1	26	650
VC	4	4	27	4	22	1	22	594
VC	4	4	29	3	32	0,75	24	696
VC	4	4	31	3	31	0,75	23,25	720,75
VC	4	4	33	2	26,5	0,5	13,25	437,25
VC	4	4	35	2	43,5	0,5	21,75	761,25
VC	4	4	37	2	49	0,5	24,5	906,5
VC	4	4	39	2	26,5	0,5	13,25	516,75
VC	4	4	41	2	31	0,5	15,5	635,5
VC	4	4	43	2	26	0,5	13	559
VC	4	4	45	2	22	0,5	11	495
VC	4	4	47	2	0	0,5	0	0
VC	4	4	49	0	0	0	0	0
VC	5	4	3	4	39	1	39	117
VC	5	4	5	4	60,5	1	60,5	302,5
VC	5	4	7	4	30,5	1	30,5	213,5
VC	5	4	9	4	24	1	24	216
VC	5	4	11	4	37,5	1	37,5	412,5
VC	5	4	13	4	45,25	1	45,25	588,25
VC	5	4	15	4	22,5	1	22,5	337,5
VC	5	4	17	4	31,25	1	31,25	531,25
VC	5	4	19	4	24	1	24	456
VC	5	4	21	3	20,66666667	0,75	15,5	325,5
VC	5	4	23	3	11	0,75	8,25	189,75
VC	5	4	25	3	4	0,75	3	75
VC	5	4	27	2	0	0,5	0	0
VC	5	4	29	2	0	0,5	0	0
VC	5	4	31	2	2,5	0,5	1,25	38,75
VC	5	4	33	0	0	0	0	0
VC	7	4	3	4	11,5	1	11,5	34,5
VC	7	4	5	3	54	0,75	40,5	202,5
VC	7	4	7	3	50	0,75	37,5	262,5
VC	7	4	9	3	49,33333333	0,75	37	333
VC	7	4	11	3	35,66666667	0,75	26,75	294,25
VC	7	4	13	3	37,33333333	0,75	28	364

Suplemmentary material

VC	7	4	15	3	35,33333333	0,75	26,5	397,5
VC	7	4	17	3	16,66666667	0,75	12,5	212,5
VC	7	4	19	3	3	0,75	2,25	42,75
VC	7	4	21	3	0	0,75	0	0
VC	7	4	23	3	3	0,75	2,25	51,75
VC	7	4	25	2	11,5	0,5	5,75	143,75
VC	7	4	27	2	3	0,5	1,5	40,5
VC	7	4	29	2	13,5	0,5	6,75	195,75
VC	7	4	31	2	0,5	0,5	0,25	7,75
VC	7	4	33	2	1	0,5	0,5	16,5
VC	7	4	35	2	3	0,5	1,5	52,5
VC	7	4	37	2	1	0,5	0,5	18,5
VC	7	4	39	2	0	0,5	0	0
VC	7	4	41	2	1	0,5	0,5	20,5
VC	7	4	43	2	2	0,5	1	43
VC	7	4	45	2	7	0,5	3,5	157,5
VC	7	4	47	2	5	0,5	2,5	117,5
VC	7	4	49	2	0	0,5	0	0
VC	7	4	51	2	0	0,5	0	0
VC	7	4	53	1	0	0,25	0	0
VC	7	4	55	0	0	0	0	0
VC	8	4	3	4	54	1	54	162
VC	8	4	5	4	26	1	26	130
VC	8	4	7	4	48,75	1	48,75	341,25
VC	8	4	9	4	46,75	1	46,75	420,75
VC	8	4	11	4	58	1	58	638
VC	8	4	13	4	64,25	1	64,25	835,25
VC	8	4	15	2	2,5	0,5	1,25	18,75
VC	8	4	17	1	2	0,25	0,5	8,5
VC	8	4	19	0	0	0	0	0
VC	1	8	3	8	20	1	20	60
VC	1	8	5	8	22,75	1	22,75	113,75
VC	1	8	7	7	26,42857143	0,875	23,125	161,875
VC	1	8	9	6	27,33333333	0,75	20,5	184,5
VC	1	8	11	6	29,83333333	0,75	22,375	246,125
VC	1	8	13	6	16,66666667	0,75	12,5	162,5
VC	1	8	15	6	13,66666667	0,75	10,25	153,75
VC	1	8	17	6	33,66666667	0,75	25,25	429,25
VC	1	8	19	6	21,33333333	0,75	16	304
VC	1	8	21	5	20,8	0,625	13	273
VC	1	8	23	5	20,4	0,625	12,75	293,25
VC	1	8	25	5	18,2	0,625	11,375	284,375
VC	1	8	27	5	9,2	0,625	5,75	155,25
VC	1	8	29	5	8,4	0,625	5,25	152,25
VC	1	8	31	5	2	0,625	1,25	38,75

Suplemmentary material

VC	1	8	33	5	0	0,625	0	0
VC	1	8	35	5	0	0,625	0	0
VC	1	8	37	5	0	0,625	0	0
VC	1	8	39	3	1	0,375	0,375	14,625
VC	1	8	41	1	0	0,125	0	0
VC	1	8	43	1	0	0,125	0	0
VC	1	8	45	1	0	0,125	0	0
VC	1	8	47	1	0	0,125	0	0
VC	1	8	49	0	0	0	0	0
VC	2	8	3	8	39,625	1	39,625	118,875
VC	2	8	5	8	32,5	1	32,5	162,5
VC	2	8	7	8	33,625	1	33,625	235,375
VC	2	8	9	8	37,25	1	37,25	335,25
VC	2	8	11	7	41,28571429	0,875	36,125	397,375
VC	2	8	13	7	33	0,875	28,875	375,375
VC	2	8	15	7	33,71428571	0,875	29,5	442,5
VC	2	8	17	7	24,28571429	0,875	21,25	361,25
VC	2	8	19	7	21,57142857	0,875	18,875	358,625
VC	2	8	21	7	20,14285714	0,875	17,625	370,125
VC	2	8	23	7	12,57142857	0,875	11	253
VC	2	8	25	7	13	0,875	11,375	284,375
VC	2	8	27	7	21,57142857	0,875	18,875	509,625
VC	2	8	29	7	1,285714286	0,875	1,125	32,625
VC	2	8	31	6	3	0,75	2,25	69,75
VC	2	8	33	3	0	0,375	0	0
VC	2	8	35	0	0	0	0	0
VC	3	8	3	8	30	1	30	90
VC	3	8	5	8	29,5	1	29,5	147,5
VC	3	8	7	8	27,625	1	27,625	193,375
VC	3	8	9	8	33,25	1	33,25	299,25
VC	3	8	11	8	18,875	1	18,875	207,625
VC	3	8	13	8	30	1	30	390
VC	3	8	15	8	22,125	1	22,125	331,875
VC	3	8	17	7	34,71428571	0,875	30,375	516,375
VC	3	8	19	7	19,71428571	0,875	17,25	327,75
VC	3	8	21	7	20,14285714	0,875	17,625	370,125
VC	3	8	23	7	24,71428571	0,875	21,625	497,375
VC	3	8	25	7	13,42857143	0,875	11,75	293,75
VC	3	8	27	7	19,14285714	0,875	16,75	452,25
VC	3	8	29	7	9,714285714	0,875	8,5	246,5
VC	3	8	31	7	8,142857143	0,875	7,125	220,875
VC	3	8	33	7	10,14285714	0,875	8,875	292,875
VC	3	8	35	7	0	0,875	0	0
VC	3	8	37	7	0	0,875	0	0
VC	3	8	39	6	0	0,75	0	0

Suplemmentary material

VC	3	8	41	2	0	0,25	0	0
VC	3	8	43	2	0	0,25	0	0
VC	3	8	45	2	0	0,25	0	0
VC	3	8	47	0	0	0	0	0
VC	4	8	3	8	38,875	1	38,875	116,625
VC	4	8	5	8	35,125	1	35,125	175,625
VC	4	8	7	8	22,25	1	22,25	155,75
VC	4	8	9	8	31,875	1	31,875	286,875
VC	4	8	11	7	23	0,875	20,125	221,375
VC	4	8	13	7	30,57142857	0,875	26,75	347,75
VC	4	8	15	7	28,71428571	0,875	25,125	376,875
VC	4	8	17	6	32,33333333	0,75	24,25	412,25
VC	4	8	19	6	17,66666667	0,75	13,25	251,75
VC	4	8	21	6	6,333333333	0,75	4,75	99,75
VC	4	8	23	6	10,83333333	0,75	8,125	186,875
VC	4	8	25	6	8,333333333	0,75	6,25	156,25
VC	4	8	27	6	15,83333333	0,75	11,875	320,625
VC	4	8	29	5	9,6	0,625	6	174
VC	4	8	31	5	5,2	0,625	3,25	100,75
VC	4	8	33	5	6,8	0,625	4,25	140,25
VC	4	8	35	4	7	0,5	3,5	122,5
VC	4	8	37	4	7,25	0,5	3,625	134,125
VC	4	8	39	4	4,25	0,5	2,125	82,875
VC	4	8	41	3	9,333333333	0,375	3,5	143,5
VC	4	8	43	3	2	0,375	0,75	32,25
VC	4	8	45	3	0	0,375	0	0
VC	4	8	47	3	0	0,375	0	0
VC	4	8	49	3	0	0,375	0	0
VC	4	8	51	3	0	0,375	0	0
VC	4	8	53	0	0	0	0	0
VC	5	8	3	8	37,375	1	37,375	112,125
VC	5	8	5	8	33,5	1	33,5	167,5
VC	5	8	7	8	23,25	1	23,25	162,75
VC	5	8	9	8	28,125	1	28,125	253,125
VC	5	8	11	8	27,875	1	27,875	306,625
VC	5	8	13	8	30,5	1	30,5	396,5
VC	5	8	15	8	24,25	1	24,25	363,75
VC	5	8	17	8	32	1	32	544
VC	5	8	19	8	31,125	1	31,125	591,375
VC	5	8	21	8	7,125	1	7,125	149,625
VC	5	8	23	8	6,25	1	6,25	143,75
VC	5	8	25	8	12,25	1	12,25	306,25
VC	5	8	27	8	12,125	1	12,125	327,375
VC	5	8	29	8	8,75	1	8,75	253,75
VC	5	8	31	8	2,75	1	2,75	85,25

Suplemmentary material

VC	5	8	33	8	1,875	1	1,875	61,875
VC	5	8	35	8	9,375	1	9,375	328,125
VC	5	8	37	7	5,142857143	0,875	4,5	166,5
VC	5	8	39	7	9,428571429	0,875	8,25	321,75
VC	5	8	41	7	2,571428571	0,875	2,25	92,25
VC	5	8	43	7	0	0,875	0	0
VC	5	8	45	7	0	0,875	0	0
VC	5	8	47	6	0	0,75	0	0
VC	5	8	49	5	0	0,625	0	0
VC	5	8	51	3	0	0,375	0	0
VC	5	8	53	1	0	0,125	0	0
VC	5	8	55	1	0	0,125	0	0
VC	5	8	57	1	0	0,125	0	0
VC	5	8	59	0	0	0	0	0
VC	6	8	3	8	21,25	1	21,25	63,75
VC	6	8	5	8	13	1	13	65
VC	6	8	7	8	16,25	1	16,25	113,75
VC	6	8	9	8	17,625	1	17,625	158,625
VC	6	8	11	7	12	0,875	10,5	115,5
VC	6	8	13	6	12,83333333	0,75	9,625	125,125
VC	6	8	15	6	8,333333333	0,75	6,25	93,75
VC	6	8	17	6	7,833333333	0,75	5,875	99,875
VC	6	8	19	6	11,33333333	0,75	8,5	161,5
VC	6	8	21	6	24,5	0,75	18,375	385,875
VC	6	8	23	5	17,8	0,625	11,125	255,875
VC	6	8	25	5	7	0,625	4,375	109,375
VC	6	8	27	5	4,4	0,625	2,75	74,25
VC	6	8	29	5	8	0,625	5	145
VC	6	8	31	5	7	0,625	4,375	135,625
VC	6	8	33	4	7	0,5	3,5	115,5
VC	6	8	35	4	6,25	0,5	3,125	109,375
VC	6	8	37	4	5	0,5	2,5	92,5
VC	6	8	39	4	2,25	0,5	1,125	43,875
VC	6	8	41	4	1,5	0,5	0,75	30,75
VC	6	8	43	4	0,5	0,5	0,25	10,75
VC	6	8	45	4	0	0,5	0	0
VC	6	8	47	3	0	0,375	0	0
VC	6	8	49	1	0	0,125	0	0
VC	6	8	51	1	0	0,125	0	0
VC	6	8	53	0	0	0	0	0
VC	7	8	3	8	11,25	1	11,25	33,75
VC	7	8	5	8	18,375	1	18,375	91,875
VC	7	8	7	8	13,125	1	13,125	91,875
VC	7	8	9	8	14,5	1	14,5	130,5
VC	7	8	11	6	18	0,75	13,5	148,5

Suplemmentary material

VC	7	8	13	5	24,2	0,625	15,125	196,625
VC	7	8	15	5	24,2	0,625	15,125	226,875
VC	7	8	17	5	59,6	0,625	37,25	633,25
VC	7	8	19	5	41,2	0,625	25,75	489,25
VC	7	8	21	5	17,8	0,625	11,125	233,625
VC	7	8	23	4	0	0,5	0	0
VC	7	8	25	3	8	0,375	3	75
VC	7	8	27	3	2	0,375	0,75	20,25
VC	7	8	29	3	7,666666667	0,375	2,875	83,375
VC	7	8	31	3	1,333333333	0,375	0,5	15,5
VC	7	8	33	3	5,666666667	0,375	2,125	70,125
VC	7	8	35	3	6,333333333	0,375	2,375	83,125
VC	7	8	37	2	3,5	0,25	0,875	32,375
VC	7	8	39	2	0	0,25	0	0
VC	7	8	41	2	0	0,25	0	0
VC	7	8	43	2	0,5	0,25	0,125	5,375
VC	7	8	45	2	1	0,25	0,25	11,25
VC	7	8	47	2	1	0,25	0,25	11,75
VC	7	8	49	2	0,5	0,25	0,125	6,125
VC	7	8	51	2	0	0,25	0	0
VC	7	8	53	1	0	0,125	0	0
VC	7	8	55	1	0	0,125	0	0
VC	7	8	57	1	0	0,125	0	0
VC	7	8	59	0	0	0	0	0
VC	8	8	3	8	16,75	1	16,75	50,25
VC	8	8	5	7	29,71428571	0,875	26	130
VC	8	8	7	6	37,16666667	0,75	27,875	195,125
VC	8	8	9	6	34,16666667	0,75	25,625	230,625
VC	8	8	11	6	35,16666667	0,75	26,375	290,125
VC	8	8	13	6	37,16666667	0,75	27,875	362,375
VC	8	8	15	6	31,66666667	0,75	23,75	356,25
VC	8	8	17	6	26,16666667	0,75	19,625	333,625
VC	8	8	19	6	22,5	0,75	16,875	320,625
VC	8	8	21	5	38,4	0,625	24	504
VC	8	8	23	5	19,2	0,625	12	276
VC	8	8	25	5	8,6	0,625	5,375	134,375
VC	8	8	27	4	5,25	0,5	2,625	70,875
VC	8	8	29	4	7	0,5	3,5	101,5
VC	8	8	31	4	6,25	0,5	3,125	96,875
VC	8	8	33	4	4	0,5	2	66
VC	8	8	35	4	2	0,5	1	35
VC	8	8	37	4	0	0,5	0	0
VC	8	8	39	4	1	0,5	0,5	19,5
VC	8	8	41	4	0,5	0,5	0,25	10,25
VC	8	8	43	4	0,25	0,5	0,125	5,375

Suplemmentary material

VC	8	8	45	1	0	0,125	0	0
VC	8	8	47	1	0	0,125	0	0
VC	8	8	49	1	0	0,125	0	0
VC	8	8	51	0	0	0	0	0
VC	1	16	3	16	23,75	1	23,75	71,25
VC	1	16	5	16	24,4375	1	24,4375	122,1875
VC	1	16	7	16	14,125	1	14,125	98,875
VC	1	16	9	16	16,8125	1	16,8125	151,3125
VC	1	16	11	16	20,6875	1	20,6875	227,5625
VC	1	16	13	16	19,875	1	19,875	258,375
VC	1	16	15	16	16,6875	1	16,6875	250,3125
VC	1	16	17	16	12,125	1	12,125	206,125
VC	1	16	19	16	14,8125	1	14,8125	281,4375
VC	1	16	21	15	9,666666667	0,9375	9,0625	190,3125
VC	1	16	23	15	16,06666667	0,9375	15,0625	346,4375
VC	1	16	25	15	11,46666667	0,9375	10,75	268,75
VC	1	16	27	15	11,86666667	0,9375	11,125	300,375
VC	1	16	29	13	12,76923077	0,8125	10,375	300,875
VC	1	16	31	13	5,538461538	0,8125	4,5	139,5
VC	1	16	33	13	5	0,8125	4,0625	134,0625
VC	1	16	35	12	6,833333333	0,75	5,125	179,375
VC	1	16	37	12	1,25	0,75	0,9375	34,6875
VC	1	16	39	6	0,333333333	0,375	0,125	4,875
VC	1	16	41	2	0,5	0,125	0,0625	2,5625
VC	1	16	43	1	2	0,0625	0,125	5,375
VC	1	16	45	1	3	0,0625	0,1875	8,4375
VC	1	16	47	1	0	0,0625	0	0
VC	1	16	49	0	0	0	0	0
VC	2	16	3	16	9,75	1	9,75	29,25
VC	2	16	5	16	13,8125	1	13,8125	69,0625
VC	2	16	7	16	20,5	1	20,5	143,5
VC	2	16	9	16	16,5	1	16,5	148,5
VC	2	16	11	16	20,875	1	20,875	229,625
VC	2	16	13	16	15,625	1	15,625	203,125
VC	2	16	15	15	22,26666667	0,9375	20,875	313,125
VC	2	16	17	15	14,73333333	0,9375	13,8125	234,8125
VC	2	16	19	15	15,6	0,9375	14,625	277,875
VC	2	16	21	15	9,533333333	0,9375	8,9375	187,6875
VC	2	16	23	15	12,53333333	0,9375	11,75	270,25
VC	2	16	25	14	9,571428571	0,875	8,375	209,375
VC	2	16	27	14	11,07142857	0,875	9,6875	261,5625
VC	2	16	29	12	8,5	0,75	6,375	184,875
VC	2	16	31	11	5,818181818	0,6875	4	124
VC	2	16	33	11	4,090909091	0,6875	2,8125	92,8125
VC	2	16	35	11	2,636363636	0,6875	1,8125	63,4375

Suplemmentary material

VC	2	16	37	11	1,363636364	0,6875	0,9375	34,6875
VC	2	16	39	11	1,363636364	0,6875	0,9375	36,5625
VC	2	16	41	7	1,142857143	0,4375	0,5	20,5
VC	2	16	43	5	0	0,3125	0	0
VC	2	16	45	3	0	0,1875	0	0
VC	2	16	47	0	0	0	0	0
VC	3	16	3	16	5,5	1	5,5	16,5
VC	3	16	5	16	8,875	1	8,875	44,375
VC	3	16	7	16	13	1	13	91
VC	3	16	9	16	18,5	1	18,5	166,5
VC	3	16	11	16	19,875	1	19,875	218,625
VC	3	16	13	16	15,625	1	15,625	203,125
VC	3	16	15	14	10,07142857	0,875	8,8125	132,1875
VC	3	16	17	13	7,923076923	0,8125	6,4375	109,4375
VC	3	16	19	13	7,769230769	0,8125	6,3125	119,9375
VC	3	16	21	13	6,461538462	0,8125	5,25	110,25
VC	3	16	23	13	13,23076923	0,8125	10,75	247,25
VC	3	16	25	13	11,92307692	0,8125	9,6875	242,1875
VC	3	16	27	13	14,15384615	0,8125	11,5	310,5
VC	3	16	29	13	10,69230769	0,8125	8,6875	251,9375
VC	3	16	31	13	9,615384615	0,8125	7,8125	242,1875
VC	3	16	33	13	10	0,8125	8,125	268,125
VC	3	16	35	12	10,91666667	0,75	8,1875	286,5625
VC	3	16	37	12	2	0,75	1,5	55,5
VC	3	16	39	12	0,916666667	0,75	0,6875	26,8125
VC	3	16	41	11	0,636363636	0,6875	0,4375	17,9375
VC	3	16	43	9	1	0,5625	0,5625	24,1875
VC	3	16	45	7	0,428571429	0,4375	0,1875	8,4375
VC	3	16	47	3	0	0,1875	0	0
VC	3	16	49	0	0	0	0	0
VC	4	16	3	16	17,375	1	17,375	52,125
VC	4	16	5	16	13,125	1	13,125	65,625
VC	4	16	7	16	13,3125	1	13,3125	93,1875
VC	4	16	9	16	17,8125	1	17,8125	160,3125
VC	4	16	11	16	18,75	1	18,75	206,25
VC	4	16	13	15	20,66666667	0,9375	19,375	251,875
VC	4	16	15	15	16,6	0,9375	15,5625	233,4375
VC	4	16	17	15	12,2	0,9375	11,4375	194,4375
VC	4	16	19	15	14,06666667	0,9375	13,1875	250,5625
VC	4	16	21	12	25,08333333	0,75	18,8125	395,0625
VC	4	16	23	12	19,33333333	0,75	14,5	333,5
VC	4	16	25	11	19,36363636	0,6875	13,3125	332,8125
VC	4	16	27	11	16,18181818	0,6875	11,125	300,375
VC	4	16	29	11	7,636363636	0,6875	5,25	152,25
VC	4	16	31	11	6,090909091	0,6875	4,1875	129,8125

Suplemmentary material

VC	4	16	33	10	3,5	0,625	2,1875	72,1875
VC	4	16	35	10	4,1	0,625	2,5625	89,6875
VC	4	16	37	8	1,875	0,5	0,9375	34,6875
VC	4	16	39	7	1,142857143	0,4375	0,5	19,5
VC	4	16	41	5	1,2	0,3125	0,375	15,375
VC	4	16	43	5	0,4	0,3125	0,125	5,375
VC	4	16	45	2	0	0,125	0	0
VC	4	16	47	0	0	0	0	0
VC	5	16	3	16	11,625	1	11,625	34,875
VC	5	16	5	16	16,6875	1	16,6875	83,4375
VC	5	16	7	16	18,5625	1	18,5625	129,9375
VC	5	16	9	16	17,125	1	17,125	154,125
VC	5	16	11	16	24,5	1	24,5	269,5
VC	5	16	13	16	21	1	21	273
VC	5	16	15	14	23,35714286	0,875	20,4375	306,5625
VC	5	16	17	14	14,5	0,875	12,6875	215,6875
VC	5	16	19	14	18,64285714	0,875	16,3125	309,9375
VC	5	16	21	14	14,28571429	0,875	12,5	262,5
VC	5	16	23	13	15,61538462	0,8125	12,6875	291,8125
VC	5	16	25	13	15,76923077	0,8125	12,8125	320,3125
VC	5	16	27	12	7,333333333	0,75	5,5	148,5
VC	5	16	29	11	9,727272727	0,6875	6,6875	193,9375
VC	5	16	31	11	6,727272727	0,6875	4,625	143,375
VC	5	16	33	11	5,909090909	0,6875	4,0625	134,0625
VC	5	16	35	11	5,090909091	0,6875	3,5	122,5
VC	5	16	37	11	3	0,6875	2,0625	76,3125
VC	5	16	39	8	0,125	0,5	0,0625	2,4375
VC	5	16	41	6	0	0,375	0	0
VC	5	16	43	3	2,333333333	0,1875	0,4375	18,8125
VC	5	16	45	2	0	0,125	0	0
VC	5	16	47	0	0	0	0	0
VC	6	16	3	16	17,25	1	17,25	51,75
VC	6	16	5	16	14,5	1	14,5	72,5
VC	6	16	7	16	20,3125	1	20,3125	142,1875
VC	6	16	9	16	21,125	1	21,125	190,125
VC	6	16	11	16	13,3125	1	13,3125	146,4375
VC	6	16	13	16	18,375	1	18,375	238,875
VC	6	16	15	16	18,25	1	18,25	273,75
VC	6	16	17	16	21,4375	1	21,4375	364,4375
VC	6	16	19	16	11,375	1	11,375	216,125
VC	6	16	21	15	10,46666667	0,9375	9,8125	206,0625
VC	6	16	23	15	8,933333333	0,9375	8,375	192,625
VC	6	16	25	15	8,2	0,9375	7,6875	192,1875
VC	6	16	27	14	7,857142857	0,875	6,875	185,625
VC	6	16	29	14	5,357142857	0,875	4,6875	135,9375

Suplemmentary material

VC	6	16	31	14	6	0,875	5,25	162,75
VC	6	16	33	14	5,642857143	0,875	4,9375	162,9375
VC	6	16	35	14	3,785714286	0,875	3,3125	115,9375
VC	6	16	37	9	0	0,5625	0	0
VC	6	16	39	8	0	0,5	0	0
VC	6	16	41	7	0	0,4375	0	0
VC	6	16	43	6	0	0,375	0	0
VC	6	16	45	0	0	0	0	0
VC	7	16	3	16	14,125	1	14,125	42,375
VC	7	16	5	16	21	1	21	105
VC	7	16	7	16	19,8125	1	19,8125	138,6875
VC	7	16	9	16	16,25	1	16,25	146,25
VC	7	16	11	16	15,125	1	15,125	166,375
VC	7	16	13	16	13,5625	1	13,5625	176,3125
VC	7	16	15	16	11,125	1	11,125	166,875
VC	7	16	17	16	18,75	1	18,75	318,75
VC	7	16	19	15	9,8	0,9375	9,1875	174,5625
VC	7	16	21	15	9,733333333	0,9375	9,125	191,625
VC	7	16	23	15	13,2	0,9375	12,375	284,625
VC	7	16	25	15	5,666666667	0,9375	5,3125	132,8125
VC	7	16	27	15	2,666666667	0,9375	2,5	67,5
VC	7	16	29	15	3,466666667	0,9375	3,25	94,25
VC	7	16	31	14	4	0,875	3,5	108,5
VC	7	16	33	14	2,714285714	0,875	2,375	78,375
VC	7	16	35	12	4,25	0,75	3,1875	111,5625
VC	7	16	37	11	2,181818182	0,6875	1,5	55,5
VC	7	16	39	9	2,888888889	0,5625	1,625	63,375
VC	7	16	41	4	0	0,25	0	0
VC	7	16	43	4	0	0,25	0	0
VC	7	16	45	3	0	0,1875	0	0
VC	7	16	47	2	0	0,125	0	0
VC	7	16	49	0	0	0	0	0
VC	8	16	3	16	13,4375	1	13,4375	40,3125
VC	8	16	5	16	19,1875	1	19,1875	95,9375
VC	8	16	7	16	25,25	1	25,25	176,75
VC	8	16	9	16	17,375	1	17,375	156,375
VC	8	16	11	16	13,1875	1	13,1875	145,0625
VC	8	16	13	16	11	1	11	143
VC	8	16	15	16	10,25	1	10,25	153,75
VC	8	16	17	15	15,86666667	0,9375	14,875	252,875
VC	8	16	19	15	9,066666667	0,9375	8,5	161,5
VC	8	16	21	15	8,533333333	0,9375	8	168
VC	8	16	23	15	6,8	0,9375	6,375	146,625
VC	8	16	25	15	9,133333333	0,9375	8,5625	214,0625
VC	8	16	27	15	7,866666667	0,9375	7,375	199,125

Suplemmentary material

VC	8	16	29	15	6	0,9375	5,625	163,125
VC	8	16	31	15	5,133333333	0,9375	4,8125	149,1875
VC	8	16	33	15	5,8	0,9375	5,4375	179,4375
VC	8	16	35	14	1,928571429	0,875	1,6875	59,0625
VC	8	16	37	14	1,785714286	0,875	1,5625	57,8125
VC	8	16	39	13	1,384615385	0,8125	1,125	43,875
VC	8	16	41	10	0,9	0,625	0,5625	23,0625
VC	8	16	43	8	0	0,5	0	0
VC	8	16	45	5	0	0,3125	0	0
VC	8	16	47	1	0	0,0625	0	0
VC	8	16	49	0	0	0	0	0
